# Switching from controlled to assisted mechanical ventilation: a multi-center retrospective study (SWITCH)

**DOI:** 10.1186/s40635-025-00785-1

**Published:** 2025-07-16

**Authors:** Jim M. Smit, Jasper Van Bommel, Diederik A. M. P. J. Gommers, Marcel J. T. Reinders, Michel E. Van Genderen, Jesse H. Krijthe, Annemijn H. Jonkman

**Affiliations:** 1 Erasmus Medical Center, Department of Intensive Care (internal postal address: Room Ne-411), Erasmus University Medical Center, Doctor Molewaterplein 40, 3015 GD Rotterdam, The Netherlands; 2 EEMCS, Pattern Recognition & Bioinformatics Group, Delft University of Technology, Delft, The Netherlands

**Keywords:** Mechanical ventilation, Spontaneous breathing, Assisted ventilation, Hypoxemic respiratory failure, Prediction model

## Abstract

**Background:**

Switching from controlled to assisted ventilation is crucial in the trajectory of intensive care unit (ICU) stay, but no guidelines exist. We described current practices, analyzed patient characteristics associated with switch success or failure, and explored the feasibility to predict switch failure.

**Methods:**

In this retrospective study, we obtained highly granular longitudinal ICU data sets from three medical centers, covering demographics, severity scores, vital signs, ventilation, and laboratory parameters. The primary endpoint was switch success, considering a switch attempt to be successful if a patient did not return to controlled ventilation for the next 72 h while alive, and to be failed otherwise. We compared the characteristics of patients with successful vs. failed first switch attempts at ICU admission, immediately before, and 3 h after the attempt. We trained LASSO logistic regression models to predict switch failure.

**Results:**

In 4524/6715 (67%) patients attempting a switch, the first attempt failed. The first switch attempt, regardless of success or failure, was generally made at normalized PaCO_2_ and pH levels, with PEEP < 10 cmH_2_O and PaO_2_/FiO_2_ indicating mild injury. Despite very similar baseline disease severity, switch failure was associated with significantly worse outcomes, including a 28-day mortality of 27% vs. 16% and median ventilator-free days of 16 vs. 22 (*p* < 0.001). Failed attempts were initiated significantly earlier than successful ones (median 1.8 vs. 1.3 days, *p* < 0.001). Before the switch, PaO_2_/FiO_2_, if measured at PEEP > 10 cmH_2_O, and respiratory system compliance was lower in patients with switch failure (median 185 vs. 205 mmHg, *p* < 0.001; 39 vs. 41 mL/cmH_2_O, *P* = 0.001), and post-switch, patients with switch failure experienced greater deterioration in gas exchange and minimal improvement in ventilatory parameters post-switch. Contrary to our hypotheses, patient characteristics for failed vs. successful switches were surprisingly similar, resulting in prediction models with limited discriminative performance.

**Conclusions:**

Approximately two-thirds of attempts to switch patients to assisted ventilation fail, which are associated with significantly worse clinical outcomes, despite similar baseline disease severity. Contrary to our hypotheses, patients with successful and failed attempts showed similar characteristics, making switch failure difficult to predict. These findings underscore the importance of preventing switch failures and, given the retrospective nature of this study, highlight the need for prospective studies to better understand the reasons for switch failure and when spontaneous breathing can be safely initiated.

**Supplementary Information:**

The online version contains supplementary material available at 10.1186/s40635-025-00785-1.

## Background

Mechanical ventilation is essential for patients with acute respiratory failure, but often leads to secondary lung injury and inflammation, worsening outcomes [1, 2] Hence, optimizing individualized strategies for lung-protective ventilation is a key priority [3, 4] In the acute phase of respiratory failure, respiration is fully ventilator-controlled and patients are deeply sedated. Prolonged controlled ventilation delays weaning and increases the risk of complications, such as muscle weakness and delirium [2, 5–8] However, transitioning to assisted ventilation could trigger excessive breathing efforts due to high respiratory drive, [9, 10] potentially causing high lung stress, increased lung perfusion, inflammation, and ‘patient self-inflicted lung injury’ (*P*-SILI) [11]. This switch should, therefore, be initiated as early as safe, but current guidelines do not address this critical step.

This study aims to gain insights in current practices in switching from controlled to assisted ventilation, identify characteristics associated with success or failure, and assess if predictive models can accurately predict switch failure.

As we assumed that switch failures typically occur due to the patient not being ready to be switched, and that this ‘readiness’ is associated with measurable characteristics, we a priori formulated the following hypotheses: before a switch attempt, patients with failed switches have poorer gas exchange, worse respiratory mechanics, and more inflammation than those with successful switches. After the attempt, we hypothesized greater gas exchange deterioration, lacked improvement in respiratory mechanics, and further increased inflammation in failed cases compared to successful ones.

## Methods

### Study design, setting and eligibility

We utilized clinical data from three deidentified ICU data sets that were merged: our local EMC database (ICU admissions from 2017 to 2022); the Medical Information Mart for Intensive Care IV (MIMIC-IV) database (2008–2019), [12]; and the AmsterdamUMCdb (2003–2016) [13]. For further details regarding the source and granularity of each data set, see Online Appendix A.

We followed the STROBE guidelines [14] (checklist in Online Appendix B). Patients were eligible if they (1) had hypoxemic respiratory failure (at least one measured PaO_2_/FiO_2_ < 300 mmHg within the first 48 h of intubation), (2) received invasive mechanical ventilation for at least 48 h, and (3) started in controlled ventilation mode. Only the first ICU stay per hospitalization was included, with follow-up until ICU discharge or death.

### Definitions and endpoints

**Fig. 1:**
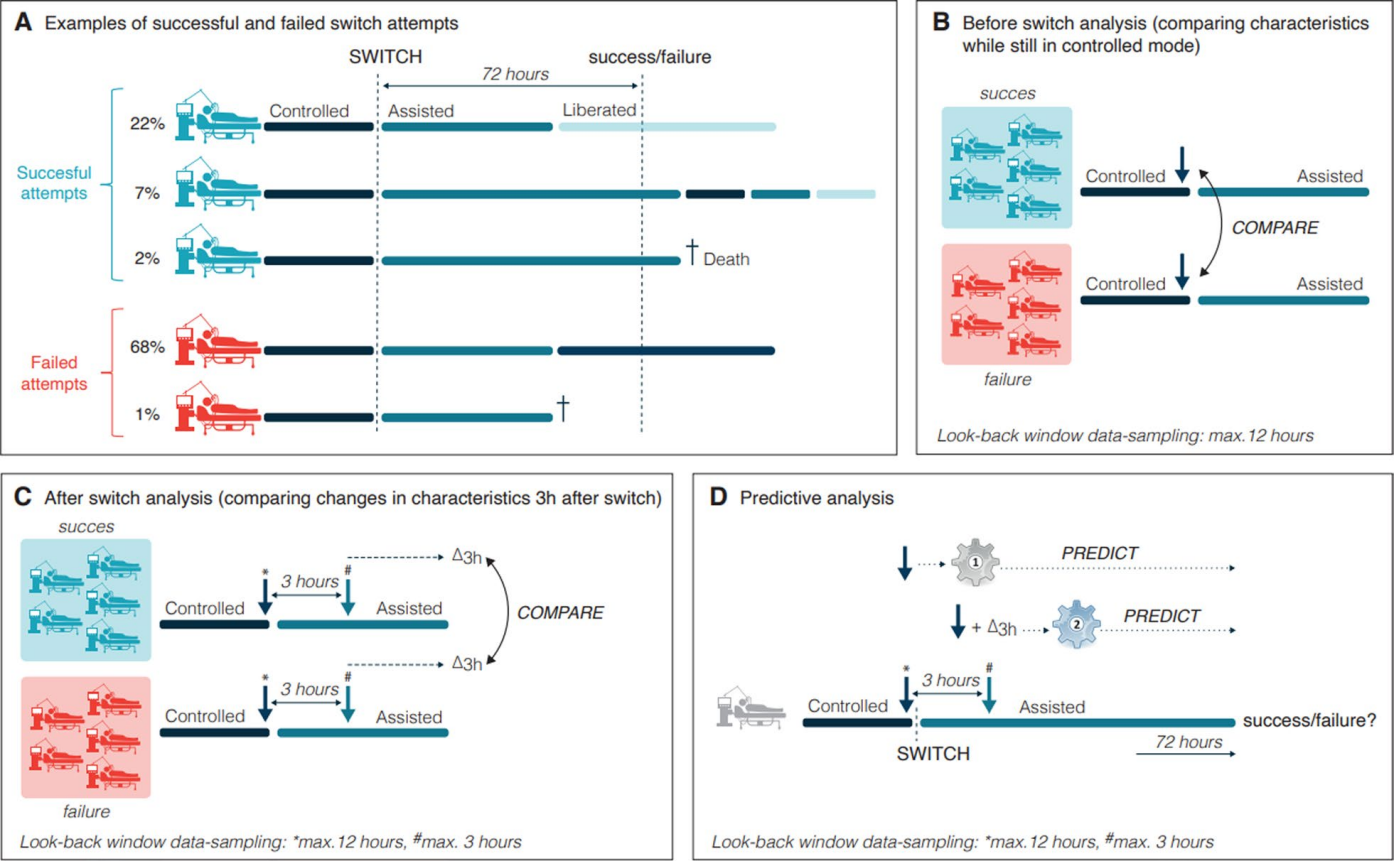
**a** Examples of ICU stays with successful and failed switch attempts. Switch success was defined as no return to controlled ventilation or death within 72 h. The different kind of trajectories and their prevalence are shown. **b**, **c** Overview of the (**b**) before switch analysis (**c**) and after switch analysis. The blue arrows represent the sampled time-varying variables. **d** Schematic overview of the predictive analysis. To investigate the potential to predict switch failure, we trained two machine learning models using LASSO regression: one designed to predict switch failure *before* the attempt (model 1), and one designed to predict switch failure 3 h after the attempt (model 2). In both models, we used variables collected prior to the switch attempt, derived from the before switch analysis. For model 2, we additionally incorporated Δ_3h_ values, as derived from the after switch analysis

We focused on the patient’s first switch attempt, i.e., the first transition from controlled to assisted ventilation. For the mapping of ventilator modes, see *2.3 Data synthesis* below. The primary endpoint was switch success, considering a switch attempt to be successful if the patient did not return to controlled ventilation for the next 72 h while alive, and to be failed otherwise (Fig. 1a). Secondary endpoints included 28-day mortality, ventilator-free days by day 28, ICU length of stay, and duration of mechanical ventilation.

### Data synthesis

#### Data collection

For each eligible patient, we extracted age, sex, baseline blood gas values, and baseline severity scores [15, 16] (if available). We collected time-varying variables measured right before the switch attempt, and within 3 h after the switch attempts, including vital signs, ventilation parameters, and lab results. Time-varying variables were available in the data sets with varying frequencies (Supplementary Table E3). Derived parameters included PaO_2_/FiO_2_, calculated from arterial PaO_2_ and the nearest prior FiO_2_; airway driving pressures (ΔP), from plateau pressure and the nearest prior positive end-expiratory pressure (PEEP); and respiratory system compliance (C_RS_), from ΔP and the nearest prior tidal volume, with ‘nearest’ meaning the closest measurement in time, but within 1 h.

#### Data pre-processing

We confirmed invasive ventilation periods and mortality times using intubation, extubation, and mortality data from clinical charts. We pre-processed uncategorized ventilation modes logged by various ventilators in different steps (full details in Online Appendix C): modes were first consolidated into four categories as per their functionalities: controlled (no patient-triggered breaths), assist-control or ‘combined’ (allowing patient-triggered breaths besides mandatory breaths), assisted (only patient-triggered breaths) and CPAP (complete mapping in Supplementary Table E4). Second, because ‘combined’ modes complicated the definition of switch attempts, we reassigned them to either controlled or assisted mode based on the nearest spontaneous respiratory rate—classifying as assisted if the rate was higher than ten breaths per minute, indicating active patient effort, and vice versa. Third, CPAP modes were reassigned to assisted mode if used during invasive ventilation, and to non-invasive/no ventilation otherwise. Fourth, transitions from controlled to assisted or assisted to controlled modes were considered only if a patient stayed in the new mode for at least 1 h. This was chosen as a pragmatic timeframe to filter out ‘false positive’ transitions stemming from abrupt mode changes that could happen, for instance, to facilitate a clinical procedure (e.g., bronchoscopy). Hence, our analysis excludes switch failures, where a patient returns to controlled mode after less than an hour in assisted mode, as these are not considered genuine switch attempts (Supplementary Figure E5c).’

### Data analyses

Data analyses were divided into four parts: we compared patient characteristics between successful and failed switch attempts at three timepoints—ICU admission, immediately before, and shortly after the switch—and evaluated the accuracy of predicting switch failure (Fig. 1b–d). Data are presented as mean (SD), median (IQR), or count (%), as appropriate. Proportions were compared using χ^2^ or Fisher’s exact test, and continuous variables using the *t* test or Wilcoxon rank-sum test. Two-sided *p* values < 0.05 were considered significant.

#### Baseline analysis

We compared baseline characteristics and endpoints between patients with successful and failed switches, and included patients who remained in controlled mode (‘no switch’) for comparison. We reported mean values for multiple measurements within 24 h. In addition, we described the time from ICU admission to the first switch attempt, the number of secondary switch attempts, and the time from the first switch attempt to failure for patients with failed switches.

#### Before switch analysis

To test our first hypothesis, we compared time-varying variables measured just *before* switch attempts for successful and failed switches (the ‘before switch analysis’, Fig. 1b). We used the most recent measurement up to 12 h before the switch attempt (‘windowed last-observation-carried-forward’, Supplementary Figure E1), and excluded variables missing for two-third of patients. We performed no further imputation. To check for potential bias, [17] we assessed whether the ‘missingness’ per variable was comparable among patients with failed and successful switch attempts. Finally, as PaO_2_/FiO_2_ and C_RS_ could vary depending on the PEEP, [18] we stratified PaO_2_/FiO_2_ and C_RS_ distributions by PEEP levels and tested if their association with switch success was significantly modified by PEEP, using a mixed effects logistic regression model [19] (Supplementary Table E1).

#### After switch analysis

To test our second hypothesis, we compared *changes* in time-varying variables 3 h after a switch attempt (Δ_3h_) between successful and failed attempts (the ‘after switch analysis’, Fig. 1c). Patients who failed the switch or were liberated from mechanical ventilation within 3 h were excluded. To calculate Δ_3h_, we subtracted the most recent measurement pre-switch with the most recent variable post-switch measurement 3 h after the attempt. Only variables with Δ_3h_ data for at least one-third of patients were included. We selected this 3-h window, because with shorter timeframes, new measurements of key predictors like blood gas values are often unavailable, while longer windows would exclude many patients who had already failed the switch (Supplementary Figure E6).

#### Predictive analysis

A model that accurately predicts switch success or failure could aid physicians in deciding whether to switch patients to an assisted mode. In addition, if switch failure could be predicted shortly after the switch attempt (i.e., after 3 h), a longer duration in assisted mode, while the patient is not ready for it, could be prevented. Therefore, we trained two machine learning models using LASSO regression [20]. The first model (‘Model 1’) predicted switch failure before the attempt, using pre-switch variables. The second model (‘model 2’) predicted failure 3 h after the switch, using both pre-switch variables and changes post-switch (Δ_3h_ values; Fig. 1d). We evaluated both models using the area under the ROC curve (AUC) and analyzed the contribution of different variable groups (details in Online Appendix D).

#### Sensitivity analyses

Several sensitivity analyses were performed (details in Online Appendix E): first, since mortality is included in the definition of a failed switch (see Sect."Definitions and endpoints"), we repeated the baseline analysis (which included findings on mortality), only including patients who survived at least 72 h after the first switch attempt. Second, to assess generalizability across data sets, we compared findings across the three data sets. Third, due to our pre-processing of combined ventilator modes (see Sect."Data synthesis"), identified switch attempts could be either actual mode changes or respiratory rate adjustments during combined modes. We compared findings between these ‘types’ separately. Fourth, we compared the findings in the baseline, before switch, and after switch analyses for patients with ‘early’ vs. ‘late’ switch failures, splitting patients by the median time to failure. Fifth, as the after switch analysis excluded patient who already failed their switch within 3 h post-switch, potentially influencing the findings, we repeated the it considering changes in time-varying variables from 1 to 8 h post-switch (i.e., Δ_1h_ to Δ_8h_). Sixth, we explored the robustness of the predictive analysis by (1) evaluating the added value of a flexible, non-linear Light Gradient Boosting Machine model (LightGBM), (2) testing sensitivity to the imputation method using scikit-learn’s IterativeImputer, and (3) restricting the analysis to patients with PaO₂/FiO₂ measurements taken at PEEP levels above 10 cmH₂O.

## Results

### Baseline analysis

**Fig. 2:**
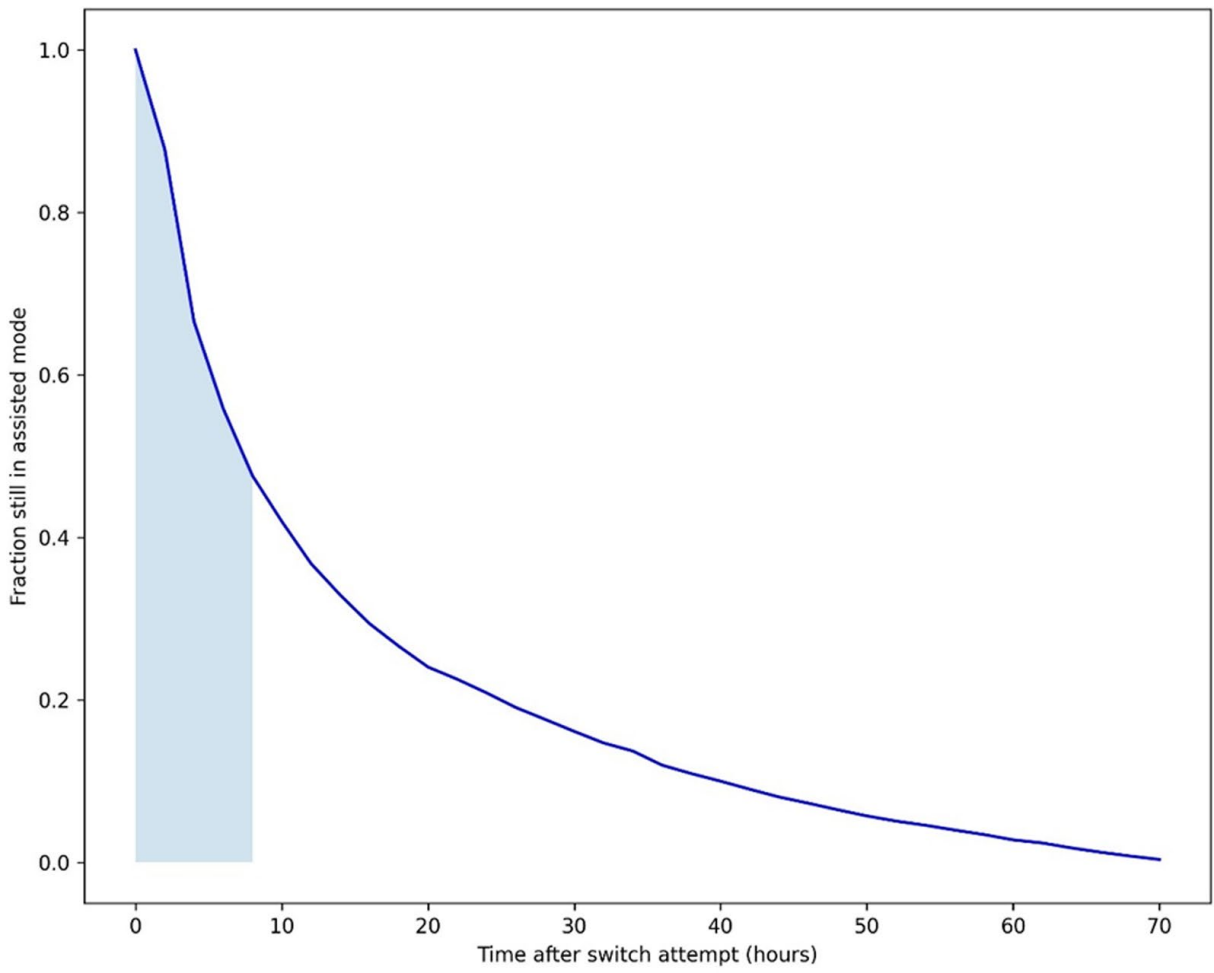
Fraction of patients still in assisted mode at different follow-up times after the first switch attempt among patients with a failed switch attempt (*n* = 4720). Half of the attempts failed within 8 h after the switch attempt (shaded area)

Across the three databases, 7277 patients met the inclusion criteria (see Supplementary Figure E2). Of these, 6715 (92%) underwent a switch attempt, with 2191 (33%) being successful (Fig. 1a). Success rates ranged from 26 to 37% across centers. Most switch failures occurred due to a transition back to controlled mode within 72 h (Fig. 1a). Patients with failed attempts had significantly worse outcomes, including higher 28-day mortality (27% vs. 16%), longer median ICU stays (9.9 vs. 7.8 days), extended mechanical ventilation duration (6.9 vs. 4.8 days), and fewer median VFDs (16.3 vs. 22.2 days), despite comparable baseline characteristics and severity scores (Table 1). Switch attempts generally occurred early after ICU admission, but later in successful cases (median 1.8 vs. 1.3 days after ICU admission, *p* < 0.001), a finding that was consistent across the data sets (Supplementary Tables E7–9). Among failed attempts, failure occurred after a median of 8 h (IQR: 4–19) (Fig. 2). Failed attempts were often followed by additional attempts (median: 2, IQR: 1–4). The 562 patients (8%) without attempts had worse baseline characteristics, higher severity scores, worse secondary outcomes, and high mortality rate (82%; Supplementary Table E2).

**Table 1:** Results of the baseline analysis. Baseline characteristics and endpoints of the full cohort, grouped by the success or failure of the first switch attempt

Variable	Successful switch (*n* = 2191)	Failed switch (*n* = 4524)	*P* value
Demographics			
Age group, *n* (%)			
18–39	177 (8)	419 (9)	0.12
40–49	202 (9)	413 (9)	0.928
50–59	357 (16)	784 (17)	0.299
60–69	462 (21)	979 (22)	0.612
70–79	469 (21)	915 (20)	0.274
80 +	254 (12)	559 (12)	0.38
Female sex (%)	821 (37.5)	1681 (37.2)	0.957
Gas exchange			
PaO_2_/FiO_2_	216 (163–278)	210 (156–278)	0.395
PaO_2_ (mmHg)	111.0 (91.4–141.7)	111.9 (91.8–143.1)	0.638
PaCO_2_ (mmHg)^‡^	40.0 (36.5–44.3)	40.5 (36.4–45.1)	0.001
pH^††^	7.35 (7.31–7.4)	7.34 (7.29–7.39)	< 0.001
Respiratory mechanics			
ΔP (cmH_2_O)^††^	11.7 (9.8–14.0)	12.1 (10.0–14.6)	< 0.001
C_RS_ (mL/cmH_2_O)^††^	40.6 (32.3–50.5)	38.8 (31.2–48.5)	0.329
SOFA components			
Mean arterial pressure (mmHg)^†^	73.9 (67.3–81.9)	73.1 (66.9–80.5)	0.037
Bilirubin (µmol/L)^††^	11.0 (6.8–20.3)	11.5 (6.8–21.0)	0.088
Creatinine (µmol/L)^††^	97.2 (73.3–139.2)	100.8 (74.0–151.1)	0.072
Platelet count (10^9^/L)	191.5 (135.0–256.0)	185 (127.2–250.7)	0.959
Baseline severity scores			
APACHE-II score^†^	26.0 (21.0–32.0)	26.0 (20.0–32.0)	0.452
SAPS-II score^†^	44.0 (34.5–54.0)	46.0 (38.0–57.0)	< 0.001
Secondary endpoints			
28-day mortality (%)^‡^	367 (16)	1259 (27)	< 0.001
VFDs-28 (days)^‡^	22.2 (12.4–25.2)	16.3 (0.0–22.8)	< 0.001
Length of MV (days)	4.8 (2.7–8.9)	6.9 (3.9–12.6)	< 0.001
Length of ICU stay (days)	7.8 (4.8–13.6)	9.9 (5.9–17.2)	< 0.001
Switch characteristics			
Time between ICU admission and switch attempt (days)^‡^	1.8 (0.8–2.9)	1.3 (0.5–2.5)	< 0.001
Time between switch attempt and switch failure (hours)	–	8 (4–19)	–
Number of secondary switch attempts (n)	–	2 (1–4)	–

Data are in median (IQR) or number (percentage). †/†† Results were based on only two (††) or one (†) of the three included data sets. ‡Variable statistically significantly associated with treatment failure, showing associations in consistent direction in all three included data sets. PaO_2_ = arterial oxygen pressure, PaCO_2_ = Partial pressure of carbon dioxide, ΔP = driving pressure, C_RS_ = respiratory system compliance, VFDs = ventilator-free days, MV = mechanical ventilation, ICU = intensive care unit

### Before switch analysis

**Fig. 3:**
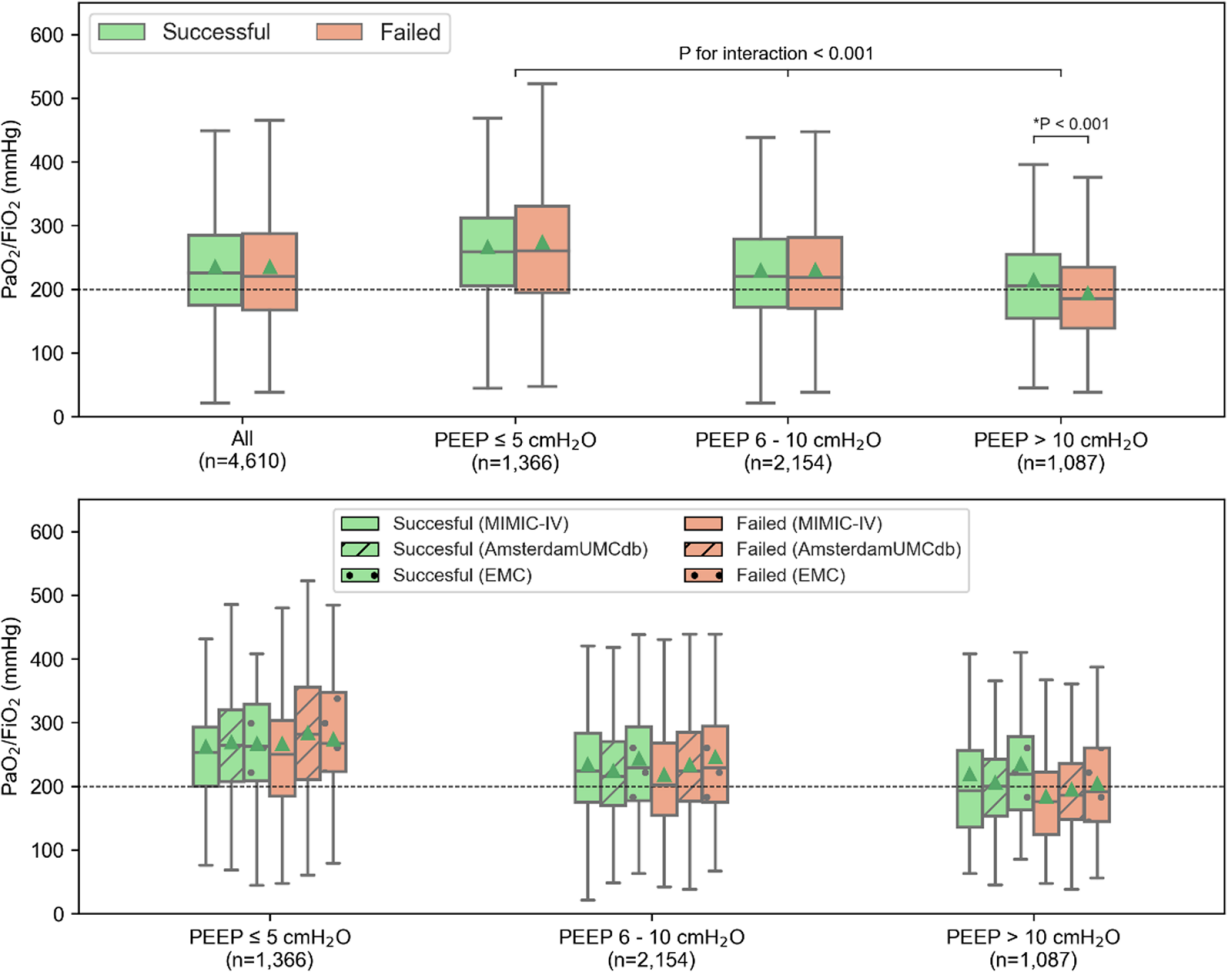
Boxplots of the distributions of the PaO_2_/FiO_2_ measurements at the switch attempts, stratified for the corresponding set PEEP (upper plot) and further stratified for data set (lower plot). Distribution means are depicted with the green triangles. The *P* values for interaction denote whether the PEEP significantly modified the association between the variable and switch success

Among patients with a switch attempt, 22 time-varying variables were sampled with sufficient availability (Table 2, Supplementary Figure E3). Regardless of success or failure, the first switch attempt occurred at varying PaO_2_/FiO_2_ levels, typically showing improvement towards mild injury, with normalized PaCO_2_ and pH, and PEEP below 10 cmH_2_O in most cases. Patients with failed attempts generally had worse gas exchange and higher ventilatory parameters before the attempt, including lower base excess and pH, and higher FiO_2_, lactic acid, and respiratory pressures (*p* < 0.001). While these findings align with our hypotheses, most differences are small, and some variables (eg, PaO_2_) showed opposite trends than expected. Failed attempts showed slightly lower PaO_2_/FiO_2_ values, but this association was significantly modified by the set PEEP—consistently across all databases: at low or moderate PEEP (≤ 10 cmH_2_O), PaO_2_/FiO_2_ values were similar between successful and failed attempts. However, at higher PEEP (> 10 cmH_2_O), PaO_2_/FiO_2_ was significantly lower in failed attempts (median 185 mmHg) compared to successful ones (median 205 mmHg, *p* value for interaction < 0.001; Fig. 3). Failed attempts were also linked to lower C_RS_, but this was not significantly influenced by PEEP level (*p* value for interaction = 0.42).

**Table 2:** Results of the before switch analysis

Variable	Successful switch (*n* = 2191)	Failed switch (*n* = 4524)	*P* value	Missingness (% successful, % failed)
Gas exchange parameters				
PaO_2_ (mmHg)	98.0 (81.2; 122.0)	99.0 (82.5; 125.0)	0.002	8, 10
PaCO_2_ (mmHg)	40.0 (36.0; 44.0)	40.0 (35.0; 45.0)	0.123	8, 10
PaO_2_/FiO_2_				23, 30
All	225 (174; 284)	220 (167; 288)	0.937	
Measured at PEEP ≤ 5 cmH_2_O	258 (205; 312)	260 (194; 332)	0.304	
Measured at PEEP 6–10 cmH_2_O	220 (172; 279)	218 (170; 280)	0.657	
Measured at PEEP > 10 cmH_2_O^‡^	205 (154; 254)	185 (138; 234)	< 0.001	
pH^††^	7.39 (7.34; 7.43)	7.38 (7.33; 7.43)	< 0.001	20, 20
Base excess (mmol/L)^‡^	1.0 (− 1.2; 3.8)	0.0 (− 3.0; 3.0)	< 0.001	8, 10
Lactic acid (mmol/L)^‡^	1.5 (1.1; 2.2)	1.7 (1.2; 2.7)	< 0.001	35, 33
HCO_3_^–^ (mmol/L)^††^	23.0 (20.3; 25.7)	22.8 (20.0; 25.4)	0.073	18, 17
FiO_2_ (%)^‡^	41 (40; 50)	41 (40; 50)	< 0.001	0, 0
SpO_2_ (%)	98 (96; 99)	98 (96; 99)	0.215	0, 0
Ventilatory parameters				
Pplat (cmH_2_O)^††^	19.0 (16.0; 22.0)	20.0 (17.0; 23.0)	< 0.001	54, 47
ΔP (cmH_2_O)^††^	11.0 (9.0; 13.0)	12.0 (9.0; 14.0)	< 0.001	55, 47
Pmean (cmH_2_O)^†^	11.0 (9.0; 13.0)	12.0 (9.0; 14.0)	< 0.001	56, 46
Ppeak (cmH_2_O)	22.0 (19.0; 26.0)	23.0 (19.0; 27.0)	< 0.001	0, 0
PEEP (cmH_2_O)	8.0 (5.0; 10.0)	8.0 (5.0; 10.0)	0.692	1, 2
Respiratory rate (breaths/min)	18 (15; 22)	19 (16; 23)	< 0.001	0, 0
Minute volume (L/min)^††^	8.8 (7.4; 10.4)	8.9 (7.4; 10.7)	0.017	12, 10
C_RS_ (mL/cmH_2_O)^††^				55, 48
All	41 (33; 52)	39 (31; 50)	0.001	
Measured at PEEP ≤ 5 cmH_2_O	40 (32; 50)	38 (30; 47)	0.018	
Measured at PEEP 6–10 cmH_2_O	42 (33; 52)	39 (31; 51)	0.061	
Measured at PEEP > 10 cmH_2_O	43 (34; 58)	42 (33; 56)	0.096	
Inflammatory markers				
White cell count (10^9^/L)^††^	11.9 (8.9; 16.4)	12.1 (8.7; 17.2)	0.06	29, 27
Other parameters				
Heart rate (bpm)^††^	84 (72; 96)	85 (74; 99)	< 0.001	12, 10
Temperature (°C)	37.0 (36.7; 37.3)	37.0 (36.6; 37.4)	0.709	23, 23
Mean arterial pressure (mmHg)^††^	79 (70; 88)	78 (69; 88)	0.06	22, 25

Data are in median (IQR). †/†† Results were based on only two (††) or one (†) of the three included data sets. ‡Variable statistically significantly associated with treatment failure, showing associations in consistent direction in all three included data sets. PaO_2_ = arterial oxygen pressure, PaCO_2_ = partial pressure of carbon dioxide, PEEP = positive end-expiratory pressure, HCO_3_^−^ = bicarbonate, FiO_2_ = fraction of inspired oxygen, SpO_2_ = oxygen saturation, Pplat = plateau pressure, ΔP = driving pressure, Pmean = mean airway pressure, Ppeak = peak airway pressure, C_RS_ = respiratory system compliance

Time-varying variables sampled at the moment of a switch attempt (i.e., switch samples)

### After switch analysis

For patients still in assisted mode 3 h after the switch attempt (5620/6715; 83.7%), we collected Δ_3h_ values for 16 time-varying variables with sufficient data available (Table 3, Supplementary Figure E4). Patients with failed attempts showed greater deterioration in gas exchange and less reduction in ventilatory parameters, such as a larger increase in PaCO_2_, a greater drop in pH and PaO_2_/FiO_2_, and smaller reductions in peak pressures (*p* < 0.05), though differences were minor.

**Table 3:** Results of the after switch analysis. Δ_3h_ values of the included time-varying variables

Variable	Successful switch (*n* = 2100)	Failed switch (*n* = 3520)	*P* value	Missingness (% successful, % failed)
Gas exchange parameters				
Δ_3h_ PaO_2_ (mmHg)	− 6.1 (40.8)	− 8.5 (45.0)	0.181	55, 59
Δ_3h_ PaCO_2_ (mmHg)^‡^	0.2 (5.6)	0.8 (6.6)	0.012	55, 59
Δ_3h_ PaO_2_/FiO_2_^‡^	− 2 (74)	− 12 (89)	0.029	70, 76
Δ_3h_ pH^††^	− 0.001 (0.046)	− 0.006 (0.054)	0.018	63, 66
Δ_3h_ Base excess (mmol/L)	0.1 (1.6)	− 0.1 (2.1)	0.074	56, 59
Δ_3h_ FiO_2_ (%)	− 1 (9)	− 1 (11)	0.169	31, 47
Δ_3h_ SpO_2_ (%)	0 (4)	− 1 (4)	0.024	1, 1
Ventilatory parameters				
Δ_3h_ Ppeak (cmH_2_O)	− 3.2 (5.1)	− 2.7 (5.2)	0.005	33, 49
Δ_3h_ PEEP (cmH_2_O)^††^	− 0.4 (1.7)	− 0.2 (1.6)	< 0.001	33, 49
Δ_3h_ Respiratory rate (breaths/min)	− 1 (7)	− 1 (7)	0.582	0, 1
Δ_3h_ Minute volume (L/min)^††^	− 0.2 (3.7)	− 0.5 (5.3)	0.074	44, 57
Δ_3h_ Tidal volume (mL)^††^	48 (546)	34 (433)	0.429	33, 49
Other parameters				
Δ_3h_ Heart rate (bpm)^††^	3 (12)	3 (13)	0.513	12, 10
Δ_3h_ Temperature (°C)	0.1 (0.8)	0.1 (0.8)	0.227	58, 63
Δ_3h_ Mean arterial pressure (mmHg)^††^	0 (16)	0 (16)	0.798	23, 27

Data are mean (SD). †/†† Results were based on only two (††) or one (†) of the three included data sets. ‡Variable statistically significantly associated with treatment failure, showing associations in consistent direction in all three included data sets. PaO_2_ = arterial oxygen pressure, PaCO_2_ = Partial pressure of carbon dioxide, PEEP = positive end-expiratory pressure, FiO_2_ = Fraction of inspired oxygen, SpO_2_ = oxygen saturation, Pplat = pleateau pressure, ΔP = driving pressure, Ppeak = peak airway pressure

Although variables’ availability differed across the three data sets, missing data were similar between patients with failed and successful switch attempts, limiting bias due to our complete case analysis approach.

### Predictive analysis

Prediction of switch failure, both *before* and shortly *after* the switch attempt, yielded limited discriminative performance, with a cross-validated AUC of 0.58 and 0.61 for model 1 and 2, respectively. In both models, the gas exchange parameters measured before the switch attempt contributed most to the predictive performance (for details, see Online Appendix D).

### Sensitivity analyses

Even when limited to patients who survived at least 72 h after the switch attempt, those with failed switches experienced significantly worse clinical outcomes, including higher 28-day mortality (21% vs. 14%; Supplementary Table E6). Results for the different analyses were similar across the three included data sets (Supplementary Tables E7–15), and associations between variables and outcomes were often in consistent direction across data sets (highlighted using a “‡”, Tables 1, 2, and 3).

5492/6715 (82%) of the switch attempts were observed as an actual mode switch (controlled to assisted mode) and only 18% of switches were observed as a change in respiratory rate during a combined mode. For both ‘types’ of switch attempts, we observed similar associations for most of the variables that had an overall statistically significant association with switch failure (Supplementary Tables E16–21). Notably, failed switch attempts observed as a change in respiratory rate during a combined mode, failed earlier compared to the failed switch attempts from controlled to assisted modes (median of 5 vs. 9 h).

Early (i.e, within 8 h) and late (i.e., after 8 h) failures exhibited similar baseline characteristics and clinical outcomes (Supplementary Table E22). Compared to late failures, early failures were characterized by slightly worse ventilatory parameters before the switch attempt, and a bigger increase in PaCO_2_ and a bigger drop in pH shortly after the switch attempt (*p* < 0.01) (Supplementary Tables E23, E24). Because the differences for Δ-PaCO_2_ and Δ-pH between successful and failed switch attempts were mostly driven by the early failures, these differences disappear at later follow-up times (Supplementary Figure E10). Supplementary Table E25 shows the results of the sensitivity analyses assessing the robustness of the predictive model. Performance was slightly worse with LightGBM compared to LASSO regression, and remained similar when LASSO was used with an alternative imputation method or limited to patients with PaO₂/FiO₂ measured at PEEP > 10 cmH₂O—despite stronger associations with switch failure in the pre-switch analysis (Table 2).

## Discussion

### Principal findings

In this large (> 7000 patients) international three-cohort retrospective study, our main findings are that (1) most of the first switches from controlled to assisted ventilation fail (67%) and these patients have poorer clinical outcomes (regardless of the failure occurring early or late after the switch) compared to successful first switch attempts (28-day mortality 27% vs. 16%, median VFDs-28 16 vs. 22 days), despite similar baseline characteristics and baseline disease severity. This suggests that the failed switch attempt itself may contribute to negative outcomes (though causality remains unclear), and emphasizes the importance of improving the ability to accurately *predict* switch success; (2) the first switch attempt, regardless of success or failure, was generally made at normalized PaCO_2_ and pH levels, with PEEP < 10 cmH_2_O and PaO_2_/FiO_2_ indicating mild injury. Switches occurred early after admission, with failed attempts even earlier than successful ones, and (3) although patients with failed switch attempts had poorer gas exchange and ventilatory parameters before the attempt, and experienced greater deterioration afterwards, we found characteristics around the switch to be surprisingly similar between successful and failed attempts. This resulted in limited performance in predicting switch success using machine learning models.

The unexpected similarity between patients with successful and failed switch attempts and poor predictive model performance, may stem from several factors. First, limitations inherent to retrospective studies (i.e., missingness of measurements) may have attenuated existing associations between patient characteristics and switch failure. Second, patient characteristics unexamined/unavailable in this study might be important predictors of switch failure, for instance respiratory drive/effort (see 4.2 Related works below). Third, the reasons behind physicians’ decisions to return to controlled ventilation were not recorded and could also include non-respiratory factors which may have weakened associations between patient characteristics and switch failure. Fourth, the execution of the switch attempt itself may also be a factor; if poorly conducted (e.g., by insufficient titration of sedatives), it could result in switch failure, even if the patient may have been ready to be switched. Finally, even if all relevant variables would be available for analysis, controlled ventilation may ‘mask’ characteristics informative for readiness to start spontaneous breathing, and hence, patients’ readiness to be switched may simply be fairly unpredictable.

### Related work

While the importance of early spontaneous breathing initiation is increasingly recognized [21–25] research on clinical and physiological patterns during this critical phase remains limited, often based on small ICU sub-populations [26–30]. Studies in COVID-19 cohorts [26, 27] proposed similar definitions for switch success/failure and found that failure was associated with adverse outcomes, aligning with our findings. However, they reported lower failure rates (31–44% vs. 69%), which might be underestimated because of only once-daily ventilator data collections [26, 27] instead of using detailed longitudinal data enabling more precise analysis [26, 27]. In addition, Balzani et al. [31] reported that patients with prolonged sedation and those with COVID-19 were more susceptible switch failure. Only 3 out of 48 patients were put back to controlled ventilation, while other ‘failure’ patients (*n* = 9) received more sedation while remaining on assisted ventilation, challenging the definitions. Another study on COVID-19 patients by Haudebourg et al. [32] found a slightly lower switch failure rate (57% vs. our 67%), using the same definition for failure. They also reported a very similar time to failure among those who failed, with a median of 9 h compared to our study’s median of 8 h.

Both Perez et al. [27] and Polo Friz et al. [26] identified low PaO_2_/FiO_2_ before the switch as independent predictor of failure [26, 27]. We observed this trend *only* for PaO_2_/FiO_2_ measured at higher PEEP (> 10 cmH_2_O), with a median of 205 mmHg vs. 185 mmHg for successful vs. failed switches (Table 2, Fig. 3). This suggests that the predictive role of PaO_2_/FiO_2_ for switch failure depends on the applied PEEP level at the time of measurement. The relatively higher PEEP levels reported by Polo Friz et al. [26] compared to our cohort (11.5 cmH_2_O vs. 8.5 cmH_2_O in our work) support this hypothesis. This also highlights the importance of investigating interaction of parameters in their association with switch failure [33].

Developing ‘actionable’ models using causal inference techniques [34–37] could generate further hypotheses for better switch strategies, potentially conditional on patient characteristics. Shahn et al. [30] performed a ‘target trial emulation’ [38] to study switch timing strategies, suggesting benefits from earlier switches after ICU admission. However, we showed that failed switch attempts occurred earlier in the ICU stay than successful ones, a pattern observed consistently across the three data sets. Although our findings do not imply causal relationships, the (modest) associations we found could guide future target trial emulations into switch strategies, particularly those focused on time-varying gas exchange (considering PEEP levels) and respiratory mechanics parameters. In line with this reasoning, using the WEAN–SAFE database [7], Reep et al. [39] indicated that it might be useful to switch to assisted ventilation with PaO_2_/FiO_2_ > 150 mmHg. This is an easy to implement oxygenation threshold; however, the interaction of PEEP and PaO_2_/FiO_2_ was not considered and may be important as previously noted. In addition, only once-daily data collections where available in the WEAN-SAFE database, while we illustrate that most switch failures occurred within 1 day after the switch (50% failures within 8 h). Hence, switch failures or clinical parameters around this exact moment may have been missed.

Current literature, including data used in our work, lack comprehensive information on breathing effort (e.g., esophageal or occlusion pressures) and patient–ventilator asynchrony, which would be crucial for understanding physiological responses around the switch that are potentially associated with failure, beyond measures of gas exchange. Explorative small studies utilizing advanced monitoring and/or biomarker assessment suggested that estimations (but no quantification was done) of drive and effort were related to switch failure [31], and that the magnitude of pendelluft (measured on EIT) had an association with inflammatory biomarkers [40]. In line with these hypotheses, we are currently conducting an in-depth physiological intervention study aimed to further unravel the (patho) physiology around this important switch moment, using multi-modal monitoring techniques (NCT06438198 [41]).

### Strengths, limitations and future directions

This study is the first to detail international clinical practice, patient characteristics, and the within-patient effects of various factors during the transition from controlled to assisted ventilation using highly granular multi-cohort data. The topic is characterized by substantial variation in practice and a lack of consensus in terminology. To foster comparability across research, we proposed clear definitions for a switch attempt and switch success, alongside strategies to handle assisted-control (i.e., combined) ventilator modes and abrupt mode transitions. We focused on patients with hypoxemic respiratory failure who were invasively ventilated for at least 2 days, excluding those with low weaning difficulty risk, [42, 43] using data from the entire ICU stay.

This study also has limitations. We only considered first switch attempts, so the results may not extend to follow-up attempts. Some findings are based on data from only one or two of the three included data sets due to variations in data availability. In the MIMIC-IV and EMC data sets, the relatively low frequency of ventilator mode logging could have caused delays between the actual moment of switching and the moment of detection in our analysis, yet data are more granular than in other studies [26, 27, 39]. Given the consistency of key findings across data sets, particularly the AmsterdamUMCdb, where logging was most frequent, we expect the impact of this limitation to be minimal. Although we hypothesized that patients with failed switch attempts would exhibit greater inflammation than those with successful switches, the available data sets only provided data for WBC counts and no other inflammatory markers, leaving this hypothesis largely untested. In addition, the use of only three centers may not fully capture global practices. Last, despite the lack of data on breathing effort, this study remains of importance in evaluating clinical variables prior to the switch, while the patient is still on controlled ventilation (no patient effort) to guide switch initiation.

## Conclusions

This international three-cohort retrospective study of over 7000 intubated patients analyzed the clinical and physiological characteristics during the transition from controlled to assisted ventilation. Notably, more than two-thirds of initial switch attempts failed, associated with worse outcomes compared to successful attempts, despite similar baseline disease severity. We found an unexpected similarity in patient characteristics at baseline, before and after switch attempts, whether successful or failed, making switch success hard to predict. Prospective studies with detailed physiological and clinical assessments are crucial for understanding when to safely initiate spontaneous breathing during mechanical ventilation.

## Supplementary Information

Additional file 1.

## Data Availability

Access to the MIMIC-IV database may be requested via: https://mimic.physionet.org/. Access to the AmsterdamUMCdb database may be requested via https://amsterdammedicaldatascience.nl/amsterdamumcdb/.
