# Supplementary material for: Switching from controlled to assisted mechanical ventilation: a multi-center retrospective study (SWITCH)
Source: Intensive Care Med Exp. 2025 Jul 16;13:73. doi: 10.1186/s40635-025-00785-1 (PMC12267752; doi:10.1186/s40635-025-00785-1)

**Online Data Supplement**Switching from Controlled to Assisted Mechanical Ventilation: a Multi-center Retrospective study
J.M. Smit, J. Van Bommel, D.A.M.P.J. Gommers, M.J.T. Reinders, M.E. Van Genderen, J.H. Krijthe, A.H. Jonkman

- **Supplementary Table and Figures** (p.2-5)
- **Appendix A**: Details of included databases (p.6-8)
- **Appendix B**: STROBE statement checklist (p.9-10)
- **Appendix C**: Data pre-processing (p.11-17)
- **Appendix D**: Predictive analysis (p.18-21)
- **Appendix E**: Sensitivity analyses (p.22-57)

Table E1: The R implementation (using the lme4 package) for the linear mixed-effects logistic regression models (LMMs) used to perform the interaction test.

The term “variable” denotes either PaO_2_/FiO_2_ or C_RS_, and “dataset” denotes the subgroup variable based on the dataset the datapoint was from (ie, MIMIC-IV, AmsterdamUMCdb or EMC).

| Model to .. | R Implementation |
| --- | --- |
| perform the interaction test to test whether the association of PaO_2_/FiO_2_ and C_RS_ with switch success was significantly modified by the set PEEP | formula_interaction <- "switch_success ~ set_peep*variable + (1 \| dataset)"  lmm <- glmer(formula_interaction, data = data, family = binomial) |

Table E2: Baseline characteristics and endpoints of patients without a switch attempt. Data are in median (IQR) or number (percentage). †/†† Results were based on only two (††) or one (†) of the three included datasets. PaO2=arterial oxygen pressure, PaCO2=Partial pressure of carbon dioxide, ΔP=driving pressure, CRS=respiratory system compliance, VFDs=ventilator-free days, MV=mechanical ventilation, ICU=intensive care unit.

| Variable | No switch attempt  (n=562) |
| --- | --- |
| **Demographics** |  |
| Age group, n (%) |  |
| 18-39 | 177 (8) |
| 40-49 | 202 (9) |
| 50-59 | 357 (16) |
| 60-69 | 462 (21) |
| 70-79 | 469 (21) |
| 80+ | 254 (12) |
| Female sex (%) | 821(37.5) |
| **Gas exchange** |  |
| PaO_2_/FiO_2_ | 194 (135 - 272) |
| PaO_2_ (mmHg) | 111.2 (88.6 - 140.0) |
| PaCO_2_ (mmHg) | 40.5 (35.5 - 45.8) |
| pH^††^ | 7.30 (7.24 - 7.36) |
| **Respiratory mechanics** |  |
| ΔP (cmH_2_O)^††^ | 12.8 (10.5 - 15.0) |
| C_RS_ (mL/cmH_2_O)^††^ | 36.8 (29.8 - 46.2) |
| **SOFA components** |  |
| Mean arterial pressure (mmHg)^†^ | 71.1 (65.0 - 80.7) |
| Bilirubin (µmol/L)^††^ | 12.8 (7.0 - 27.0) |
| Creatinine (µmol/L)^††^ | 130.8 (85.5 - 194.5) |
| Platelet count (10^9^/L) | 172.3 (110.5 - 238.9) |
| **Baseline severity scores** |  |
| APACHE-II score^†^ | 33.0 (26.0 - 39.0) |
| SAPS-II score^†^ | 53.5 (43.2 - 66.8) |
| **Secondary Endpoints** |  |
| 28-d mortality (%) | 464 (82) |
| VFDs-28 (days) | 22.2 (12.4 - 25.2) |
| Length of MV (days) | 4.8 (2.7 - 8.9) |
| Length of ICU stay (days) | 7.8 (4.8 - 13.6) |

Figure E1: Overview of the windowed last-observation-carried-forward strategy. Time-varying variables were sampled, selecting the most recent measurement (represented by the orange circles) at the moment of sampling (situation ii), specifying a maximum ‘look-back window’. If no measurement was available within this window, the variable was considered missing and no imputation was performed (situation i). For the samples at the moment of the switch attempts, we used a look-back window of twelve hours, whereas for the follow-up samples (ie, the Δ_3h_ values), the look-back windows were chosen such that only measurements were carried forward which occurred after the switch attempt (ie, using a look-back window of three hours).

**
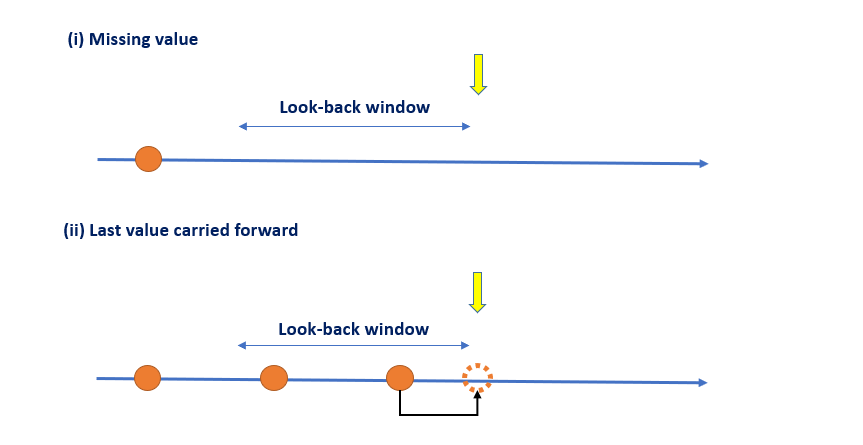
**

Figure E2: Flowchart describing the inclusion of eligible patients across the three datasets.


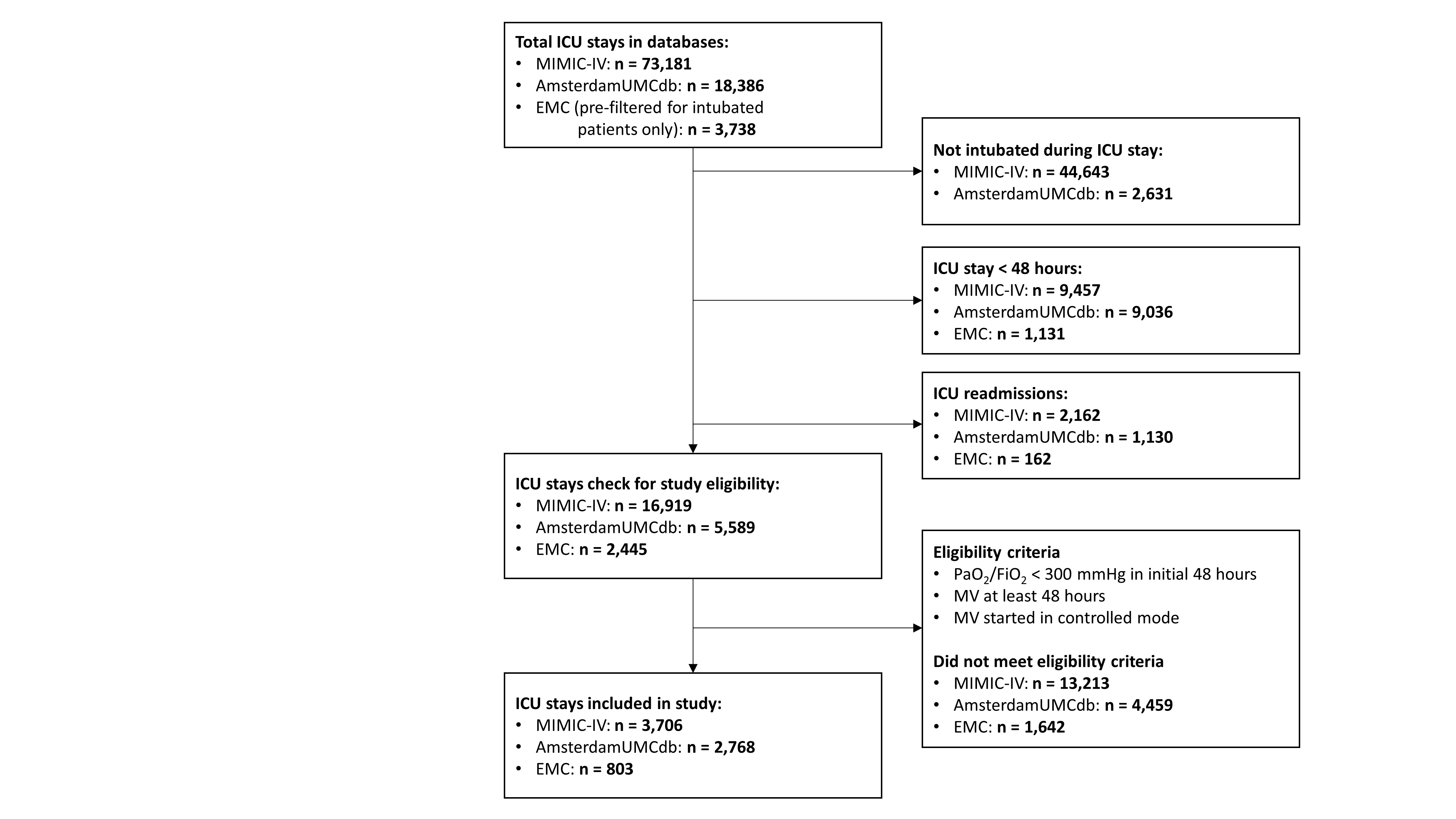


**Appendix A:** Included databases

The EMC database comprised of admissions from 2017 to 2022. The MIMIC-IV database contains records from ICU stays between 2008 and 2019 to Beth Israel Deaconess Medical Center, a tertiary academic medical center in Boston, MA, USA. Institutional review boards at both centers approved the use of the data for research and granted a waiver of informed consent. The AmsterdamUMCdb contains high-resolution clinical data related to ICU admissions from 2003 to 2016.[1] Among the included patients, we calculated the number of measurements for each variable per day. Table E3 shows the mean number of measurements among the included patients in total, and for each dataset separately. The Amsterdamumcdb was the most granular dataset, with the highest average daily measurements for most of the variables.

The ventilator modes showed a wide variation in the average number of measurements per day across the different datasets, with the AmsterdamUMCdb dataset having more than 40 times the frequency of the others (Table E3). A histogram of measurement frequencies within the AmsterdamUMCdb dataset reveals a highly skewed distribution (Figure E3). This skew is primarily due to about 21% of patients for whom ventilator modes were logged every minute during a certain period of their ICU stay. Excluding these patients, the frequency of ventilator mode logging across datasets is more comparable. The MIMIC-IV dataset has the lowest frequency (logged every 3 to 4 hours), the AmsterdamUMCdb dataset the highest (logged roughly every hour), and the EMC dataset contains both low and high-frequency subgroups.

Table E3: Mean number of measurements per day for the different databases. PaO_2_=arterial oxygen pressure, PaCO_2_=Partial pressure of carbon dioxide, PEEP= Positive end-expiratory pressure, HCO_3_^-^ = bicarbonate, FiO_2_=Fraction of inspired oxygen, SpO_2_=oxygen saturation, Pplat=pleateau pressure, Pmean=mean airway pressure, Ppeak=peak airway pressure.

|  | MIMIC-IV (n=3,706) | Amsterdamumcdb (n=2,768) | Erasmus MC (n=803) | Overall (n=7,277) |
| --- | --- | --- | --- | --- |
| **Gas exchange parameters** |  |  |  |  |
| PaO_2_ | 2.5 | 6.6 | 8 | 4.6 |
| PaCO_2_ | 2.5 | 6.7 | 8.2 | 4.6 |
| pH | 2.5 | 6.6 | - | 3.7 |
| Base excess | 2.5 | 6.6 | 8 | 4.6 |
| Lactic acid | 1.7 | 1.9 | 8 | 2.5 |
| HCO_3_^–^ | 2 | 6.6 | - | 3.4 |
| FiO_2_ | 5.9 | 463 | 11.8 | 171.4 |
| SpO_2_ | 25.9 | 309.8 | 14.4 | 127.1 |
| **Ventilatory parameters** |  |  |  |  |
| Pplat | 2.1 | - | 2.2 | 1.4 |
| Pmean | 4.8 | 231.1 | 11 | 87.1 |
| Ppeak | 1.8 | 231.3 | 9.4 | 85.4 |
| PEEP | 30.8 | 231 | 14.2 | 101.2 |
| Respiratory rate | 4.9 | 231.1 | - | 86 |
| Minute volume | 5.1 | 231.1 | 18.4 | 88.1 |
| **Inflammatory markers** |  |  |  |  |
| White blood cell count | 1.7 | 1.5 | - | 1.4 |
| **Other parameters** |  |  |  |  |
| Heart rate | 26.5 | 311.3 | - | 126.3 |
| Temperature | 7.6 | 42.9 | 7.6 | 20.3 |
| Diastolic blood pressure | 15.6 | 296.1 | - | 115.1 |
| Systolic blood pressure | 15.6 | 296.2 | - | 115.1 |
| Mean arterial pressure | 15.7 | 296.2 | - | 115.2 |
| Ventilator mode | 5.1 | 231.2 | 40.5 | 90.6 |

Figure E3: Histograms describing the distributions of number of logged ventilator modes per day, (a) in the AmsterdamUMCdb database, and (b) for the different databases, not showing the patients with more than 100 logged ventilator modes per day (566 in the AmsterdamUMCdb and 45 in the EMC database).

(a)


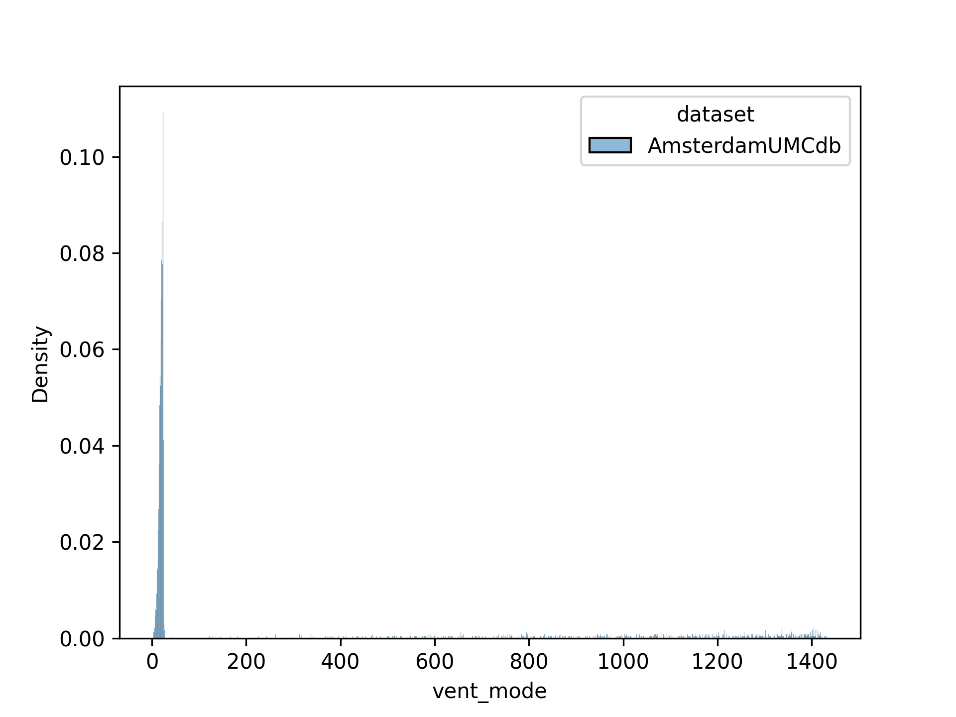


(b)


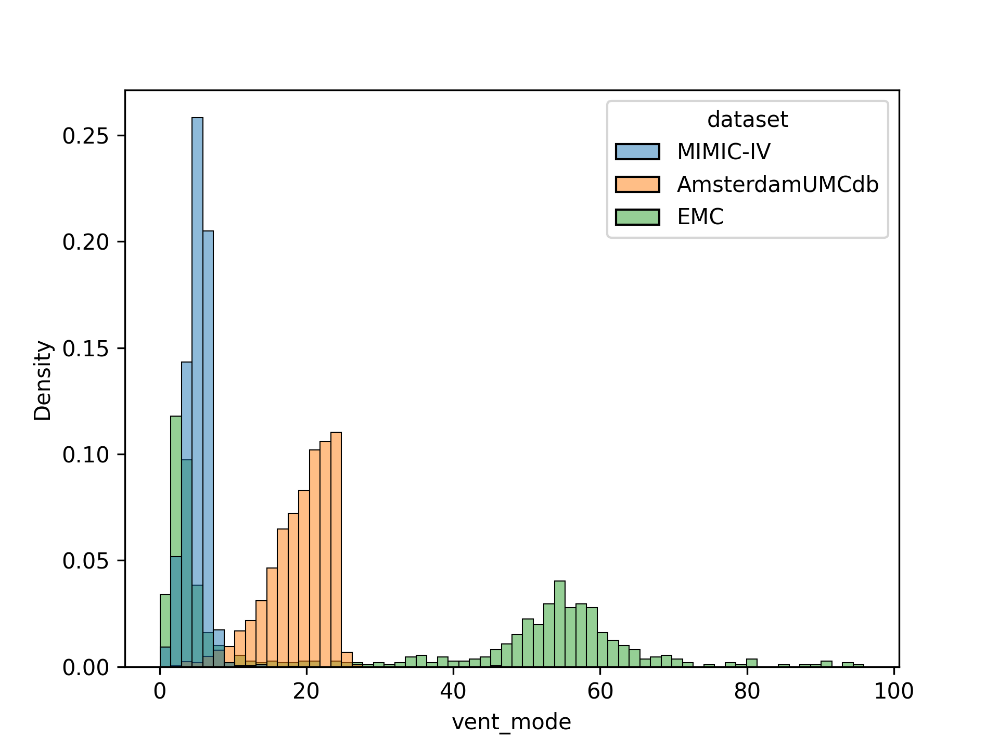


**Appendix B:** STROBE Statement—Checklist of items that should be included in reports of cohort studies.

|  | Item No | Recommendation | Page No |
| --- | --- | --- | --- |
| **Title and abstract** | 1 | (*a*) Indicate the study’s design with a commonly used term in the title or the abstract |  |
|  |  | (*b*) Provide in the abstract an informative and balanced summary of what was done and what was found | 0-2 |
| Introduction | | | |
| Background/rationale | 2 | Explain the scientific background and rationale for the investigation being reported | 3 |
| Objectives | 3 | State specific objectives, including any prespecified hypotheses | 3-4 |
| Methods | | | |
| Study design | 4 | Present key elements of study design early in the paper | 5 |
| Setting | 5 | Describe the setting, locations, and relevant dates, including periods of recruitment, exposure, follow-up, and data collection | 5 |
| Participants | 6 | (*a*) Give the eligibility criteria, and the sources and methods of selection of participants. Describe methods of follow-up | 5-6 |
|  |  | (*b*) For matched studies, give matching criteria and number of exposed and unexposed |  |
| Variables | 7 | Clearly define all outcomes, exposures, predictors, potential confounders, and effect modifiers. Give diagnostic criteria, if applicable | 6-7 |
| Data sources/ measurement | 8* | For each variable of interest, give sources of data and details of methods of assessment (measurement). Describe comparability of assessment methods if there is more than one group | 7 |
| Bias | 9 | Describe any efforts to address potential sources of bias | 9 |
| Study size | 10 | Explain how the study size was arrived at | - |
| Quantitative variables | 11 | Explain how quantitative variables were handled in the analyses. If applicable, describe which groupings were chosen and why | 7-10 |
| Statistical methods | 12 | (*a*) Describe all statistical methods, including those used to control for confounding | 7-8 |
|  |  | (*b*) Describe any methods used to examine subgroups and interactions |  |
|  |  | (*c*) Explain how missing data were addressed |  |
|  |  | (*d*) If applicable, explain how loss to follow-up was addressed |  |
|  |  | (*e*) Describe any sensitivity analyses |  |
| Results | | |  |
| Participants | 13* | (a) Report numbers of individuals at each stage of study—eg numbers potentially eligible, examined for eligibility, confirmed eligible, included in the study, completing follow-up, and analysed | 12 |
|  |  | (b) Give reasons for non-participation at each stage |  |
|  |  | (c) Consider use of a flow diagram |  |
| Descriptive data | 14* | (a) Give characteristics of study participants (eg demographic, clinical, social) and information on exposures and potential confounders | 12 |
|  |  | (b) Indicate number of participants with missing data for each variable of interest |  |
|  |  | (c) Summarise follow-up time (eg, average and total amount) |  |
| Outcome data | 15* | Report numbers of outcome events or summary measures over time | 12 |

| Main results | 16 | (*a*) Give unadjusted estimates and, if applicable, confounder-adjusted estimates and their precision (eg, 95% confidence interval). Make clear which confounders were adjusted for and why they were included | 12-15 |
| --- | --- | --- | --- |
|  |  | (*b*) Report category boundaries when continuous variables were categorized |  |
|  |  | (*c*) If relevant, consider translating estimates of relative risk into absolute risk for a meaningful time period |  |
| Other analyses | 17 | Report other analyses done—eg analyses of subgroups and interactions, and sensitivity analyses | 15 |
| Discussion | | | |
| Key results | 18 | Summarise key results with reference to study objectives | 16 |
| Limitations | 19 | Discuss limitations of the study, taking into account sources of potential bias or imprecision. Discuss both direction and magnitude of any potential bias | 19-20 |
| Interpretation | 20 | Give a cautious overall interpretation of results considering objectives, limitations, multiplicity of analyses, results from similar studies, and other relevant evidence | 16-18 |
| Generalisability | 21 | Discuss the generalisability (external validity) of the study results | 17-18 |
| Other information | | | |
| Funding | 22 | Give the source of funding and the role of the funders for the present study and, if applicable, for the original study on which the present article is based | - |

*Give information separately for exposed and unexposed groups.

**Appendix C:** Pre-processing of ventilator modes

We pre-processed 'raw' ventilation modes, as logged by various ventilators, in four steps (Figure E4):

- In **step 1**, we categorized each raw mode into a ‘controlled’, ‘combined’, ‘assisted’, ‘CPAP’ or ‘non-invasive/no ventilation’ mode (ie, any mode logged for patients not intubated), using remapping lists drafted by clinical experts (see Table E4).
- The prevalence of assist-control (ie, ‘combined’) modes complicate the defining of switch attempts, requiring a unified approach for studying them. Therefore, in **step 2**, we remapped each combined mode to either a controlled or assisted mode, based on the nearest measured spontaneous respiratory rate (RR), considering only spontaneous RRs which were measured within one hour from the logged combined mode. We remapped the combined mode to an assisted mode if the spontaneous RR was 10 breaths/min or higher, and to a controlled mode otherwise. In absence of a spontaneous RR measurement within one hour, we compared the nearest *set* and *observed* RRs (if these were both measured within one hour). We remapped the combined mode to an assisted mode if the observed RR was 1 or more breaths/min higher than the set RR, and to a controlled mode otherwise. In the absence of both spontaneous RR and a set/observed RR combination, we remapped the combined mode to a controlled mode.
- In **step 3**, we remapped CPAP modes to an assisted mode if it was logged during invasive ventilation (based on the logged in- and extubation times), and to a non-invasive/no ventilation mode otherwise.
- In **step** 4, to minimize abrupt ventilator mode transitions, eg, due to clinical interventions requiring temporary resumption of controlled ventilation, we decided to consider mode transitions only if a patient remained in the new mode for at least one hour. Hence, if another ventilator mode is logged within 1 hour from moment of transitioning, the ventilator mode was replaced by the mode which was logged before the transition.

Figure E5a shows an example of a patient where combined modes are remapped to controlled modes, based on the nearest spontaneous RR measurements which are under 10 breaths/min. Figure E5b shows an example of a patient where combined modes are remapped to assisted modes, whereas no spontaneous RR measurements were available near these modes. The difference between the nearest set and observed RR measurements are bigger than 1 breaths/min. Figure E5c shows an example of a patient where a logged assisted mode is remapped to a controlled mode, as it represents an abrupt transition which lasts for shorter than one hour.

Figure E4: Schematic visualization of the different steps of the pre-processing of the logged ventilator modes.


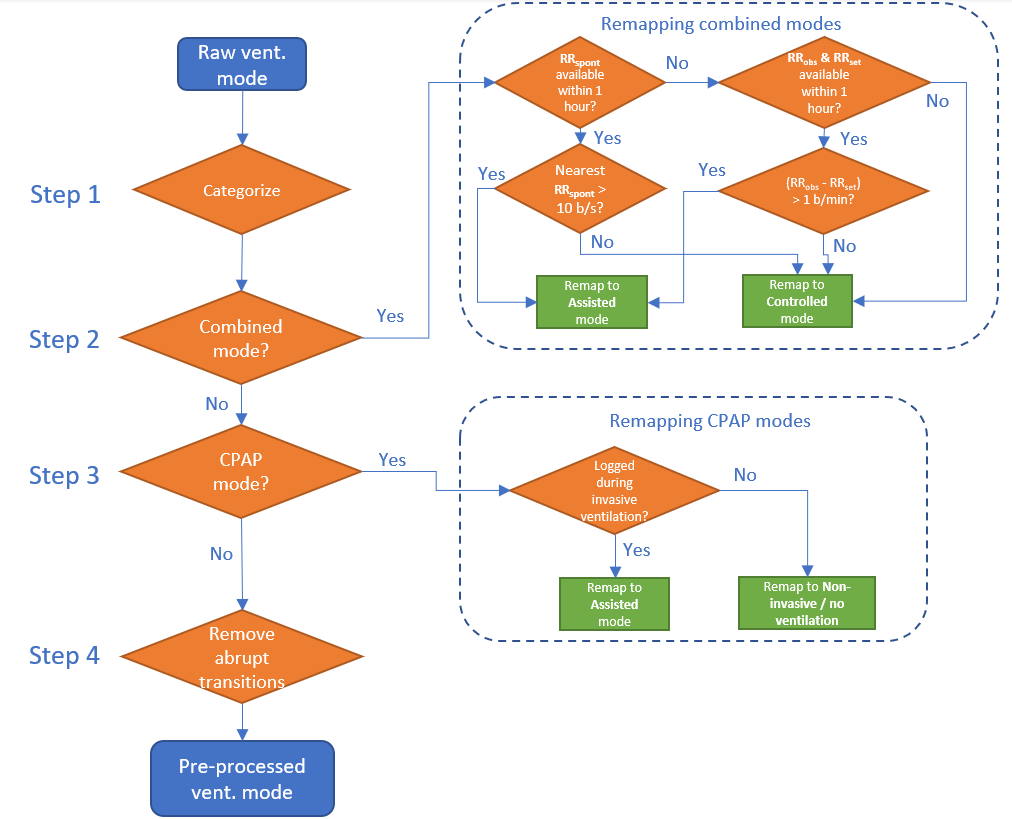


Figure E5: Examples of the pre-processing of logged ventilator modes of (a) a patient whose combined modes were re-mapped to controlled modes based on spontaneous respiratory rate, (b) a patient whose combined modes were re-mapped to assisted modes based on the difference between set and observed respiratory rate and (c) a patient whose abrupt ventilator mode transition was filtered.
The yellow regions highlight the ventilator modes which are remapped. The red star represents the moment of a switch attempt.

(a)


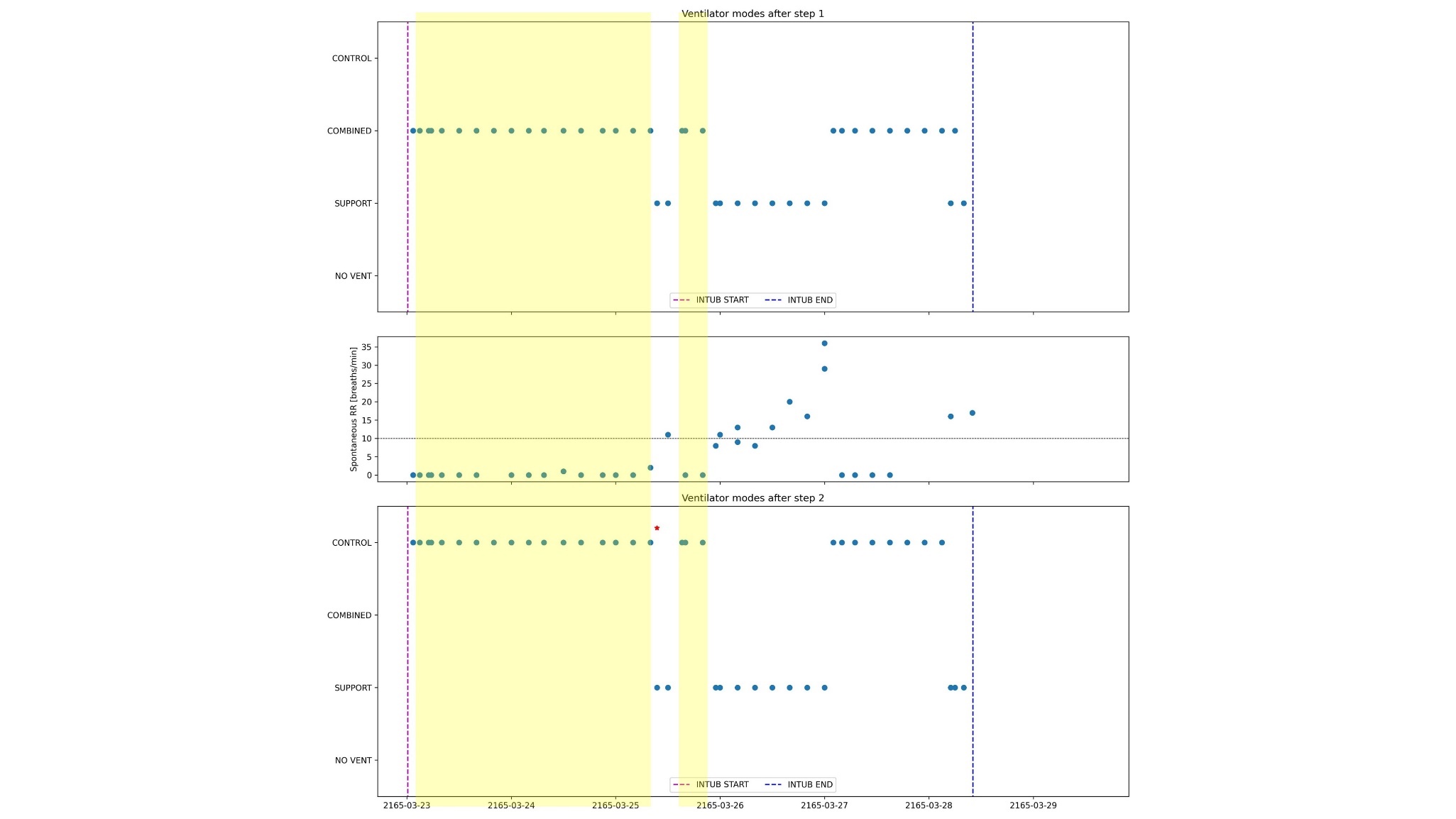


(b)


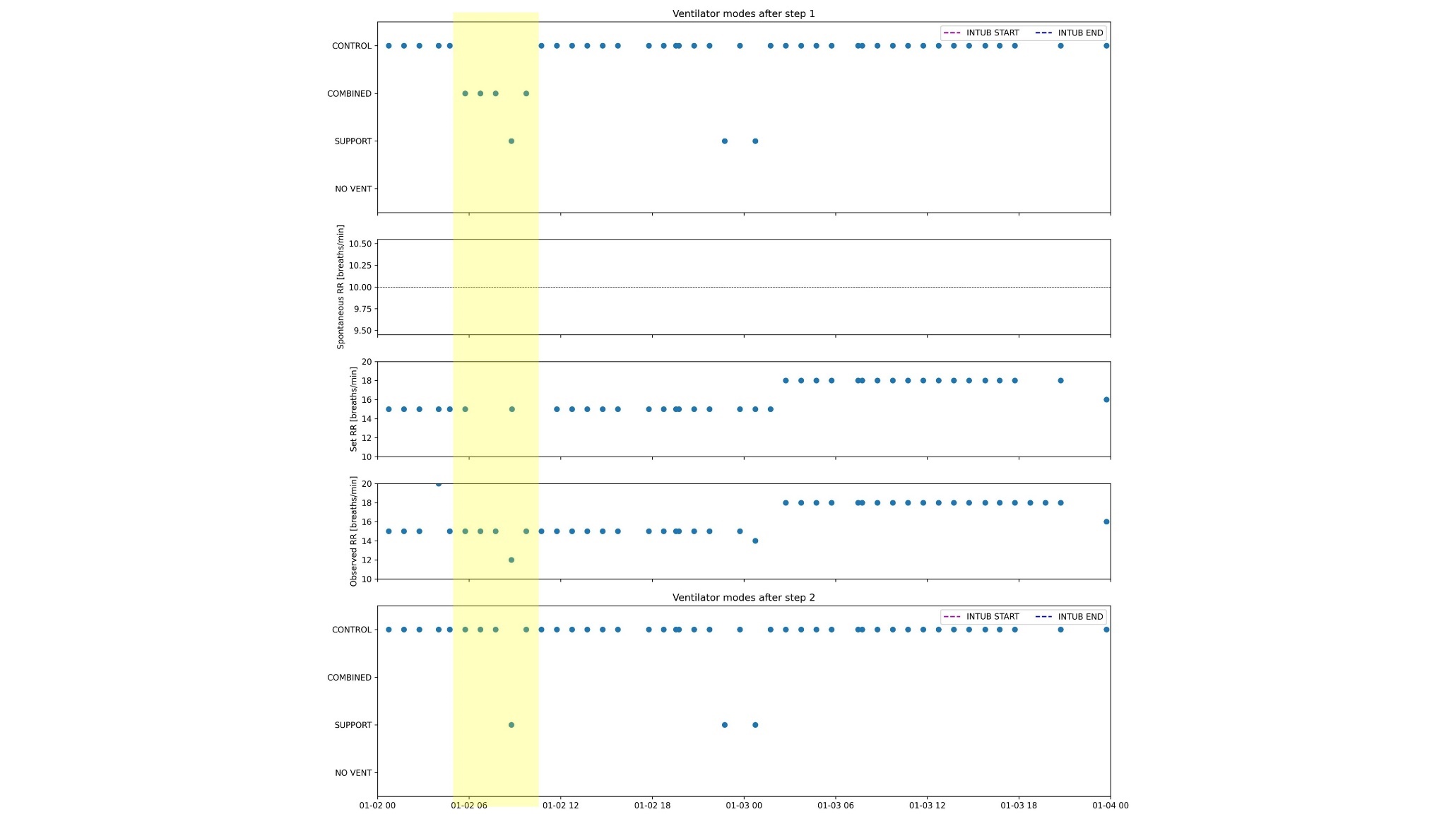


(c)


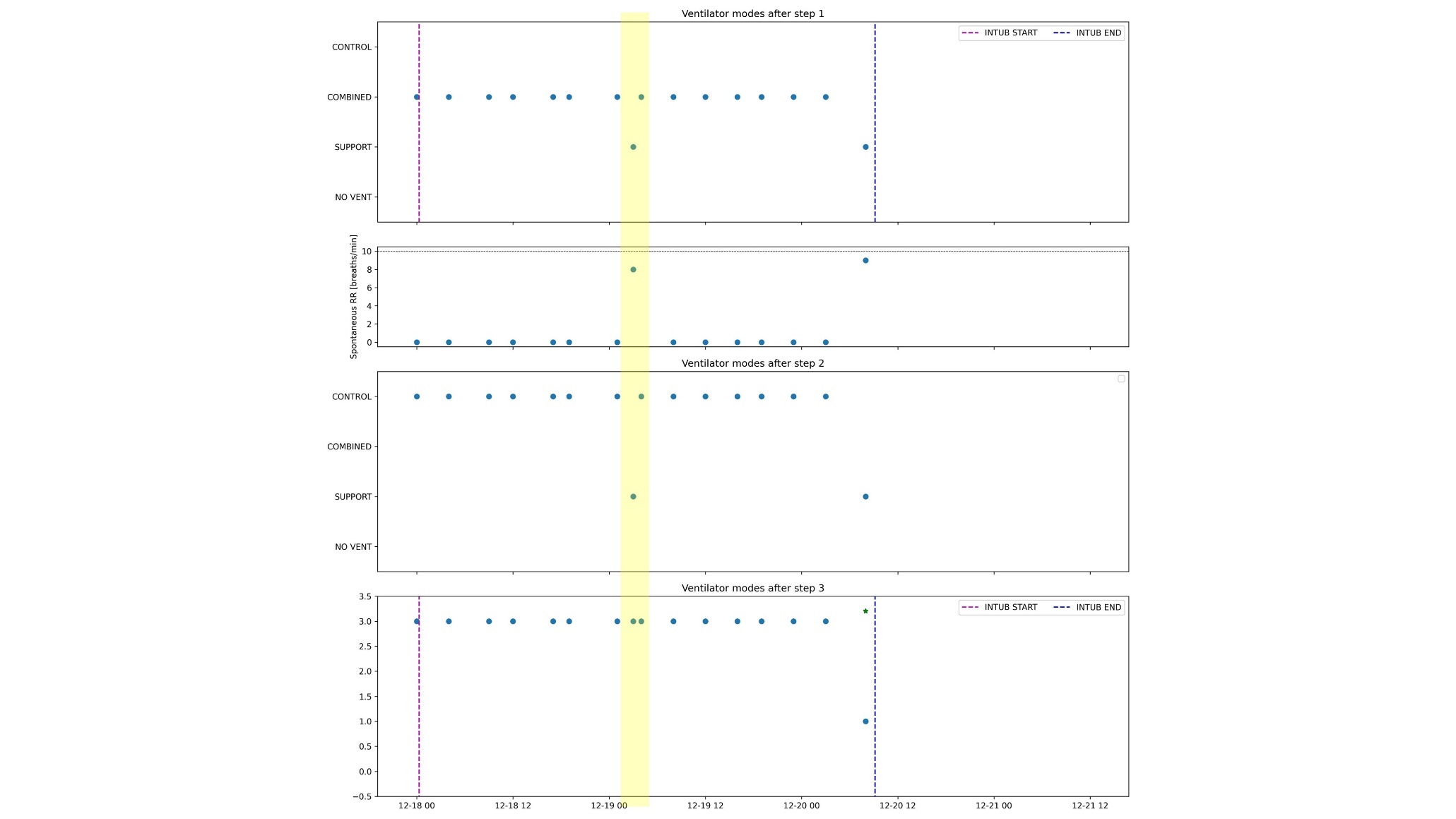


Table E4: Lists of modes logged by mechanical ventilators, mapped to either controlled, assisted, combined, CPAP or non-invasive/no ventilation mode.

| Controlled mode | Assisted mode | Combined mode | CPAP | Non-invasive mode or no ventilation |
| --- | --- | --- | --- | --- |
| PC | PS/CPAP | SIMV(VC)+PS | CPAP | Ambient |
| VC | NAVA | SIMV/AutoFlow | CPAP/PPS | Apnea Ventilation |
| PRVC | VS | SIMV(PC)+PS | CPAP/PSV | DuoPaP |
| VC (No trig) | PS/CPAP (trig) | PC/PS(AUTO,PT) | CPAP/PSV+Apn TCPL | nCPAP-PS |
| PRVC (No trig) | SPONT | APRV/Biphasic+ApnVol | CPAP/PSV+ApnPres | NIV |
| PC (No trig) | PSV/SBT | MMV | CPAP/PSV+ApnVol | NIV-ST |
| PCV+ | spn-cpap | CMV/ASSIST/AutoFlow |  | Standby |
| P-CMV | SPN-CPAP/+PS | CMV/ASSIST |  | SYNCHRON MASTER |
| CMV/AutoFlow | SPN-CPAP+PS+ATC | ASV |  | SYNCHRON SLAVE |
| CMV | Mode SPN-CPAP | APV (simv) |  | HIGH FLOW |
| (S) CMV | Mode SPN-CPAP//PS | APV (cmv) |  | HFOT |
| IPPV/+AF | Mode MAN/SPONT | APRV |  | HFT |
| Mode IPPV | Mode SPN-CPAP/+PS/ATC | APRV/Biphasic+ApnPress |  | NIV PS/CPAP |
| Mode VC-CMV/+AF/ATC | Mode CPAP/ASB | MMV/PSV |  | optiflow |
| Mode VC-CMV | Mode SPN-CPAP/Mode APNEA VENTILATION/+PS | pc-bipap |  | PS/CPAP(NIV) |
| IPPV/AutoFlow | PS(AUTO) | Bivent/APRV |  | Optiflow |
| PCV-VG | PS | PC-APRV+AutoRelease |  | DuoPaP |
| Mode PC-CMV | SPN-CPAP | Mode VC-AC/+AF/ATC |  | nCPAP-PS |
| Pressure mode | Mode CPAP | MMV/AutoFlow |  | NIV |
| Mode VC-CMV/+AF | CPAP,CPAP/PSV | MMV/PSV/AutoFlow |  | NIV-ST |
| Mode VC-CMV//AF | CPAP/PSV | PC-Bipap |  | PC in NIV |
| Volume mode | PSV | P-SIMV |  | PS/CPAP in NIV |
| PCV | CPAP/ASB | PCV+/PSV |  |  |
| Mode IPPV/AutoFlow | SPN-CPAP+PS | PCV+Assist |  |  |
| PRVC | Mode SPN-CPAP/+PS | PRES/AC |  |  |
|  |  | PRVC/AC |  |  |
|  |  | PRVC/SIMV |  |  |
|  |  | SIMV |  |  |
|  |  | SIMV/PRES |  |  |
|  |  | SIMV/PSV |  |  |
|  |  | SIMV/PSV/AutoFlow |  |  |

**Appendix D:** Predictive analysis

Methods

To investigate the potential to predict switch failure both before and shortly after a switch attempt, we trained machine learning models using LASSO regression. These models were designed to predict switch failure either before the attempt (model 1) or three hours after the attempt (model 2), as detailed in Figure 1d of the main text. Model 1 included all patients who underwent a switch attempt (n=6,715), while model 2 focused on patients who attempted a switch and remained in assisted mode for at least three hours (n=5,620). For all patients, we used variables collected prior to the switch attempt, derived from the switch analysis (see main text), as input features. Additionally, for the patients included in model 2, we incorporated Δ_3h_ values obtained from follow-up analysis as input features (see Figure 1d in the main text). Each patient was labelled as either ‘failed’ (encoded as 1) or ‘successful’ (encoded as 0). We performed cross-validation to evaluate the discriminative performance. Furthermore, to gain explainability of the trained models, we examined how much different variable groups contributed to this prediction.

This analysis consisted of 8 steps:

1. **Variable selection:** A priori, we selected a set of variables which were available for at least two thirds of the included patients.
2. **Data split**: We split the patients into a train (95%) and validation cohort (5%).
3. **Missing data imputation**: We trained a K-Nearest-Neighbour (KNN) imputation algorithm using the train cohort, and used it to fill in missing values in both the train and validation cohorts. This algorithm imputes missing values using values from the five nearest neighbours (i.e., the shortest Euclidean distance regarding the remaining variables) that have a value for that variable, averaging these uniformly.
4. **Data normalization**: We normalized the imputed train and test cohorts by centering and scaling each variable based on its standard deviation, ensuring that all variables in the training data are zero-mean and have unit variance before these are used for model training.
5. **Lasso strength optimization**: We optimized the LASSO penalization strength (λ) through a grid search, searching in an evenly spaced log range from 10^-4^ to 10^4^, using the train cohort only. For each candidate λ, a nested, 5-fold cross-validation was performed using the train cohort, meaning that the train cohort is again split up in two datasets: the ‘inner train’ and ‘inner validation’ cohort, comprising 80% and 20% of the train cohort, respectively. The model is trained using the candidate λ in the inner train cohort, and evaluated in the inner validation cohort. Here, we only evaluated the model’s discriminative performance in terms of area under the ROC curve (AUC). This is repeated five times, ensuring that each patient in the train cohort is in the inner validation cohort once. The λ that yields the highest cross-validated mean AUC is selected.
6. **Model training, prediction and evaluation**: Using the optimized λ from the previous step, we train a model in the train cohort, and use it to predict switch failure in the validation cohort. We evaluated these predictions in terms of AUC.
7. **Cross-validation**: Steps 2-6 are repeated 10 times, ensuring that each patient is in the validation cohort once (ie, 10-fold cross-validation), resulting in 10 AUCs. Then we reported the mean AUC, and the interquartile range (IQR).
8. **Variable group removal**: The whole process (ie, steps 1-7) is repeated 4 times, each time removing a set of variables (Table E5) after step 1, to quantify the difference in discriminative performance as a result of removing these features.

Results:

Both models yielded limited discriminative performance, with a cross-validated mean AUC of 0.59 (IQR: 0.57 – 0.61) and 0.61 (IQR: 0.59 – 0.62) for model 1 and model 2, respectively. In both models, removal of the gas exchange parameters at the moment of a switch attempt resulted in the biggest drop in discriminative performance (3 percentage points), followed by the ventilatory parameters at the moment of the switch attempt and the Δ_3h_ values of the gas exchange parameters (1 percentage point). Removal of the Δ_3h_ values for the ventilatory parameters in model 2 did not cause a drop in performance (Table E5).

Table E5: Changes in predictive performance for various left-out variables sets, for the two models. PaO_2_=arterial oxygen pressure, PaCO_2_=Partial pressure of carbon dioxide, PEEP= Positive end-expiratory pressure, HCO_3_^-^ = bicarbonate, FiO_2_=Fraction of inspired oxygen, SpO_2_=oxygen saturation, Pplat=pleateau pressure, ΔP=driving pressure, Pmean=mean airway pressure, Ppeak=peak airway pressure, C_RS_=respiratory system compliance.

| Left-out variables | Model 1 | | Model 2 | |
| --- | --- | --- | --- | --- |
|  | **Mean  AUC (IQR)** | **Drop in mean AUC** | **Mean  AUC (IQR)** | **Drop in mean AUC** |
| None  (ie, using all variables) | 0.59 (0.57 – 0.61) | - | 0.61 (0.59 – 0.62) | - |
| Gas exchange parameters: PaO_2_, PaCO_2_, PF-ratio, pH, Base excess, Lactic acid, HCO_3_^–^, FiO_2_, SpO_2_ | 0.56 (0.54 – 0.57) | 0.03 | 0.58 (0.56 – 0.59) | 0.03 |
| Ventilatory parameters: Pplat, ΔP, Pmean, Ppeak, PEEP, RR, minute volume, C_RS_ | 0.58 (0.58 – 0.59) | 0.01 | 0.60 (0.58 – 0.61) | 0.01 |
| Δ_3h_ values of gas exchange parameters: PaO_2_, PaCO_2_, PF-ratio, pH, base excess, FiO_2_, SpO_2_ | - | - | 0.61 (0.59 – 0.62) | 0.00 |
| Δ_3h_ values of ventilatory parameters: Ppeak, PEEP, repiratory rate, minute volume | - | - | 0.61 (0.59 – 0.63) | 0.00 |

**Appendix E:** Sensitivity analyses

Methods:
*Influence of mortality in switch failure definition*
As mortality is included in the definition of a failed switch attempt (see Section 2.2), this may have influenced our findings on mortality endpoints. To address this, we repeated the baseline analysis (which included findings on mortality outcomes), only including patients who survived at least 72 hours after the first switch attempt.

*Consistency of findings across individual datasets*
To examine the generalisability of findings across datasets (also taking into consideration the observed differences in logging frequency of the ventilator modes, and therefore varying ‘resolution’ with which the switches could be analysed between the included datasets; see appendix A), we compared the findings of the baseline, before switch, and after switch analyses across the three included datasets.

*Comparison of two ‘types’ of switch attempts*
Due to our pre-processing of combined modes, the observed switch attempts were either an actual switch in ventilatory mode from controlled to assisted ventilation, or a change in respiratory rate during a combined mode. Therefore, we also compared the findings of the baseline, before switch, and after switch analyses between these two types of switch attempts.

*‘Early’ vs ‘late’ switch failures*
We repeated the baseline, before switch, and after switch analyses, marking the switch attempts that failed *within* eight hours as ‘early’, and after eight hours as ‘late’ failures. This cut-off was chosen based on the distribution of the times between switch attempt and failure among the patients with a failed switch attempt (see Figure 2, main text).

*After switch analysis with varying follow-up times*
Fifth, we repeated the after switch analysis at different follow-up times up to eight hours, collecting changes in time-varying variables (ie, ‘Δs’) for up to eight hours after the switch attempt. For each follow-up hour, we sampled the most recent time-varying variables that were available x hours after the switch attempt, and calculated the Δs by subtracting it from the value sampled at the corresponding switch attempt. Again, the Δ value was considered missing if the variable was missing at the moment of the switch attempt, or there was no new measurement within x hours after the switch attempt (or both).

*Sensitivity of predictive analysis*Sixth, we explored the robustness of the predictive analysis by (1) evaluating the added value of a flexible, non-linear Light Gradient Boosting Machine model (LightGBM), (2) testing sensitivity to the imputation method using scikit-learn’s IterativeImputer, and (3) restricting the analysis to patients with PaO₂/FiO₂ measurements taken at PEEP levels above 10 cmH₂O.
To examine the added value of using methods capable of handling non-linear relationships, we repeated the predictive analysis using a more flexible model, ie, a Light Gradient Boosting Machine (LightGBM). We repeated steps 1-7 of the predictive analysis (described in Appendix D), but now optimizing the LightGBM hyperparameters ‘boosting_type’ (searched grid: [gbdt, dart]), and ‘max_depth’ (searched grid: [5, 10, unlimited]) in step 5, and training a LightGBM using these optimal hyperparameters in step 6.
We assessed the sensitivity of the predictive analysis to the chosen imputation method by repeating it using an alternative imputation approach—scikit-learn’s 'IterativeImputer'. This imputation method (inspired by R's MICE package[2]) imputes each variable with missing values based on the remaining variables with Bayesian ridge regression in an iterated round-robin fashion. We repeated steps 1-7 of the predictive analysis (described in Appendix D), but now using the alternative imputation method in step 3.

As the PaO₂/FiO₂ showed a stronger association with switch failure when these were taken at PEEP levels above 10 cmH₂O (see main text Table 2), we assessed whether predictions would improve if the predictive analysis is restricted to patients for whom PaO₂/FiO₂ was measured at PEEP > 10 cmH₂O (3,756/6,715 of the patients in the original predictive analysis), first doing this filtering and subsequently repeating steps 1-7 (described in Appendix D).

Results:
*Influence of mortality in switch failure definition*
The baseline analysis including only patients alive 72 after the switch attempt showed very similar results (Supplementary Table E6) compared to the baseline analysis including all included patients (main text table 1), showing similar worse outcomes for among patients with failed switches including higher 28-day mortality (21% vs. 14%), longer median ICU stays (10.8 vs. 8.0 days), extended mechanical ventilation duration (7.5 vs. 4.9 days), and fewer median VFDs (17.9 vs. 22.5 days).

*Consistency of findings across individual datasets*
Results for the different analyses showed to be similar across the three included datasets (Supplementary Tables E7-15), and most of the variables and outcomes significantly associated with treatment failure, and available in all three datasets, showed associations in consistent direction in each dataset (as highlighted using a “‡” in the main text Tables).

*Comparison of two ‘types’ of switch attempts*
5,492/6,715 (82%) of the switch attempts were observed as an actual switch in ventilatory mode from controlled to assisted ventilation and only 18% were observed as a change in respiratory rate within a combined mode. For both ‘types’ of switch attempts, we observed similar associations for most of the variables which showed an overall statistically significantly association with switch failure (Supplementary Tables E16-21). Notably, failed switch attempts observed as a change in respiratory rate within a combined mode, failed earlier compared to the failed switch attempts from controlled to assisted modes (median of 5 vs 9 hours).

*‘Early’ vs ‘late’ switch failures*Results for the sensitivity analysis comparing patients with early (<8 hours) and late switch failures are depicted in Tables E22-24. Results of the baseline analysis were very similar between early and late failures (Table E22). The before switch analysis, however, does show some notable differences (Table E23). The distributions of the gas exchange parameters before the switch were similar, whereas early failures were characterized by higher ventilatory parameters compared to late failures, especially in terms of peak, plateau and driving pressures. In the after switch analysis (Table E24), early failures were characterized by bigger increases in PaCO_2_ and bigger drops in pH three hours after the switch, compared to late failures. Also, the changes that may be interpreted as improvement in ventilatory parameters, especially peak pressure and RR, were less pronounced for early failures as compared to late failures.

*After switch analysis with varying follow-up times*Figure E6 shows the results of the after switch analysis for each time-varying variable separately, comparing different follow-up times up to eight hours. For most variables, obtaining a new value within 1 hour after attempting a switch was uncommon, leading to a high level of missing data among the differences (Δs) at this follow-up time. However, as the follow-up time extends, the likelihood of acquiring a new measurement increases, resulting in a decrease in missing data. In the case of patients with successful switch attempts or late failures, the number of patients with available Δs rises with longer follow-up times. Conversely, among patients with early failed switch attempts, the number of patients unable to provide Δs due to prior failure increases as follow-up time progresses, as evidenced by the 'inverse U shape' of the curves representing the number of patients with available Δs among those experiencing early failures. For most of the variables, the distribution of the changes in variables for successful and failed switch attempts were similar for the different follow-up times. Notable exception are PaCO_2_ and pH: as the between-group differences for the changes in these measurements were fully driven by the early failures, these differences disappear at later follow-up times (Figures E6b and E6d).

*Sensitivity of predictive analysis*Finally, the predictive analysis using the more flexible LightGBM model yielded slightly worse discriminative performance, while the predictive analysis (using LASSO regression) with the alternative imputation method, or restricting it to patients with PaO₂/FiO₂ measurements taken at PEEP levels above 10 cmH₂O, yielded very similar results (Table E25).

Supplementary Tables for sensitivity analyses outline:

- Results of sensitivity analysis comparing findings in patients alive 72 hours after first switch attempt
  - Baseline analysis: **Table E6**
- Results of sensitivity analysis comparing findings across the three individual datasets.
  - Baseline analysis: **Table E7-9**
  - Before switch analysis: **Table E10-12**
  - After switch analysis: **Table E13-15**
- Results of sensitivity analysis comparing findings across switch attempts observed as an actual switch in ventilatory mode from controlled to assisted ventilation, vs changes in respiratory rate during combined modes.
  - Baseline analysis: **Table E16-17**
  - Before switch analysis: **Table E18-19**
  - After switch analysis: **Table E20-21**
- Results of sensitivity analysis comparing findings across “early” and “late” failures among failed switch attempts.
  - Baseline analysis: **Table E22**
  - Before switch analysis: **Table E23**
  - After switch analysis: **Table E24**
- Results of comparing the original predictive analysis with (1) usage of Gradient Boosting Machine model (LightGBM), (2) scikit-learn’s IterativeImputer, and (3) restricting the analysis to patients with PaO₂/FiO₂ measurements taken at PEEP levels above 10 cmH₂O: **Table E25**

Table E6: **Baseline characteristics and endpoints** among patients **still alive 72 hours after the first switch attempt**, grouped by the success, failure or absence of the first switch attempt. Data are in median (IQR) or number (percentage). PaO_2_=arterial oxygen pressure, PaCO_2_=Partial pressure of carbon dioxide, ΔP=driving pressure, C_RS_=respiratory system compliance, MAP=mean arterial pressure, VFDs=ventilator-free days, MV=mechanical ventilation, ICU=intensive care unit.

|  | Successful switch (n=2,127) | Failed switch  (n=4,154) | P value |
| --- | --- | --- | --- |
| **Demographics** |  |  |  |
| Age group, n (%) |  |  |  |
| 18-39 | 176 (8) | 403 (10) | 0.065 |
| 40-49 | 198 (9) | 386 (9) | 1 |
| 50-59 | 348 (16) | 719 (17) | 0.356 |
| 60-69 | 455 (21) | 898 (22) | 0.846 |
| 70-79 | 453 (21) | 829 (20) | 0.221 |
| 80+ | 238 (11) | 504 (12) | 0.283 |
| Female sex (%) | 798(37.5) | 1543(37.1) | 0.956 |
| **Gas exchange** |  |  |  |
| PF-ratio | 215.6 (162.7 - 276.8) | 210.0 (156.1 - 277.5) | 0.406 |
| PaO_2_ (mmHg) | 111.4 (91.6 - 142.0) | 112.0 (91.7 - 143.3) | 0.762 |
| PaCO_2_ (mmHg) | 40.0 (36.5 - 44.3) | 40.6 (36.6 - 45.2) | <0.001 |
| pH | 7.35 (7.31 - 7.4) | 7.34 (7.29 - 7.39) | 0.001 |
| **Respiratory mechanics** |  |  |  |
| ΔP (cmH_2_O) | 11.7 (9.8 - 14.0) | 12.1 (10.0 - 14.6) | <0.001 |
| C_RS_ (mL/cmH_2_O) | 40.6 (32.3 - 50.5) | 38.8 (31.4 - 48.4) | 0.477 |
| **SOFA components** |  |  |  |
| MAP (mmHg) | 73.7 (67.3 - 81.9) | 73.3 (67.0 - 80.5) | 0.072 |
| Bilirubin (µmol/L) | 11.0 (7.0 - 20.5) | 11.5 (6.8 - 21.0) | 0.281 |
| Creatinine (µmol/L) | 97.2 (73.4 - 139.1) | 99.3 (73.7 - 150.3) | 0.209 |
| Platelet count (10^9^/L) | 190.4 (135.0 - 256.0) | 184.1 (127.0 - 248.2) | 0.75 |
| **Baseline severity scores** |  |  |  |
| SAPS-II score | 44.0 (35.0 - 54.0) | 46.0 (37.0 - 57.0) | <0.001 |
| APACHE-II score | 26.0 (21.0 - 31.5) | 25.0 (19.0 - 31.0) | 0.231 |
| **Secondary Endpoints** |  |  |  |
| 28-d mortality (%) | 303 (14) | 889 (21) | <0.001 |
| VFDs-28 (days) | 22.5 (14.2 - 25.2) | 17.9 (0.7 - 23.2) | <0.001 |
| Length of MV (days) | 4.9 (2.7 - 9.1) | 7.5 (4.3 - 13.0) | <0.001 |
| Length of ICU stay (days) | 8.0 (5.0 - 13.8) | 10.8 (6.7 - 18.2) | <0.001 |
| **Switch characteristics** |  |  |  |
| Time between ICU admission and switch attempt (days) | 1.8 (0.8 - 2.9) | 1.2 (0.5 - 2.5) | <0.001 |
| Time between switch attempt and switch failure (hours) | - | 8.0 (4.0 - 19.3) | - |
| Number of secondary switch attempts (n) | - | 2.0 (1.0 - 4.0) | - |

Table E7: **Baseline characteristics and endpoints** of the **MIMIC-IV cohort**, grouped by the success, failure or absence of the first switch attempt. Data are in median (IQR) or number (percentage). *p-values are given for the comparison of distributions with successful and failed switch attempts. PaO_2_=arterial oxygen pressure, PaCO_2_=Partial pressure of carbon dioxide, ΔP=driving pressure, C_RS_=respiratory system compliance, MAP=mean arterial pressure, VFDs=ventilator-free days, MV=mechanical ventilation, ICU=intensive care unit.

|  | Successful switch (n=962) | Failed switch (n=2,452) | No switch  (n=292) | P value* |
| --- | --- | --- | --- | --- |
| **Demographics** |  |  |  |  |
| Age group, n (%) |  |  |  |  |
| 18-39 | 76 (8) | 220 (9) | 35 (12) | 0.344 |
| 40-49 | 89 (9) | 234 (10) | 37 (13) | 0.845 |
| 50-59 | 186 (19) | 467 (19) | 45 (15) | 0.847 |
| 60-69 | 233 (24) | 611 (25) | 67 (23) | 0.692 |
| 70-79 | 238 (25) | 524 (21) | 62 (21) | 0.036 |
| 80+ | 140 (15) | 396 (16) | 46 (16) | 0.272 |
| Female sex (%) | 426 (44.3) | 982 (40.0) | 110 (37.7) | 0.025 |
| **Gas exchange** |  |  |  |  |
| PF-ratio | 217.9 (161.3 - 280.5) | 201.3 (144.6 - 273.4) | 184.7 (123.9 - 265.5) | 0.011 |
| PaO_2_ (mmHg) | 128.2 (99.8 - 169.1) | 119.1 (94.8 - 157.1) | 113.0 (88.1 - 149.4) | <0.001 |
| PaCO_2_ (mmHg) | 39.9 (36.2 - 44.6) | 40.8 (36.0 - 46.3) | 39.5 (35.0 - 46.0) | 0.003 |
| pH | 7.36 (7.32 - 7.4) | 7.34 (7.29 - 7.4) | 7.31 (7.25 - 7.37) | <0.001 |
| **Respiratory mechanics** |  |  |  |  |
| ΔP (cmH_2_O) | 11.7 (9.8 - 14.0) | 12.2 (10.1 - 14.6) | 13.0 (10.8 - 15.0) | <0.001 |
| C_RS_ (mL/cmH_2_O) | 40.4 (32.3 - 50.1) | 38.6 (31.2 - 48.0) | 36.2 (29.8 - 45.4) | 0.438 |
| **SOFA components** |  |  |  |  |
| MAP (mmHg) | 73.9 (67.3 - 81.9) | 73.1 (66.9 - 80.5) | 71.1 (65.0 - 80.7) | 0.037 |
| Bilirubin (µmol/L) | 13.7 (6.8 - 27.4) | 12.8 (6.8 - 25.6) | 14.5 (7.7 - 31.6) | 0.794 |
| Creatinine (µmol/L) | 97.2 (70.7 - 150.3) | 106.1 (71.8 - 170.2) | 154.7 (92.4 - 218.1) | 0.066 |
| Platelet count (10^9^/L) | 179.0 (125.0 - 247.2) | 179.0 (122.0 - 246.0) | 157.0 (106.0 - 213.9) | 0.941 |
| **Baseline severity scores** |  |  |  |  |
| SAPS-II score | 44.0 (34.5 - 54.0) | 46.0 (38.0 - 57.0) | 53.5 (43.2 - 66.8) | <0.001 |
| **Secondary Endpoints** |  |  |  |  |
| 28-d mortality (%) | 124 (12) | 683 (27) | 255 (87) | <0.001 |
| VFDs-28 (days) | 24.1 (19.0 - 25.4) | 17.4 (0.0 - 23.1) | 0.0 (0.0 - 0.0) | <0.001 |
| Length of MV (days) | 3.8 (2.5 - 6.8) | 6.6 (3.8 - 11.7) | 3.4 (2.3 - 4.7) | <0.001 |
| Length of ICU stay (days) | 6.9 (4.7 - 10.9) | 9.3 (5.9 - 15.2) | 3.8 (2.8 - 5.4) | <0.001 |
| **Switch characteristics** |  |  |  |  |
| Time between ICU admission and switch attempt (days) | 1.7 (0.8 - 2.6) | 1.2 (0.5 - 2.6) | - | 0.356 |
| Time between switch attempt and switch failure (hours) | - | 8.0 (4.0 - 18.0) | - | - |
| Number of secondary switch attempts (n) | - | 2 (1 - 4) | - | - |

Table E8: **Baseline characteristics and endpoints** of the **AmsterdamUMCdb** cohort, grouped by the success, failure or absence of the first switch attempt. Data are in median (IQR) or number (percentage). *p-values are given for the comparison of distributions with successful and failed switch attempts. PaO_2_=arterial oxygen pressure, PaCO_2_=Partial pressure of carbon dioxide, VFDs=ventilator-free days, MV=mechanical ventilation, ICU=intensive care unit.

|  | Successful switch (n=959) | Failed switch (n=1,617) | No switch  (n=192) | P value* |
| --- | --- | --- | --- | --- |
| **Demographics** |  |  |  |  |
| Age group, n (%) |  |  |  |  |
| 18-39 | 101 (11) | 199 (12) | 20 (10) | 0.155 |
| 40-49 | 113 (12) | 179 (11) | 20 (10) | 0.603 |
| 50-59 | 171 (18) | 317 (20) | 29 (15) | 0.399 |
| 60-69 | 229 (24) | 368 (23) | 41 (21) | 0.46 |
| 70-79 | 231 (24) | 391 (24) | 58 (30) | 0.961 |
| 80+ | 114 (12) | 163 (10) | 24 (12) | 0.285 |
| Female sex (%) | 301 (31.4) | 572 (35.4) | 68 (35.4) | 0.018 |
| **Gas exchange** |  |  |  |  |
| PF-ratio | 211.0 (162.7 - 275.4) | 216.2 (164.4 - 281.7) | 198.0 (146.0 - 261.0) | 0.059 |
| PaO_2_ (mmHg) | 105.8 (89.3 - 127.8) | 109.6 (90.8 - 134.6) | 113.0 (90.6 - 136.3) | <0.001 |
| PaCO_2_ (mmHg) | 39.8 (36.6 - 43.6) | 39.9 (36.6 - 43.8) | 40.6 (36.0 - 45.7) | 0.566 |
| pH | 7.34 (7.3 - 7.39) | 7.34 (7.3 - 7.39) | 7.29 (7.23 - 7.35) | 0.811 |
| **SOFA components** |  |  |  |  |
| Bilirubin (µmol/L) | 10.0 (6.8 - 16.0) | 10.0 (6.7 - 16.6) | 11.0 (7.0 - 22.0) | 0.306 |
| Creatinine (µmol/L) | 95.2 (73.9 - 130.2) | 95.5 (74.8 - 130.0) | 111.4 (80.2 - 150.8) | 0.653 |
| Platelet count (10^9^/L) | 197.2 (149.5 - 256.4) | 189.1 (131.5 - 249.4) | 190.2 (118.3 - 244.6) | 0.009 |
| **Baseline severity scores** |  |  |  |  |
| APACHE-II score | 26.0 (21.0 - 32.0) | 26.0 (20.0 - 32.0) | 33.0 (26.0 - 39.0) | 0.452 |
| **Secondary Endpoints** |  |  |  |  |
| 28-d mortality (%) | 195 (20) | 455 (28) | 173 (90) | <0.001 |
| VFDs-28 (days) | 20.4 (5.7 - 24.4) | 14.2 (0.0 - 21.7) | 0.0 (0.0 - 0.0) | <0.001 |
| Length of MV (days) | 5.9 (3.0 - 10.8) | 7.9 (4.3 - 14.6) | 2.9 (2.2 - 4.2) | <0.001 |
| Length of ICU stay (days) | 8.1 (4.8 - 16.6) | 11.0 (6.0 - 20.5) | 3.1 (2.6 - 4.5) | <0.001 |
| **Switch characteristics** |  |  |  |  |
| Time between ICU admission and switch attempt (days) | 1.7 (0.6 - 2.7) | 1.3 (0.4 - 2.3) | - | 0.004 |
| Time between switch attempt and switch failure (hours) | - | 7.7 (3.0 - 20.0) | - | - |
| Number of secondary switch attempts (n) | - | 2.0 (1.0 - 3.0) | - | - |

Table E9: **Baseline characteristics and endpoints** of the **Erasmus Medical Center** cohort, grouped by the success, failure or absence of the first switch attempt. Data are in median (IQR) or number (percentage). *p-values are given for the comparison of distributions with successful and failed switch attempts. PaO_2_=arterial oxygen pressure, PaCO_2_=Partial pressure of carbon dioxide, VFDs=ventilator-free days, MV=mechanical ventilation, ICU=intensive care unit.

|  | Successful switch (n=270) | Failed switch (n=455) | No switch  (n=78) | P value* |
| --- | --- | --- | --- | --- |
| **Demographics** |  |  |  |  |
| Female sex (%) | 94 (34.8) | 127 (27.9) | 28(35.9) | 0.055 |
| **Gas exchange** |  |  |  |  |
| PF-ratio | 219.3 (168.6 - 269.6) | 224.0 (165.2 - 282.1) | 222.0 (145.4 - 309.0) | 0.597 |
| PaO_2_ (mmHg) | 98.5 (85.5 - 110.5) | 97.0 (86.2 - 110.8) | 100.2 (86.8 - 127.6) | 0.753 |
| PaCO_2_ (mmHg) | 41.3 (37.5 - 44.9) | 41.6 (37.7 - 45.1) | 41.6 (36.9 - 45.8) | 0.514 |
| **Respiratory mechanics** |  |  |  |  |
| ΔP (cmH_2_O) | 11.8 (9.4 - 14.1) | 11.8 (9.5 - 14.2) | 12.0 (9.7 - 15.1) | 0.883 |
| C_RS_ (mL/cmH_2_O) | 42.1 (32.5 - 57.0) | 40.2 (32.4 - 53.4) | 40.4 (29.9 - 52.2) | 0.51 |
| **SOFA components** |  |  |  |  |
| Platelet count (10^9^/L) | 215.5 (138.5 - 281.5) | 203.8 (145.7 - 277.6) | 186.5 (126.5 - 249.0) | 0.422 |
| **Secondary Endpoints** |  |  |  |  |
| 28-d mortality (%) | 48 (17) | 121 (26) | 36 (46) | 0.006 |
| VFDs-28 (days) | 20.2 (10.3 - 24.7) | 18.9 (0.0 - 24.2) | 20.0 (0.0 - 25.1) | 0.022 |
| Length of MV (days) | 5.6 (3.1 - 10.6) | 5.1 (2.7 - 10.1) | 2.9 (2.1 - 4.7) | 0.642 |
| Length of ICU stay (days) | 10.7 (6.0 - 17.2) | 9.9 (5.2 - 19.2) | 4.2 (2.8 - 7.8) | 0.326 |
| **Switch characteristics** |  |  |  |  |
| Time between ICU admission and switch attempt (days) | 2.5 (1.2 - 5.3) | 1.5 (0.8 - 2.9) | - | <0.001 |
| Time between switch attempt and switch failure (hours) | - | 10.5 (2.8 - 25.2) | - | - |
| Number of secondary switch attempts (n) | - | 2 (1 - 4) | - | - |

Table E10: Results of the **before switch analysis** in the **MIMIC-IV cohort**. Time-varying variables sampled at the moment of a switch attempt (ie, switch samples). Data are in median (IQR). PaO_2_=arterial oxygen pressure, PaCO_2_=Partial pressure of carbon dioxide, FiO_2_=Fraction of inspired oxygen, SpO_2_=oxygen saturation, HCO_3_^-^ = bicarbonate, PEEP= Positive end-expiratory pressure, Pplat=pleateau pressure, ΔP=driving pressure, Pmean=mean airway pressure, Ppeak=peak airway pressure, C_RS_=respiratory system compliance, RR=respiratory rate, DBP=diastolic blood pressure, SBP=systolic blood pressure, MAP=mean arterial pressure.

|  | Successful switch  (n=962) | Failed switch  (n=2,452) | P value | Missingness (% successful, % failed) |
| --- | --- | --- | --- | --- |
| **Gas exchange parameters** |  |  |  |  |
| PaO_2_ (mmHg) | 111.0 (91.0; 137.0) | 105.0 (86.0; 134.0) | 0.32 | 18, 18 |
| PaCO_2_ (mmHg) | 38.0 (34.0; 42.0) | 39.0 (35.0; 44.0) | 0.002 | 18, 17 |
| PF-ratio |  |  |  |  |
| All | 234 (181; 288) | 216 (159; 282) | 0.177 | 49, 51 |
| Measured at PEEP ≤ 5 cmH_2_O | 253 (201; 292) | 250 (184; 307) | 0.653 |  |
| Measured at PEEP 6-10 cmH_2_O | 224 (174; 285) | 202 (154; 266) | 0.046 |  |
| Measured at PEEP > 10 cmH_2_O | 193 (136; 256) | 176 (124; 222) | 0.018 |  |
| pH | 7.4 (7.36; 7.44) | 7.39 (7.34; 7.43) | <0.001 | 18, 17 |
| Base excess (mmol/L) | 0.0 (-2.0; 2.0) | 0.0 (-4.0; 2.0) | <0.001 | 18, 17 |
| Lactic acid (mmol/L) | 1.6 (1.2; 2.2) | 1.7 (1.2; 2.6) | <0.001 | 34, 30 |
| HCO_3_^–^ (mmol/L) | 23.0 (20.0; 26.0) | 23.0 (20.0; 26.0) | 0.083 | 12, 13 |
| FiO_2_ (%) | 40 (40; 50) | 50 (40; 50) | <0.001 | 0, 0 |
| SpO_2_ (%) | 98 (96; 100) | 98 (96; 100) | <0.001 | 0, 0 |
| **Ventilatory parameters** |  |  |  |  |
| Pplat (cmH_2_O) | 19.0 (16.0; 22.0) | 20.0 (17.0; 23.0) | <0.001 | 10, 12 |
| ΔP (cmH_2_O) | 11.0 (9.0; 13.0) | 12.0 (9.0; 14.0) | <0.001 | 11, 13 |
| Pmean (cmH_2_O) | 11.0 (9.0; 13.0) | 12.0 (9.0; 14.0) | <0.001 | 0, 0 |
| Ppeak (cmH_2_O) | 22.0 (19.0; 26.0) | 24.0 (20.0; 28.0) | <0.001 | 0, 0 |
| PEEP (cmH_2_O) | 5.0 (5.0; 10.0) | 8.0 (5.0; 10.0) | <0.001 | 0, 0 |
| RR (breaths/min) | 19 (16; 22) | 20 (16; 24) | <0.001 | 0, 0 |
| Minute volume (L/min) | 8.8 (7.4; 10.4) | 9.2 (7.6; 11.0) | <0.001 | 0, 0 |
| C_RS_ (mL/cmH_2_O) |  |  |  |  |
| All | 41 (33; 51) | 39 (31; 50) | 0.035 | 11, 14 |
| Measured at PEEP ≤ 5 cmH_2_O | 40 (32; 51) | 38 (30; 47) | 0.004 |  |
| Measured at PEEP 6-10 cmH_2_O | 42 (34; 52) | 40 (32; 52) | 0.523 |  |
| Measured at PEEP > 10 cmH_2_O | 42 (34; 56) | 42 (32; 55) | 0.506 |  |
| **Inflammatory markers** |  |  |  |  |
| WBC (10^9^/L) | 11.9 (8.9; 16.4) | 12.2 (8.7; 17.3) | 0.056 | 16, 18 |
| **Other parameters** |  |  |  |  |
| Heart rate (bpm) | 84 (73; 95) | 86 (75; 100) | <0.001 | 0, 0 |
| Temperature (°C) | 37.1 (36.7; 37.4) | 37.1 (36.7; 37.5) | 0.788 | 15, 11 |
| MAP (mmHg) | 76 (68; 85) | 75 (68; 85) | 0.736 | 22, 27 |

Table E11: Results of the **before switch analysis** in the **AmsterdamUMCdb cohort**. Time-varying variables sampled at the moment of a switch attempt (ie, switch samples). Data are in median (IQR). PaO_2_=arterial oxygen pressure, PaCO_2_=Partial pressure of carbon dioxide, FiO_2_=Fraction of inspired oxygen, SpO_2_=oxygen saturation, HCO_3_^-^ = bicarbonate, PEEP= Positive end-expiratory pressure, Pplat=pleateau pressure, ΔP=driving pressure, Pmean=mean airway pressure, Ppeak=peak airway pressure, C_RS_=respiratory system compliance, RR=respiratory rate, DBP=diastolic blood pressure, SBP=systolic blood pressure, MAP=mean arterial pressure.

|  | Successful switch  (n=959) | Failed switch (n=1,617) | P value | Missingness (% successful, % failed) |
| --- | --- | --- | --- | --- |
| **Gas exchange parameters** |  |  |  |  |
| PaO_2_ (mmHg) | 94.0 (79.0; 116.0) | 97.5 (81.0; 120.0) | 0.005 | 1, 1 |
| PaCO_2_ (mmHg) | 40.0 (36.0; 44.0) | 40.0 (36.0; 45.0) | 0.69 | 1, 1 |
| PF-ratio |  |  |  | 1, 2 |
| All | 220 (171; 276) | 222 (171; 290) | 0.05 |  |
| Measured at PEEP ≤ 5 cmH_2_O | 264 (208; 319) | 282 (211; 355) | 0.084 |  |
| Measured at PEEP 6-10 cmH_2_O | 215 (170; 270) | 224 (176; 285) | 0.052 |  |
| Measured at PEEP > 10 cmH_2_O | 200 (153; 242) | 186 (148; 235) | 0.052 |  |
| pH | 7.37 (7.33; 7.42) | 7.37 (7.32; 7.41) | 0.004 | 1, 1 |
| Base excess (mmol/L) | 2.2 (0.3; 4.6) | 1.7 (-1.4; 4.5) | <0.001 | 1, 1 |
| Lactic acid (mmol/L) | 1.6 (1.1; 2.5) | 1.9 (1.3; 3.0) | <0.001 | 47, 47 |
| HCO_3_^–^ (mmol/L) | 22.9 (20.4; 25.5) | 22.5 (20.0; 25.1) | 0.321 | 1, 1 |
| FiO_2_ (%) | 41 (40; 49) | 41 (40; 50) | 0.001 | 0, 0 |
| SpO_2_ (%) | 98 (96; 99) | 98 (96; 99) | 0.326 | 0, 0 |
| **Ventilatory parameters** |  |  |  |  |
| Ppeak (cmH_2_O) | 22.0 (18.0; 26.0) | 23.0 (19.0; 26.0) | 0.001 | 0, 0 |
| PEEP (cmH_2_O) | 8.0 (6.0; 10.0) | 8.0 (6.0; 11.0) | 0.831 | 1, 0 |
| RR (breaths/min) | 18 (15; 21) | 18 (15; 22) | 0.328 | 0, 0 |
| Minute volume (L/min) | 8.8 (7.3; 10.3) | 8.4 (7.0; 10.3) | 0.753 | 0, 0 |
| **Inflammatory markers** |  |  |  |  |
| WBC (10^9^/L) | 11.9 (9.0; 16.4) | 12.0 (8.7; 16.9) | 0.70 | 22, 21 |
| **Other parameters** |  |  |  |  |
| Heart rate (bpm) | 84 (72; 97) | 84 (71; 98) | 0.703 | 0, 0 |
| Temperature (°C) | 37.0 (36.6; 37.0) | 37.0 (36.3; 37.0) | 0.006 | 34, 44 |
| MAP (mmHg) | 81 (72; 91) | 80 (71; 90) | 0.033 | 0, 0 |

Table E12: Results of the **before switch analysis** in the **Erasmus Medical Center cohort**. Time-varying variables sampled at the moment of a switch attempt (ie, switch samples). Data are in median (IQR). PaO_2_=arterial oxygen pressure, PaCO_2_=Partial pressure of carbon dioxide, FiO_2_=Fraction of inspired oxygen, SpO_2_=oxygen saturation, HCO_3_^-^ = bicarbonate, PEEP= Positive end-expiratory pressure, Pplat=pleateau pressure, ΔP=driving pressure, Pmean=mean airway pressure, Ppeak=peak airway pressure, C_RS_=respiratory system compliance, RR=respiratory rate, DBP=diastolic blood pressure, SBP=systolic blood pressure, MAP=mean arterial pressure.

|  | Successful switch  (n=270) | Failed switch (n=455) | P value | Missingness (% successful, % failed) |
| --- | --- | --- | --- | --- |
| **Gas exchange parameters** |  |  |  |  |
| PaO_2_ (mmHg) | 84.0 (75.0; 96.0) | 85.5 (75.8; 99.8) | 0.006 | 0, 1 |
| PaCO_2_ (mmHg) | 41.3 (37.5; 45.0) | 40.5 (36.0; 45.0) | 0.2 | 0, 1 |
| PF-ratio |  |  |  | 5, 10 |
| All | 225 (175; 288) | 220 (172; 289) | 0.505 |  |
| Measured at PEEP ≤ 5 cmH_2_O | 264 (190; 323) | 269 (220; 363) | 0.757 |  |
| Measured at PEEP 6-10 cmH_2_O | 229 (178; 292) | 225 (175; 288) | 0.623 |  |
| Measured at PEEP > 10 cmH_2_O | 219 (163; 278) | 191 (145; 260) | 0.033 |  |
| Base excess (mmol/L) | 1.4 (-1.7; 4.3) | -0.2 (-3.6; 2.9) | <0.001 | 0, 1 |
| Lactic acid (mmol/L) | 1.2 (0.9; 1.7) | 1.4 (1.0; 2.2) | 0.002 | 0, 1 |
| FiO_2_ (%) | 39 (30; 45) | 40 (30; 50) | 0.03 | 1, 3 |
| SpO_2_ (%) | 96 (95; 98) | 97 (95; 98) | 0.804 | 0, 1 |
| **Ventilatory parameters** |  |  |  |  |
| Pplat (cmH_2_O) | 21.0 (18.0; 24.5) | 22.0 (19.0; 25.0) | 0.624 | 53, 50 |
| ΔP (cmH_2_O) | 11.2 (10.0; 14.0) | 12.0 (9.7; 14.2) | 0.323 | 54, 50 |
| Ppeak (cmH_2_O) | 22.0 (19.0; 26.0) | 22.0 (19.0; 26.0) | 0.938 | 1, 5 |
| PEEP (cmH_2_O) | 10.0 (8.0; 12.0) | 10.0 (7.8; 12.0) | 0.275 | 9, 14 |
| RR (breaths/min) | 22 (18; 26) | 20 (18; 25) | 0.23 | 0, 1 |
| C_RS_ (mL/cmH_2_O) |  |  |  | 55, 51 |
| All | 42 (32; 54) | 40 (32; 53) | 0.032 |  |
| Measured at PEEP ≤ 5 cmH_2_O | 39 (33; 50) | 41 (24; 57) | 0.902 |  |
| Measured at PEEP 6-10 cmH_2_O | 42 (31; 53) | 36 (29; 47) | 0.007 |  |
| Measured at PEEP > 10 cmH_2_O | 47 (34; 61) | 45 (39; 59) | 0.332 |  |
| **Other parameters** |  |  |  |  |
| Temperature (°C) | 37.0 (36.6; 37.5) | 36.8 (36.3; 37.4) | 0.005 | 13, 12 |

Table E13: Results of the **after switch analysis** in the **MIMIC-IV cohort**. Δ_3h_ values of the included time-varying variables. Data are mean (SD). PaO_2_=arterial oxygen pressure, PaCO_2_=Partial pressure of carbon dioxide, PEEP= Positive end-expiratory pressure, FiO_2_=Fraction of inspired oxygen, SpO_2_=oxygen saturation, Pplat=pleateau pressure, ΔP=driving pressure, Ppeak=peak airway pressure, RR= respiratory rate, DBP=diastolic blood pressure, SBP=systolic blood pressure, MAP=mean arterial pressure.

|  | Successful switch (n=916) | Failed switch  (n=2,005) | P value | Missingness (% successful, % failed) |
| --- | --- | --- | --- | --- |
| **Gas exchange parameters** |  |  |  |  |
| Δ_3h_ PaO_2_ (mmHg) | -14.6 (50.3) | -11.9 (54.4) | 0.491 | 71, 74 |
| Δ_3h_ PaCO_2_ (mmHg) | -0.1 (5.0) | 1.1 (6.7) | 0.01 | 71, 74 |
| Δ_3h_ PF-ratio | -9 (104) | -12 (120) | 0.892 | 94, 96 |
| Δ_3h_ pH | 0.0 (0.041) | -0.009 (0.054) | 0.013 | 70, 74 |
| Δ_3h_ Base excess (mmol/L) | -0.0 (1.6) | -0.1 (1.9) | 0.627 | 71, 74 |
| Δ_3h_ FiO_2_ (%) | -5 (12) | -4 (15) | 0.433 | 70, 80 |
| Δ_3h_ SpO_2_ (%) | 0 (2) | 0 (3) | 0.797 | 0, 0 |
| **Ventilatory parameters** |  |  |  |  |
| Δ_3h_ Pmean (cmH_2_O) | -2.2 (2.6) | -2.0 (2.8) | 0.427 | 75, 83 |
| Δ_3h_ Ppeak (cmH_2_O) | -6.1 (6.0) | -6.1 (6.1) | 0.997 | 75, 84 |
| Δ_3h_ RR (breaths/min) | -1 (7) | -1 (6) | 0.785 | 0, 0 |
| Δ_3h_ Minute volume (L/min) | 0.0 (2.1) | -0.2 (2.7) | 0.349 | 74, 83 |
| Δ_3h_ Tidal volume (mL) | 33 (141) | 59 (177) | 0.067 | 74, 83 |
| **Other parameters** |  |  |  |  |
| Δ_3h_ Heart rate (bpm) | 2 (11) | 2 (12) | 0.432 | 0, 0 |
| Δ_3h_ Temperature (°C) | 0.1 (0.5) | 0.1 (0.6) | 0.942 | 66, 68 |
| Δ_3h_ MAP (mmHg) | 0 (16) | 0 (16) | 0.665 | 24, 30 |

Table E14: Results of the **after switch analysis** in the **AmsterdamUMCdb cohort**. Δ_3h_ values of the included time-varying variables. Data are mean (SD). PaO_2_=arterial oxygen pressure, PaCO_2_=Partial pressure of carbon dioxide, PEEP= Positive end-expiratory pressure, FiO_2_=Fraction of inspired oxygen, SpO_2_=oxygen saturation, Pplat=pleateau pressure, ΔP=driving pressure, Ppeak=peak airway pressure, RR= respiratory rate, DBP=diastolic blood pressure, SBP=systolic blood pressure, MAP=mean arterial pressure.

|  | Successful switch (n=933) | Failed switch  (n=1,177) | P value | Missingness (% successful, % failed) |
| --- | --- | --- | --- | --- |
| **Gas exchange parameters** |  |  |  |  |
| Δ_3h_ PaO_2_ (mmHg) | -3.9 (36.9) | -6.9 (35.4) | 0.157 | 46, 44 |
| Δ_3h_ PaCO_2_ (mmHg) | 0.4 (6.0) | 0.7 (6.5) | 0.445 | 46, 44 |
| Δ_3h_ PF-ratio | -2 (73) | -12 (78) | 0.03 | 48, 46 |
| Δ_3h_ pH | -0.001 (0.048) | -0.004 (0.054) | 0.346 | 46, 44 |
| Δ_3h_ Base excess (mmol/L) | 0.0 (1.7) | -0.1 (2.4) | 0.268 | 47, 44 |
| Δ_3h_ FiO_2_ (%) | -1 (7) | 0 (9) | 0.054 | 0, 1 |
| Δ_3h_ SpO_2_ (%) | 0 (5) | -1 (6) | 0.027 | 0, 0 |
| **Ventilatory parameters** |  |  |  |  |
| Δ_3h_ Ppeak (cmH_2_O) | -2.8 (4.7) | -1.8 (4.7) | <0.001 | 0, 1 |
| Δ_3h_ PEEP (cmH_2_O) | -0.4 (1.7) | -0.2 (1.4) | 0.001 | 1, 1 |
| Δ_3h_ RR (breaths/min) | -1 (7) | -1 (7) | 0.306 | 0, 1 |
| Δ_3h_ Minute volume (L/min) | -0.2 (4.0) | -0.6 (5.8) | 0.101 | 0, 1 |
| Δ_3h_ Tidal volume (mL) | 51 (644) | 23 (505) | 0.265 | 0, 1 |
| **Other parameters** |  |  |  |  |
| Δ_3h_ Heart rate (bpm) | 3 (13) | 4 (15) | 0.565 | 0, 1 |
| Δ_3h_ Temperature (°C) | 0.2 (1.0) | 0.1 (0.8) | 0.468 | 54, 59 |
| Δ_3h_ MAP (mmHg) | 0 (16) | 1 (15) | 0.777 | 1, 1 |

Table E15: Results of the **after switch analysis** in the **Erasmus Medical Center cohort**. Δ_3h_ values of the included time-varying variables. Data are mean (SD). PaO_2_=arterial oxygen pressure, PaCO_2_=Partial pressure of carbon dioxide, PEEP= Positive end-expiratory pressure, FiO_2_=Fraction of inspired oxygen, SpO_2_=oxygen saturation, Pplat=pleateau pressure, ΔP=driving pressure, Ppeak=peak airway pressure, RR= respiratory rate.

|  | Successful switch (n=252) | Failed switch  (n=338) | P value | Missingness (% successful, % failed) |
| --- | --- | --- | --- | --- |
| **Gas exchange parameters** |  |  |  |  |
| Δ_3h_ PaO_2_ (mmHg) | 0.5 (32.3) | -6.0 (45.4) | 0.112 | 32, 26 |
| Δ_3h_ PaCO_2_ (mmHg) | -0.2 (5.2) | 0.6 (6.5) | 0.226 | 31, 26 |
| Δ_3h_ PF-ratio | 2 (54) | -9 (115) | 0.372 | 63, 62 |
| Δ_3h_ Base excess (mmol/L) | 0.2 (1.2) | -0.1 (1.7) | 0.072 | 32, 26 |
| Δ_3h_ FiO_2_ (%) | 0 (11) | 0 (11) | 0.869 | 6, 12 |
| Δ_3h_ SpO_2_ (%) | 0 (2) | 0 (3) | 0.34 | 3, 6 |
| **Ventilatory parameters** |  |  |  |  |
| Δ_3h_ Ppeak (cmH_2_O) | -2.2 (4.4) | -2.3 (4.4) | 0.704 | 6, 14 |
| Δ_3h_ PEEP (cmH_2_O) | -0.3 (1.4) | -0.2 (1.6) | 0.728 | 13, 23 |
| Δ_3h_ RR (breaths/min) | -2 (8) | -2 (8) | 0.836 | 3, 7 |
| **Other parameters** |  |  |  |  |
| Δ_3h_ Temperature (°C) | 0.2 (0.6) | 0.1 (1.0) | 0.314 | 49, 52 |

Table E16: **Baseline characteristics and endpoints**, grouped by the success or failure of the first switch attempt, examining **only actual switches from controlled to assisted ventilation modes (n= 5,492)**. Data are in median (IQR) or number (percentage). PaO_2_=arterial oxygen pressure, PaCO_2_=Partial pressure of carbon dioxide, ΔP=driving pressure, C_RS_=respiratory system compliance, MAP=mean arterial pressure, VFDs=ventilator-free days, MV=mechanical ventilation, ICU=intensive care unit.

|  | Successful switch (n=1,872) | Failed switch (n=3,620) | P value |
| --- | --- | --- | --- |
| **Demographics** |  |  |  |
| Age group, n (%) |  |  |  |
| 18-39 | 159 (8) | 345 (10) | 0.218 |
| 40-49 | 175 (9) | 344 (10) | 0.884 |
| 50-59 | 306 (16) | 619 (17) | 0.494 |
| 60-69 | 393 (21) | 801 (22) | 0.351 |
| 70-79 | 415 (22) | 748 (21) | 0.198 |
| 80+ | 221 (12) | 437 (12) | 0.793 |
| Female sex (%) | 712(38.0) | 1359(37.5) | 0.907 |
| **Gas exchange** |  |  |  |
| PF-ratio | 216.5 (162.1 - 278.1) | 209.6 (156.9 - 278.5) | 0.513 |
| PaO_2_ (mmHg) | 113.1 (92.5 - 144.2) | 112.4 (92.7 - 143.6) | 0.275 |
| PaCO_2_ (mmHg) | 40.0 (36.4 - 44.3) | 40.5 (36.5 - 45.0) | 0.002 |
| pH | 7.35 (7.31 - 7.4) | 7.34 (7.29 - 7.39) | <0.001 |
| **Respiratory mechanics** |  |  |  |
| ΔP (cmH_2_O) | 11.7 (9.8 - 14.0) | 12.0 (10.0 - 14.5) | <0.001 |
| C_RS_ (mL/cmH_2_O) | 40.3 (32.3 - 50.3) | 38.7 (31.5 - 47.7) | 0.434 |
| **SOFA components** |  |  |  |
| MAP (mmHg) | 73.9 (67.0 - 82.2) | 73.0 (67.0 - 80.1) | 0.053 |
| Bilirubin (µmol/L) | 11.0 (6.8 - 20.5) | 11.3 (6.8 - 20.6) | 0.196 |
| Creatinine (µmol/L) | 97.2 (73.7 - 141.4) | 100.8 (75.1 - 150.3) | 0.292 |
| Platelet count (10^9^/L) | 189.0 (132.3 - 254.7) | 183.7 (126.0 - 247.7) | 0.906 |
| **Secondary Endpoints** |  |  |  |
| 28-d mortality (%) | 320 (17) | 985 (27) | <0.0001 |
| VFDs-28 (days) | 22.3 (12.2 - 25.2) | 16.5 (0.0 - 22.8) | <0.0001 |
| Length of MV (days) | 4.7 (2.7 - 8.9) | 6.9 (3.9 - 12.6) | <0.0001 |
| Length of ICU stay (days) | 7.7 (4.8 - 13.5) | 9.9 (5.9 - 17.4) | <0.0001 |
| **Switch characteristics** |  |  |  |
| Time between ICU admission and switch attempt (days) | 1.7 (0.7 - 2.9) | 1.3 (0.5 - 2.5) | <0.001 |
| Time between switch attempt and switch failure (hours) | - | 9.0 (4.0 - 21.5) | - |
| Number of secondary switch attempts (n) | - | 2 (1 - 4) | - |

Table E17: **Baseline characteristics and endpoints**, grouped by the success or failure of the first switch attempt, examining **only switches observed as changes in respiratory rate within combined modes (n= 1,223)**. Data are in median (IQR) or number (percentage). PaO_2_=arterial oxygen pressure, PaCO_2_=Partial pressure of carbon dioxide, ΔP=driving pressure, C_RS_=respiratory system compliance, MAP=mean arterial pressure, VFDs=ventilator-free days, MV=mechanical ventilation, ICU=intensive care unit.

|  | Successful switch (n=319) | Failed switch  (n=904) | P value |
| --- | --- | --- | --- |
| **Demographics** |  |  |  |
| Age group, n (%) |  |  |  |
| 18-39 | 18 (6) | 74 (8) | 0.174 |
| 40-49 | 27 (8) | 69 (8) | 0.629 |
| 50-59 | 51 (16) | 165 (18) | 0.394 |
| 60-69 | 69 (22) | 178 (20) | 0.466 |
| 70-79 | 54 (17) | 167 (18) | 0.555 |
| 80+ | 33 (10) | 122 (13) | 0.17 |
| Female sex (%) | 109(34.2) | 322(35.6) | 0.634 |
| **Gas exchange** |  |  |  |
| PF-ratio | 211.0 (164.1 - 273.4) | 211.1 (151.7 - 276.4) | 0.615 |
| PaO_2_ (mmHg) | 102.7 (87.6 - 120.8) | 109.9 (89.7 - 140.2) | <0.001 |
| PaCO_2_ (mmHg) | 40.3 (36.9 - 44.3) | 40.7 (36.2 - 45.2) | 0.465 |
| pH | 7.34 (7.28 - 7.39) | 7.34 (7.29 - 7.4) | 0.457 |
| **Respiratory mechanics** |  |  |  |
| ΔP (cmH_2_O) | 12.0 (9.7 - 14.4) | 12.5 (10.0 - 15.0) | 0.184 |
| C_RS_ (mL/cmH_2_O) | 44.3 (32.3 - 52.8) | 39.8 (30.4 - 50.7) | 0.113 |
| **SOFA components** |  |  |  |
| MAP (mmHg) | 72.9 (68.8 - 78.1) | 73.3 (66.6 - 81.4) | 0.624 |
| Bilirubin (µmol/L) | 10.5 (6.8 - 17.1) | 12.0 (6.8 - 21.4) | 0.173 |
| Creatinine (µmol/L) | 92.3 (70.5 - 126.0) | 101.7 (70.7 - 154.7) | 0.015 |
| Platelet count (10^9^/L) | 213.5 (154.5 - 264.7) | 190.5 (131.9 - 257.8) | 0.015 |
| **Secondary Endpoints** |  |  |  |
| 28-d mortality (%) | 47 (14) | 274 (30) | <0.0001 |
| VFDs-28 (days) | 21.7 (13.5 - 25.0) | 15.5 (0.0 - 22.8) | <0.0001 |
| Length of MV (days) | 5.2 (2.8 - 9.3) | 6.8 (3.8 - 12.5) | <0.0001 |
| Length of ICU stay (days) | 8.3 (5.1 - 14.0) | 9.9 (5.8 - 17.0) | <0.0001 |
| **Switch characteristics** |  |  |  |
| Time between ICU admission and switch attempt (days) | 1.9 (1.0 - 2.8) | 1.3 (0.5 - 2.5) | 0.015 |
| Time between switch attempt and switch failure (hours) | - | 5.0 (3.6 - 12.0) | - |
| Number of secondary switch attempts (n) | - | 2 (1 - 4) | - |

Table E18: Results of the **before switch analysis**, examining **only actual switches from controlled to assisted ventilation modes (n= 5,492)**. Time-varying variables sampled at the moment of a switch attempt (ie, switch samples). Data are in median (IQR). PaO_2_=arterial oxygen pressure, PaCO_2_=Partial pressure of carbon dioxide, FiO_2_=Fraction of inspired oxygen, SpO_2_=oxygen saturation, HCO_3_^-^ = bicarbonate, PEEP= Positive end-expiratory pressure, Pplat=pleateau pressure, ΔP=driving pressure, Pmean=mean airway pressure, Ppeak=peak airway pressure, C_RS_=respiratory system compliance, RR=respiratory rate, V_min_=Minute volume, DBP=diastolic blood pressure, SBP=systolic blood pressure, MAP=mean arterial pressure.

|  | Successful switch  (n=1,872) | Failed switch  (n=3,620) | P value | Missingness (% successful, % failed) |
| --- | --- | --- | --- | --- |
| **Gas exchange parameters** |  |  |  |  |
| PaO_2_ (mmHg) | 100.0 (82.0; 123.0) | 100.0 (83.0; 126.0) | 0.046 | 9, 10 |
| PaCO_2_ (mmHg) | 39.8 (35.0; 44.0) | 40.0 (35.0; 44.0) | 0.012 | 9, 10 |
| PF-ratio |  |  |  |  |
| All | 225 (175; 283) | 221 (170; 288) | 0.674 | 25, 29 |
| Measured at PEEP ≤ 5 cmH_2_O | 258 (206; 306) | 262 (198; 340) | 0.267 |  |
| Measured at PEEP 6-10 cmH_2_O | 221 (172; 282) | 220 (170; 282) | 0.717 |  |
| Measured at PEEP > 10 cmH_2_O | 202 (156; 254) | 190 (148; 240) | 0.008 |  |
| pH | 7.39 (7.34; 7.43) | 7.38 (7.33; 7.42) | <0.001 | 20, 19 |
| Base excess (mmol/L) | 0.8 (-1.5; 3.6) | 0.0 (-3.0; 3.0) | <0.001 | 9, 10 |
| Lactic acid (mmol/L) | 1.6 (1.1; 2.3) | 1.7 (1.2; 2.7) | <0.001 | 38, 35 |
| HCO_3_^–^ (mmol/L) | 23.0 (20.3; 25.7) | 22.7 (20.0; 25.4) | 0.174 | 17, 16 |
| FiO_2_ (%) | 41 (40; 50) | 41 (40; 50) | <0.001 | 0, 0 |
| SpO_2_ (%) | 98 (96; 99) | 98 (96; 99) | 0.09 | 0, 0 |
| **Ventilatory parameters** |  |  |  |  |
| Pplat (cmH_2_O) | 19.0 (16.0; 22.0) | 20.0 (17.0; 23.0) | <0.001 | 51, 49 |
| ΔP (cmH_2_O) | 11.0 (9.0; 13.0) | 12.0 (9.0; 14.0) | <0.001 | 52, 49 |
| Pmean (cmH_2_O) | 11.0 (9.0; 14.0) | 11.0 (9.0; 14.0) | <0.001 | 52, 47 |
| Ppeak (cmH_2_O) | 23.0 (19.0; 26.0) | 23.0 (20.0; 27.0) | <0.001 | 0, 0 |
| PEEP (cmH_2_O) | 8.0 (5.0; 10.0) | 8.0 (5.0; 10.0) | 0.951 | 1, 1 |
| RR (breaths/min) | 19 (16; 23) | 19 (16; 23) | 0.277 | 0, 0 |
| Minute volume (L/min) | 8.8 (7.4; 10.4) | 8.8 (7.3; 10.6) | 0.233 | 11, 9 |
| C_RS_ (mL/cmH_2_O) |  |  |  |  |
| All | 41 (33; 52) | 39 (31; 50) | 0.002 | 52, 50 |
| Measured at PEEP ≤ 5 cmH_2_O | 41 (32; 51) | 38 (30; 47) | 0.007 |  |
| Measured at PEEP 6-10 cmH_2_O | 42 (33; 52) | 39 (31; 50) | 0.069 |  |
| Measured at PEEP > 10 cmH_2_O | 42 (34; 58) | 44 (35; 58) | 0.241 |  |
| **Inflammatory markers** |  |  |  |  |
| WBC (10^9^/L) | 11.9 (9.0; 16.5) | 12.1 (8.6; 17.1) | 0.227 | 27, 25 |
| **Other parameters** |  |  |  |  |
| Heart rate (bpm) | 83 (72; 95) | 85 (73; 98) | 0.001 | 11, 9 |
| Temperature (°C) | 37.0 (36.7; 37.3) | 37.0 (36.6; 37.4) | 0.61 | 26, 26 |
| MAP (mmHg) | 79 (70; 88) | 78 (69; 88) | 0.263 | 21, 23 |

Table E19: Results of the **before switch analysis**, examining **only switches observed as changes in respiratory rate within combined modes (n= 1,223)**. Time-varying variables sampled at the moment of a switch attempt (ie, switch samples). Data are in median (IQR). PaO_2_=arterial oxygen pressure, PaCO_2_=Partial pressure of carbon dioxide, FiO_2_=Fraction of inspired oxygen, SpO_2_=oxygen saturation, HCO_3_^-^ = bicarbonate, PEEP= Positive end-expiratory pressure, Pplat=pleateau pressure, ΔP=driving pressure, Pmean=mean airway pressure, Ppeak=peak airway pressure, C_RS_=respiratory system compliance, RR=respiratory rate, DBP=diastolic blood pressure, SBP=systolic blood pressure, MAP=mean arterial pressure.

|  | Successful switch  (n=319) | Failed switch  (n=904) | P value | Missingness (% successful, % failed) |
| --- | --- | --- | --- | --- |
| **Gas exchange parameters** |  |  |  |  |
| PaO_2_ (mmHg) | 90.0 (78.6; 110.2) | 95.3 (78.8; 120.0) | 0.001 | 4, 9 |
| PaCO_2_ (mmHg) | 41.3 (37.0; 46.0) | 40.0 (35.0; 45.0) | 0.066 | 4, 9 |
| PF-ratio |  |  |  |  |
| All | 222 (172; 285) | 212 (160; 278) | 0.583 | 11, 31 |
| Measured at PEEP ≤ 5 cmH_2_O | 266 (200; 320) | 258 (189; 308) | 0.889 |  |
| Measured at PEEP 6-10 cmH_2_O | 213 (169; 263) | 206 (166; 267) | 0.807 |  |
| Measured at PEEP > 10 cmH_2_O | 207 (150; 247) | 159 (120; 200) | <0.001 |  |
| pH | 7.38 (7.33; 7.418) | 7.38 (7.32; 7.43) | 0.646 | 4, 9 |
| Base excess (mmol/L) | 1.8 (0.0; 4.1) | 0.0 (-3.0; 3.0) | <0.001 | 20, 26 |
| Lactic acid (mmol/L) | 1.4 (1.0; 1.9) | 1.7 (1.2; 2.7) | <0.001 | 24, 24 |
| HCO_3_^–^ (mmol/L) | 23.0 (20.5; 25.8) | 23.0 (20.0; 25.6) | 0.159 | 0, 0 |
| FiO_2_ (%) | 40 (39; 50) | 50 (40; 54) | <0.001 | 0, 0 |
| SpO_2_ (%) | 97 (95; 99) | 97 (95; 99) | 0.066 | 4, 9 |
| **Ventilatory parameters** |  |  |  |  |
| Pplat (cmH_2_O) | 20.0 (17.0; 23.0) | 21.0 (17.0; 25.0) | 0.042 | 72, 41 |
| ΔP (cmH_2_O) | 12.0 (10.0; 14.0) | 12.0 (10.0; 15.0) | 0.364 | 72, 41 |
| Pmean (cmH_2_O) | 11.0 (9.0; 13.0) | 12.0 (9.9; 15.0) | <0.001 | 76, 39 |
| Ppeak (cmH_2_O) | 19.0 (15.0; 23.0) | 23.0 (19.0; 27.0) | <0.001 | 0, 0 |
| PEEP (cmH_2_O) | 8.0 (5.0; 10.0) | 8.0 (5.0; 10.0) | 0.428 | 2, 3 |
| RR (breaths/min) | 16 (12; 20) | 20 (15; 24) | <0.001 | 0, 0 |
| Minute volume (L/min) | 8.6 (7.1; 10.3) | 9.2 (7.5; 11.3) | <0.001 | 21, 14 |
| C_RS_ (mL/cmH_2_O) |  |  |  |  |
| All | 40 (32; 50) | 39 (31; 50) | 0.134 | 72, 42 |
| Measured at PEEP ≤ 5 cmH_2_O | 38 (31; 47) | 38 (30; 46) | 0.471 |  |
| Measured at PEEP 6-10 cmH_2_O | 40 (31; 50) | 41 (31; 53) | 0.229 |  |
| Measured at PEEP > 10 cmH_2_O | 48 (38; 51) | 39 (31; 49) | 0.784 |  |
| **Inflammatory markers** |  |  |  |  |
| WBC (10^9^/L) | 11.4 (8.8; 15.2) | 12.2 (8.8; 17.6) | 0.023 | 39, 34 |
| **Other parameters** |  |  |  |  |
| Heart rate (bpm) | 89 (75; 102) | 88 (76; 102) | 0.952 | 21, 14 |
| Temperature (°C) | 37.0 (36.8; 37.0) | 37.0 (36.6; 37.4) | 0.978 | 7, 12 |
| MAP (mmHg) | 80 (72; 90) | 77 (69; 87) | 0.025 | 26, 31 |

Table E20: Results of the **after switch analysis**, examining **only actual switches from controlled to assisted ventilation modes (n=4,620)**. Δ_3h_ values of the included time-varying variables. Data are mean (SD). PaO_2_=arterial oxygen pressure, PaCO_2_=Partial pressure of carbon dioxide, PEEP= Positive end-expiratory pressure, FiO_2_=Fraction of inspired oxygen, SpO_2_=oxygen saturation, Pplat=plateau pressure, ΔP=driving pressure, Ppeak=peak airway pressure, RR= respiratory rate, DBP=diastolic blood pressure, SBP=systolic blood pressure, MAP=mean arterial pressure.

|  | Successful switch (n=1,794) | Failed switch  (n=2,826) | P value | Missingness (% successful, % failed) |
| --- | --- | --- | --- | --- |
| **Gas exchange parameters** |  |  |  |  |
| Δ_3h_ PaO_2_ (mmHg) | -6.5 (41.1) | -8.7 (43.7) | 0.265 | 56, 59 |
| Δ_3h_ PaCO_2_ (mmHg) | 0.3 (5.6) | 0.9 (6.8) | 0.04 | 56, 59 |
| Δ_3h_ PF-ratio | -3 (78) | -14 (92) | 0.039 | 72, 75 |
| Δ_3h_ pH | -0.001 (0.047) | -0.008 (0.056) | 0.013 | 63, 65 |
| Δ_3h_ Base excess (mmol/L) | 0.0 (1.5) | -0.1 (2.2) | 0.1 | 56, 59 |
| Δ_3h_ FiO_2_ (%) | -2 (9) | -1 (11) | 0.02 | 33, 44 |
| Δ_3h_ SpO_2_ (%) | 0 (4) | -1 (4) | 0.005 | 1, 1 |
| **Ventilatory parameters** |  |  |  |  |
| Δ_3h_ Ppeak (cmH_2_O) | -0.4 (1.7) | -0.2 (1.7) | 0.002 | 35, 45 |
| Δ_3h_ RR (breaths/min) | -2 (7) | -1 (7) | 0.392 | 0, 1 |
| Δ_3h_ Minute volume (L/min) | -0.2 (3.9) | -0.5 (5.5) | 0.179 | 44, 53 |
| Δ_3h_ Tidal volume (mL) | 58 (511) | 41 (445) | 0.372 | 35, 46 |
| **Other parameters** |  |  |  |  |
| Δ_3h_ Heart rate (bpm) | 3 (12) | 3 (14) | 0.911 | 10, 9 |
| Δ_3h_ Temperature (°C) | 0.2 (0.6) | 0.1 (0.6) | 0.327 | 61, 63 |
| Δ_3h_ MAP (mmHg) | 0 (16) | 0 (16) | 0.775 | 22, 26 |

Table E21: Results of the **after switch analysis**, examining **only switches observed as changes in respiratory rate within combined modes (n=1,000)**. Δ_3h_ values of the included time-varying variables. Data are mean (SD). PaO_2_=arterial oxygen pressure, PaCO_2_=Partial pressure of carbon dioxide, PEEP= Positive end-expiratory pressure, FiO_2_=Fraction of inspired oxygen, SpO_2_=oxygen saturation, Pplat=pleateau pressure, ΔP=driving pressure, Ppeak=peak airway pressure, RR= respiratory rate, DBP=diastolic blood pressure, SBP=systolic blood pressure, MAP=mean arterial pressure.

|  | Successful switch (n=306) | Failed switch  (n=694) | P value | Missingness (% successful, % failed) |
| --- | --- | --- | --- | --- |
| **Gas exchange parameters** |  |  |  |  |
| Δ_3h_ PaO_2_ (mmHg) | -4.1 (38.7) | -7.9 (50.0) | 0.42 | 50, 61 |
| Δ_3h_ PaCO_2_ (mmHg) | -0.6 (5.5) | 0.4 (5.5) | 0.077 | 49, 61 |
| Δ_3h_ PF-ratio | 1 (57) | -3 (71) | 0.614 | 57, 80 |
| Δ_3h_ pH | 0.003 (0.04) | 0.001 (0.045) | 0.739 | 62, 69 |
| Δ_3h_ Base excess (mmol/L) | 0.2 (1.9) | 0.0 (1.6) | 0.428 | 50, 61 |
| Δ_3h_ FiO_2_ (%) | -0.1 (0.8) | -0.1 (1.0) | 0.827 | 61, 75 |
| Δ_3h_ SpO_2_ (%) | 0 (8) | -1 (12) | 0.062 | 24, 60 |
| **Ventilatory parameters** |  |  |  |  |
| Δ_3h_ Ppeak (cmH_2_O) | -0.3 (1.5) | 0.1 (1.5) | 0.008 | 25, 64 |
| Δ_3h_ RR (breaths/min) | 0 (7) | -1 (6) | 0.156 | 1, 1 |
| Δ_3h_ Minute volume (L/min) | 0.1 (2.2) | -0.5 (3.5) | 0.056 | 43, 75 |
| Δ_3h_ Tidal volume (mL) | 1 (696) | -5 (349) | 0.91 | 24, 62 |
| **Other parameters** |  |  |  |  |
| Δ_3h_ Heart rate (bpm) | 1 (12) | 1 (12) | 0.645 | 20, 12 |
| Δ_3h_ Temperature (°C) | 0.0 (1.3) | -0.0 (1.1) | 0.451 | 43, 64 |
| Δ_3h_ MAP (mmHg) | 0 (16) | -1 (14) | 0.214 | 26, 32 |

Table E22: Results of the **baseline analysis**, **distinguishing early and late failures**. Data are in median (IQR) or number (percentage). PaO_2_=arterial oxygen pressure, PaCO_2_=Partial pressure of carbon dioxide, ΔP=driving pressure, C_RS_=respiratory system compliance, MAP=mean arterial pressure, VFDs=ventilator-free days, MV=mechanical ventilation, ICU=intensive care unit.
*P-values are given for the comparison of distributions with early failed (n=2,239) and late failed (n=2,285) switch attempts.

| Variable | Successful switch (n=2,191) | Early failed switch attempts (n=2,239) | Late failed switch attempts (n=2,285) | P value* |
| --- | --- | --- | --- | --- |
| **Demographics** |  |  |  |  |
| Age group, n (%) |  |  |  |  |
| 18-39 | 177 (8) | 203 (9) | 216 (9) | 0.682 |
| 40-49 | 202 (9) | 206 (9) | 207 (9) | 0.877 |
| 50-59 | 357 (16) | 403 (18) | 381 (17) | 0.255 |
| 60-69 | 462 (21) | 475 (21) | 504 (22) | 0.493 |
| 70-79 | 469 (21) | 461 (21) | 454 (20) | 0.554 |
| 80+ | 254 (12) | 290 (13) | 269 (12) | 0.24 |
| Female sex (%) | 821(37.5) | 827(36.9) | 854(37.4) | 0.83 |
| **Gas exchange** |  |  |  |  |
| PaO_2_/FiO_2_ | 216 (163 - 278) | 207.2 (154.0 - 276.7) | 212.4 (156.5 - 279.1) | 0.292 |
| PaO_2_ (mmHg) | 111.0 (91.4 - 141.7) | 112.0 (91.6 - 144.6) | 111.7 (92.2 - 141.6) | 0.195 |
| PaCO_2_ (mmHg)^‡^ | 40.0 (36.5 - 44.3) | 40.7 (36.6 - 45.2) | 40.4 (36.3 - 45.1) | 0.006 |
| pH^††^ | 7.35 (7.31 - 7.4) | 7.34 (7.29 - 7.39) | 7.34 (7.3 - 7.39) | 0.18 |
| **Respiratory mechanics** |  |  |  |  |
| ΔP (cmH_2_O)^††^ | 11.7 (9.8 - 14.0) | 12.4 (10.2 - 14.7) | 12.0 (10.0 - 14.5) | 0.053 |
| C_RS_ (mL/cmH_2_O)^††^ | 40.6 (32.3 - 50.5) | 38.5 (31.0 - 47.5) | 39.2 (31.4 - 49.8) | 0.159 |
| **SOFA components** |  |  |  |  |
| Mean arterial pressure (mmHg)^†^ | 73.9 (67.3 - 81.9) | 73.1 (66.8 - 80.1) | 73.0 (66.9 - 80.8) | 0.992 |
| Bilirubin (µmol/L)^††^ | 11.0 (6.8 - 20.3) | 11.1 (6.8 - 21.0) | 11.8 (7.0 - 20.6) | 0.888 |
| Creatinine (µmol/L)^††^ | 97.2 (73.3 - 139.2) | 102.3 (74.8 - 154.7) | 99.3 (73.7 - 147.3) | 0.024 |
| Platelet count (10^9^/L) | 191.5 (135.0 - 256.0) | 185.3 (127.1 - 246.5) | 184.5 (127.2 - 253.7) | 0.851 |
| **Baseline severity scores** |  |  |  |  |
| APACHE-II score^†^ | 26.0 (21.0 - 32.0) | 26.0 (19.0 - 32.0) | 26.0 (21.0 - 32.0) | 0.554 |
| SAPS-II score^†^ | 44.0 (34.5 - 54.0) | 47.0 (38.0 - 58.0) | 46.0 (37.0 - 57.0) | 0.202 |
| **Secondary Endpoints** |  |  |  |  |
| 28-d mortality (%)^‡^ | 367 (16) | 601 (26) | 658 (28) | 0.144 |
| VFDs-28 (days)^‡^ | 22.2 (12.4 - 25.2) | 16.4 (0.0 - 23.2) | 16.3 (0.0 - 22.4) | 0.261 |
| Length of MV (days) | 4.8 (2.7 - 8.9) | 6.7 (3.8 - 12.7) | 7.1 (4.0 - 12.3) | 0.618 |
| Length of ICU stay (days) | 7.8 (4.8 - 13.6) | 9.3 (5.6 - 17.2) | 10.3 (6.2 - 17.3) | 0.292 |
| **Switch characteristics** |  |  |  |  |
| Time between ICU admission and switch attempt (days)^‡^ | 1.8 (0.8 – 2.9) | 1.3 (0.5 - 2.6) | 1.2 (0.5 - 2.5) | 0.223 |
| Time between switch attempt and switch failure (hours) | - | 4.0 (2.0 - 5.0) | 19.0 (12.0 - 35.0) | <0.001 |
| Number of secondary switch attempts (n) | - | 2.0 (1.0 - 4.0) | 2.0 (1.0 - 3.0) | <0.001 |

Table E23: Results of the **before switch analysis**, **distinguishing early and late failures**. Time-varying variables sampled at the moment of a switch attempt (ie, switch samples). *P-values are given for the comparison of distributions with early failed (n=2,239) and late failed (n=2,285) switch attempts. Data are in median (IQR). PaO_2_=arterial oxygen pressure, PaCO_2_=Partial pressure of carbon dioxide, PEEP= Positive end-expiratory pressure, FiO_2_=Fraction of inspired oxygen, SpO_2_=oxygen saturation, Pplat=pleateau pressure, ΔP=driving pressure, Ppeak=peak airway pressure, RR= respiratory rate, DBP=diastolic blood pressure, SBP=systolic blood pressure, MAP=mean arterial pressure.

|  | Successful switch attempts  (n=2,191) | Early failed switch attempts (n=2,239) | Late failed switch attempts (n=2,285) | P value* | Missingness (% successful, % early failed, % late failed) |
| --- | --- | --- | --- | --- | --- |
| **Gas exchange parameters** |  |  |  |  |  |
| PaO_2_ (mmHg) | 98.0 (81.2; 122.0) | 98.0 (81.8; 122.5) | 100.0 (83.0; 127.5) | 0,7 | 8, 11, 9 |
| PaCO_2_ (mmHg) | 40.0 (36.0; 44.0) | 40.0 (35.0; 45.0) | 40.0 (35.0; 44.0) | 0,102 | 8, 10, 9 |
| PF-ratio |  |  |  |  | 23, 30, 30 |
| All | 225 (174; 284) | 216 (162; 286) | 225 (173; 288) | 0,017 |  |
| Measured at PEEP ≤ 5 cmH_2_O | 258 (205; 312) | 258 (192; 333) | 262 (198; 330) | 0,637 |  |
| Measured at PEEP 6-10 cmH_2_O | 220 (172; 279) | 215 (165; 277) | 222 (174; 284) | 0,125 |  |
| Measured at PEEP > 10 cmH_2_O | 205 (154; 254) | 181 (135; 235) | 190 (148; 233) | 0,191 |  |
| pH | 7.39 (7.34; 7.43) | 7.38 (7.32; 7.43) | 7.38 (7.33; 7.43) | 0,109 | 20, 20, 20 |
| Base excess (mmol/L) | 1.0 (-1.2; 3.8) | 0.0 (-3.0; 3.0) | 0.2 (-3.0; 3.2) | 0,129 | 8, 10, 9 |
| Lactic acid (mmol/L) | 1.5 (1.1; 2.2) | 1.7 (1.2; 2.6) | 1.7 (1.2; 2.7) | 0,246 | 35, 35, 31 |
| HCO_3_^–^ (mmol/L) | 23.0 (20.3; 25.7) | 22.8 (20.0; 25.6) | 22.8 (20.0; 25.2) | 0,576 | 18, 17, 17 |
| FiO_2_ (%) | 41 (40; 50) | 45 (40; 50) | 41 (40; 50) | <0.001 | 0, 0, 0 |
| SpO_2_ (%) | 98 (96; 99) | 98 (96; 99) | 98 (96; 99) | 0,157 | 0, 0, 0 |
| **Ventilatory parameters** |  |  |  |  |  |
| Pplat (cmH_2_O) | 19.0 (16.0; 22.0) | 20.0 (17.0; 24.0) | 20.0 (17.0; 23.0) | 0,01 | 54, 47, 47 |
| ΔP (cmH_2_O) | 11.0 (9.0; 13.0) | 12.0 (10.0; 15.0) | 11.5 (9.0; 14.0) | <0.001 | 55, 48, 47 |
| Pmean (cmH_2_O) | 11.0 (9.0; 13.0) | 12.0 (9.2; 15.0) | 11.0 (9.0; 14.0) | <0.001 | 56, 45, 46 |
| Ppeak (cmH_2_O) | 22.0 (19.0; 26.0) | 24.0 (20.0; 28.0) | 23.0 (19.0; 26.8) | <0.001 | 0, 0, 1 |
| PEEP (cmH_2_O) | 8.0 (5.0; 10.0) | 8.0 (5.0; 10.0) | 8.0 (5.0; 10.0) | 0,004 | 1, 2, 2 |
| RR (breaths/min) | 18 (15; 22) | 20 (16; 23) | 19 (16; 23) | 0,386 | 0, 0, 0 |
| Minute volume (L/min) | 8.8 (7.4; 10.4) | 8.9 (7.3; 10.7) | 8.9 (7.4; 10.8) | 0,08 | 12, 9, 11 |
| C_RS_ (mL/cmH_2_O) |  |  |  |  |  |
| All | 41 (33; 52) | 38 (30; 48) | 40 (32; 51) | 0,112 | 55, 48, 48 |
| Measured at PEEP ≤ 5 cmH_2_O | 40 (32; 50) | 37 (30; 46) | 39 (31; 49) | 0,113 |  |
| Measured at PEEP 6-10 cmH_2_O | 42 (33; 52) | 39 (31; 50) | 40 (32; 52) | 0,879 |  |
| Measured at PEEP > 10 cmH_2_O | 43 (34; 58) | 41 (31; 53) | 44 (34; 58) | 0,223 |  |
| **Inflammatory markers** |  |  |  |  |  |
| WBC (10^9^/L) | 11.9 (8.9; 16.4) | 12.2 (8.7; 17.2) | 12.0 (8.7; 17.1) | 0,854 | 29, 28, 26 |

| Other parameters |  |  |  |  |  |
| --- | --- | --- | --- | --- | --- |
| Heart rate (bpm) | 84 (72; 96) | 85 (73; 99) | 86 (74; 98) | 0,312 | 12, 9, 11 |
| Temperature (°C) | 37.0 (36.7; 37.3) | 37.0 (36.6; 37.4) | 37.0 (36.6; 37.4) | 0,145 | 23, 24, 22 |
| MAP (mmHg) | 79 (70; 88) | 77 (69; 88) | 78 (70; 87) | 0,567 | 22, 24, 26 |

Table E24: Results of the **after switch analysis**, **distinguishing early and late failures**. Δ_3h_’s of the time-varying variables, distinguishing early and late failures. *P-values are given for the comparison of distributions with early failed (n=1,235) and late failed (n=2,285) switch attempts. Data are mean (SD). PaO_2_=arterial oxygen pressure, PaCO_2_=Partial pressure of carbon dioxide, PEEP= Positive end-expiratory pressure, FiO_2_=Fraction of inspired oxygen, SpO_2_=oxygen saturation, Ppeak=peak airway pressure, RR= respiratory rate, V_t_=tidal volume, DBP=diastolic blood pressure, SBP=systolic blood pressure, MAP=mean arterial pressure.

|  | Successful switch attempts  (n=2,100) | Early failed switch attempts (n=1,235) | Late failed switch attempts (n=2,285) | P value* | Missingness (% successful, % early failed, % late failed) |
| --- | --- | --- | --- | --- | --- |
| **Gas exchange parameters** |  |  |  |  |  |
| Δ_3h_ PaO_2_ (mmHg) | -6.1 (40.8) | -8.1 (48.8) | -8.7 (43.0) | 0,817 | 55, 62, 58 |
| Δ_3h_ PaCO_2_ (mmHg) | 0.2 (5.6) | 2.0 (7.4) | 0.2 (6.1) | <0.001 | 55, 62, 58 |
| Δ_3h_ PF-ratio | -2 (74) | -9 (80) | -13 (93) | 0,536 | 70, 78, 75 |
| Δ_3h_ pH | -0.001 (0.046) | -0.017 (0.06) | -0.001 (0.05) | <0.001 | 63, 67, 66 |
| Δ_3h_ Base excess (mmol/L) | 0.1 (1.6) | -0.2 (2.7) | -0.0 (1.7) | 0,07 | 56, 62, 58 |
| Δ_3h_ FiO_2_ (%) | -1 (9) | -1 (12) | -1 (11) | 0,869 | 31, 54, 43 |
| Δ_3h_ SpO_2_ (%) | 0 (4) | -1 (4) | 0 (4) | 0,208 | 1, 1, 1 |
| **Ventilatory parameters** |  |  |  |  |  |
| Δ_3h_ Ppeak (cmH_2_O) | -3.2 (5.1) | -2.4 (5.7) | -2.8 (4.9) | 0,196 | 33, 56, 46 |
| Δ_3h_ PEEP (cmH_2_O) | -0.4 (1.7) | -0.0 (1.8) | -0.3 (1.6) | 0,006 | 33, 56, 45 |
| Δ_3h_ RR (breaths/min) | -1 (7) | -1 (7) | -1 (7) | 0,374 | 0, 1, 1 |
| Δ_3h_ Minute volume (L/min) | -0.2 (3.7) | -0.3 (2.8) | -0.6 (6.1) | 0,285 | 44, 62, 55 |
| Δ_3h_ V_t_ (mL) | 48 (546) | 27 (379) | 37 (454) | 0,65 | 33, 56, 45 |
| **Other parameters** |  |  |  |  |  |
| Δ_3h_ Heart rate (bpm) | 3 (12) | 3 (14) | 3 (13) | 0,442 | 12, 7, 11 |
| Δ_3h_ Temperature (°C) | 0.1 (0.8) | 0.1 (0.8) | 0.1 (0.7) | 0,565 | 58, 66, 62 |
| Δ_3h_ MAP (mmHg) | 0 (16) | -1 (16) | 0 (15) | 0,161 | 23, 27, 27 |

Table E25: Results of the **predictive analysis**, comparing (1) the originally used LASSO regression with the more flexible Light Gradient Boosting Machine (LightGBM), (2) the originally used KNN-imputation with Sklearn’s IterativeImputer method and (3) limiting the analysis to patients with PaO₂/FiO₂ measurements taken at PEEP levels above 10 cmH₂O. PaO_2_=arterial oxygen pressure, PaCO_2_=Partial pressure of carbon dioxide, PEEP= Positive end-expiratory pressure, HCO_3_^-^ = bicarbonate, FiO_2_=Fraction of inspired oxygen, SpO_2_=oxygen saturation, Pplat=pleateau pressure, ΔP=driving pressure, Pmean=mean airway pressure, Ppeak=peak airway pressure, C_RS_=respiratory system compliance.

|  | Model 1 | Model 2 | |  |
| --- | --- | --- | --- | --- |
|  | **Mean  AUC (IQR)** | | **Mean  AUC (IQR)** | |
| LASSO + KNN imputation (reference;  from Table E5, Appendix D) | 0.59 (0.57 – 0.61) | | 0.61 (0.59 – 0.62) | |
| LightGBM + KNN imputation | 0.57 (0.56 – 0.59) | | 0.59 (0.58 – 0.60) | |
| LASSO + IterativeImputer | 0.59 (0.58 – 0.60) | | 0.61 (0.60 – 0.63) | |
| LASSO + KNN imputation,  considering patients with PaO_2_/FiO_2_ measured during PEEP > 10 cmH_2_O only | 0.59 (0.56 – 0.62) | | 0.61 (0.59 – 0.63) | |

Figure E6: After switch analysis using multiple follow-up times, each separated for early vs late failures (sensitivity analysis) for each time-varying variable. As the follow-up time increases, the likelihood that a new measurement is available between the follow-up moment and the moment of sampling, and hence available for collecting a non-missing Δs, increases. Also, as the follow-up time increases, the number of patients who already failed increases. PaO_2_=arterial oxygen pressure, PaCO_2_=Partial pressure of carbon dioxide, PEEP= Positive end-expiratory pressure, FiO_2_=Fraction of inspired oxygen, SpO_2_=oxygen saturation, Pplat=pleateau pressure, ΔP=driving pressure, Ppeak=peak airway pressure, RR= respiratory rate, DBP=diastolic blood pressure, SBP=systolic blood pressure, MAP=mean arterial pressure.

a) Δ-PaO_2_

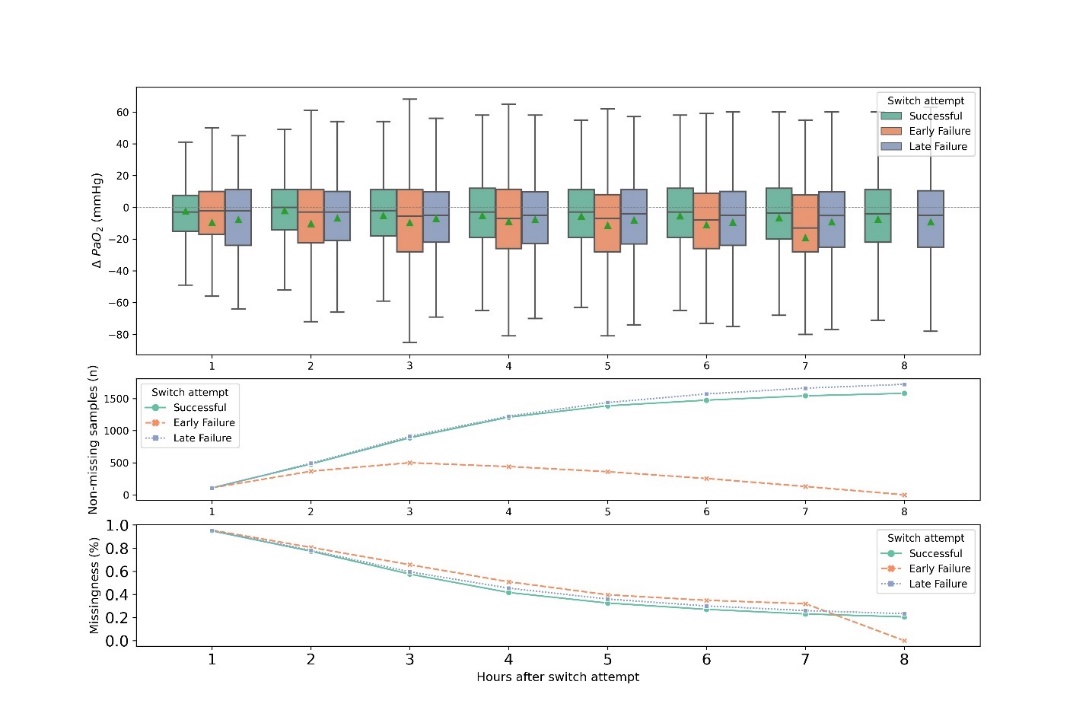


b) Δ-PaCO_2_

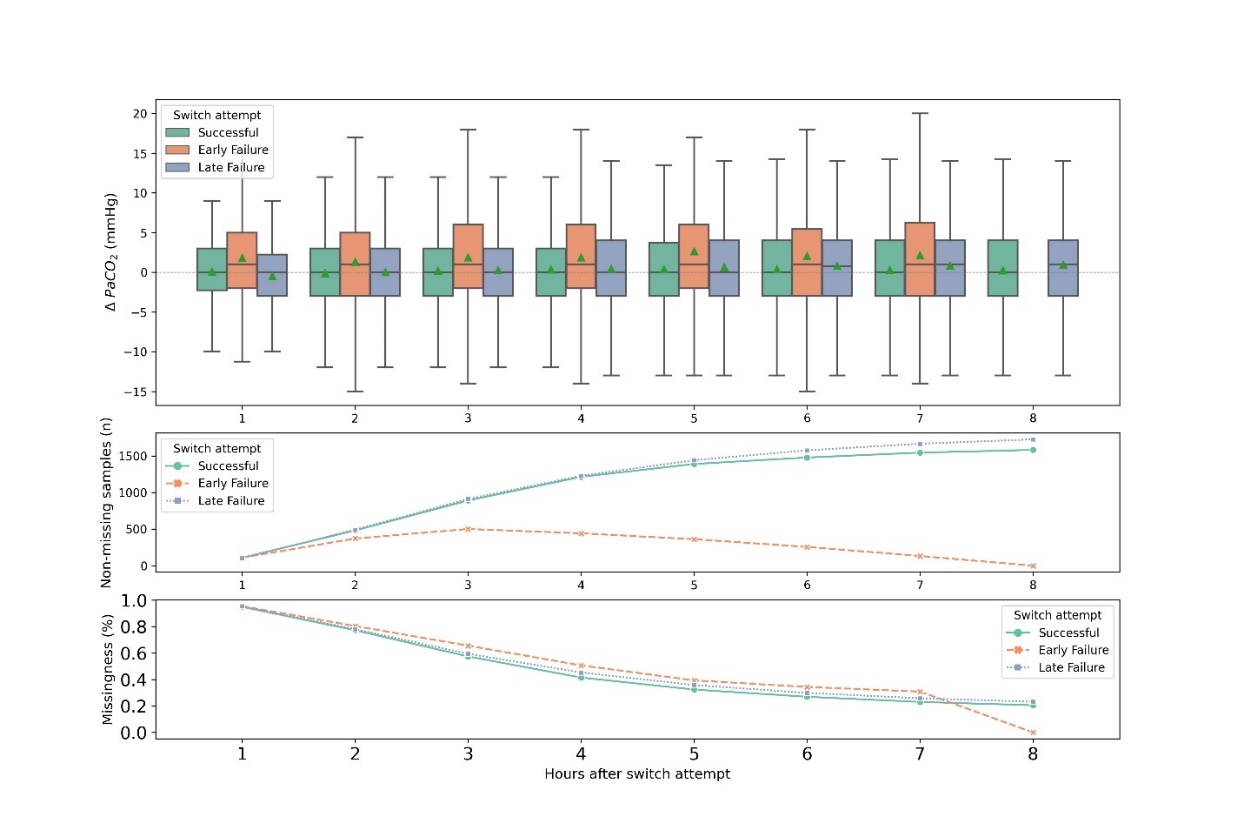


c) Δ-PF-ratio


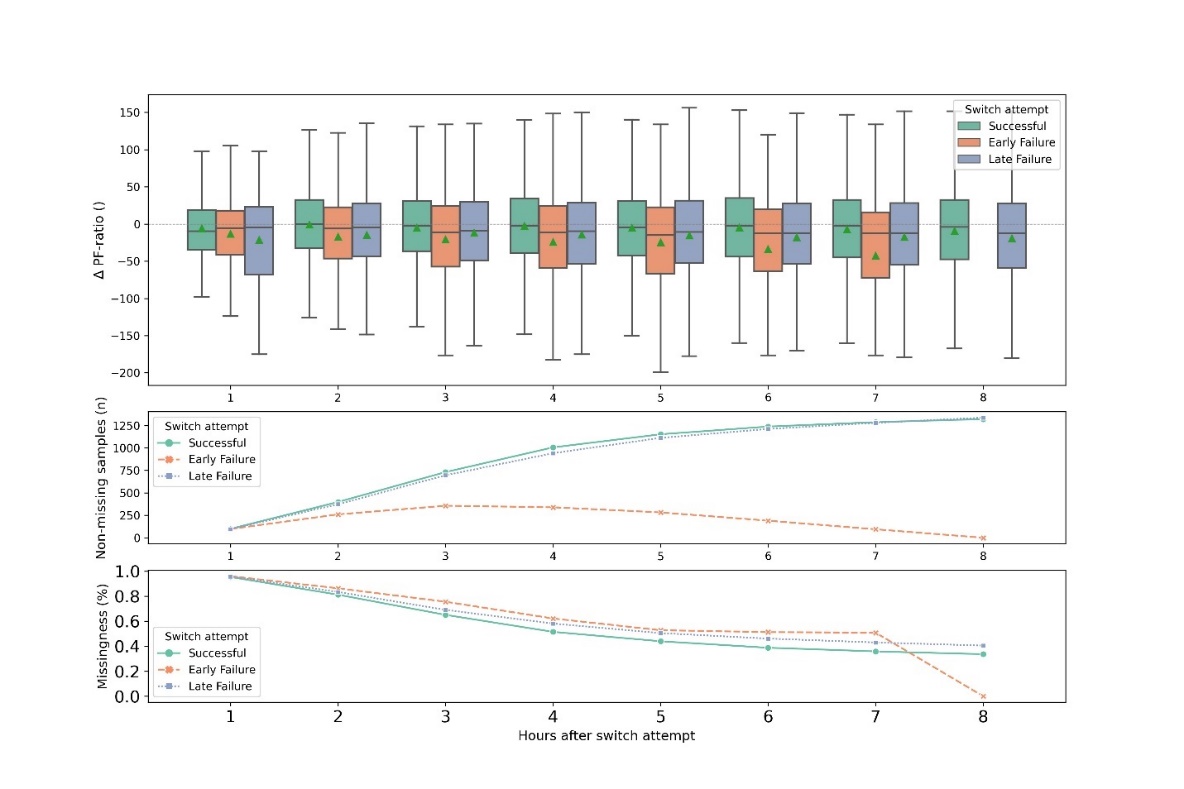


d) Δ-pH


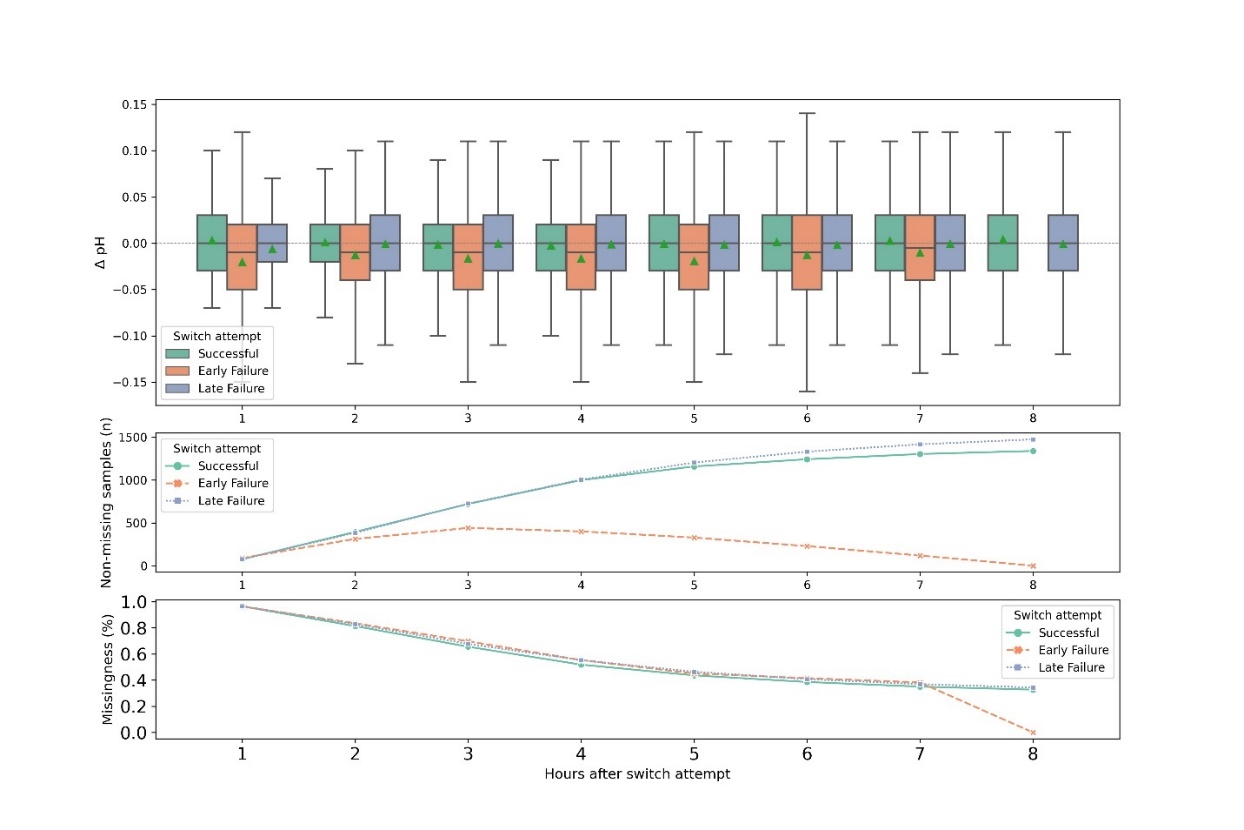


e) Δ-Base excess


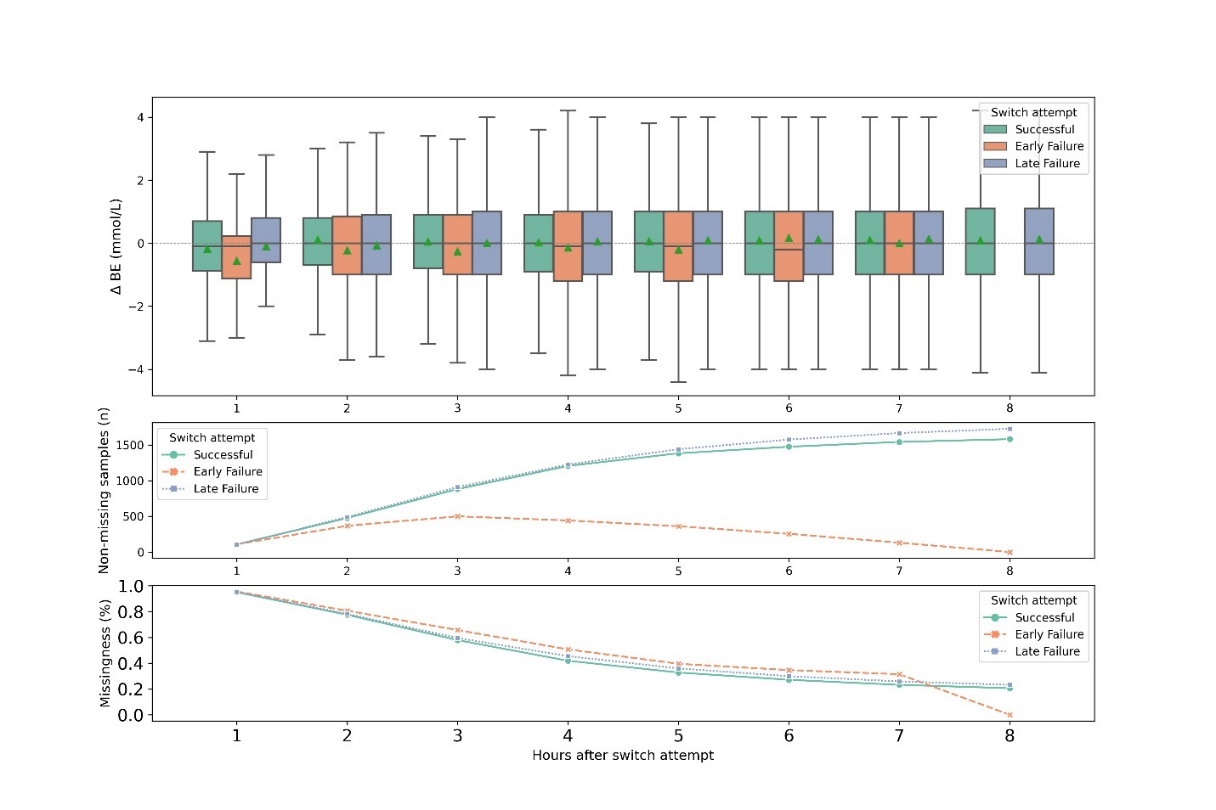


f) Δ-FiO_2_


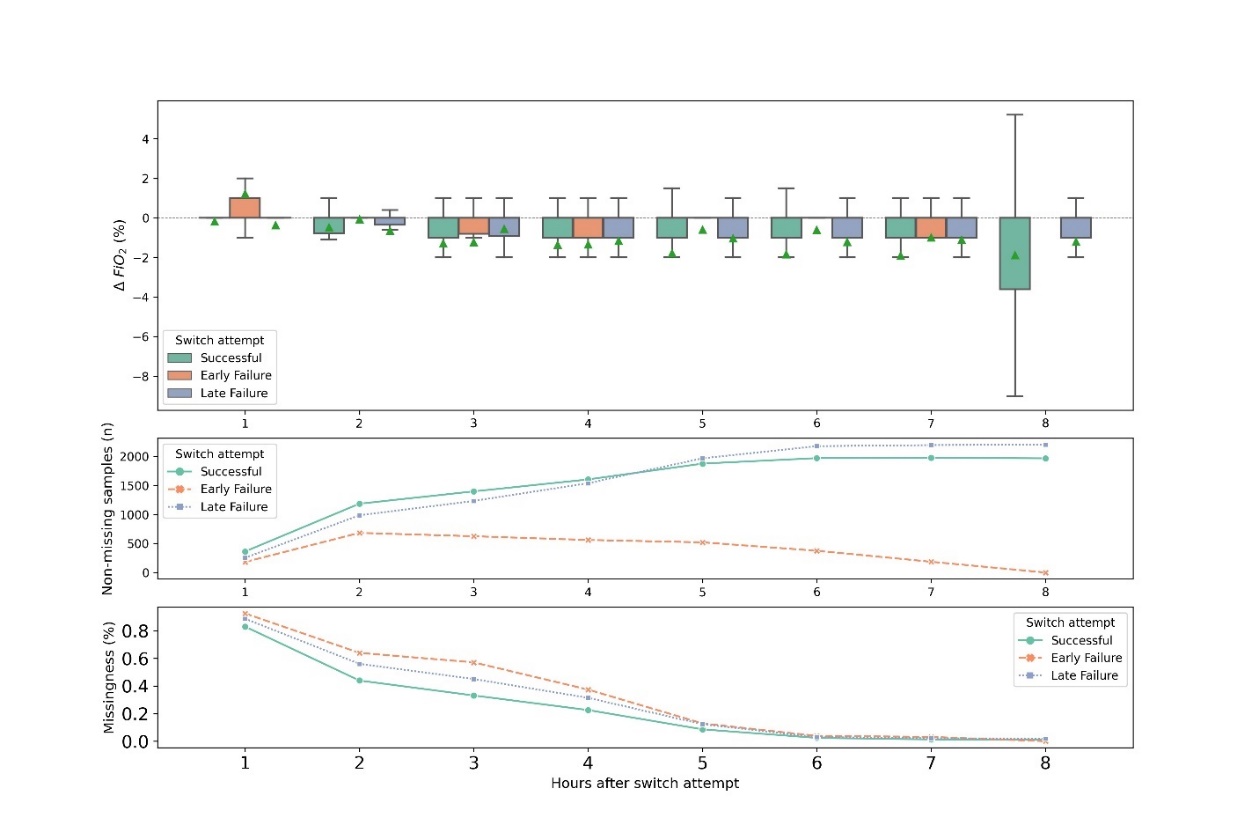


g) Δ-SpO_2_


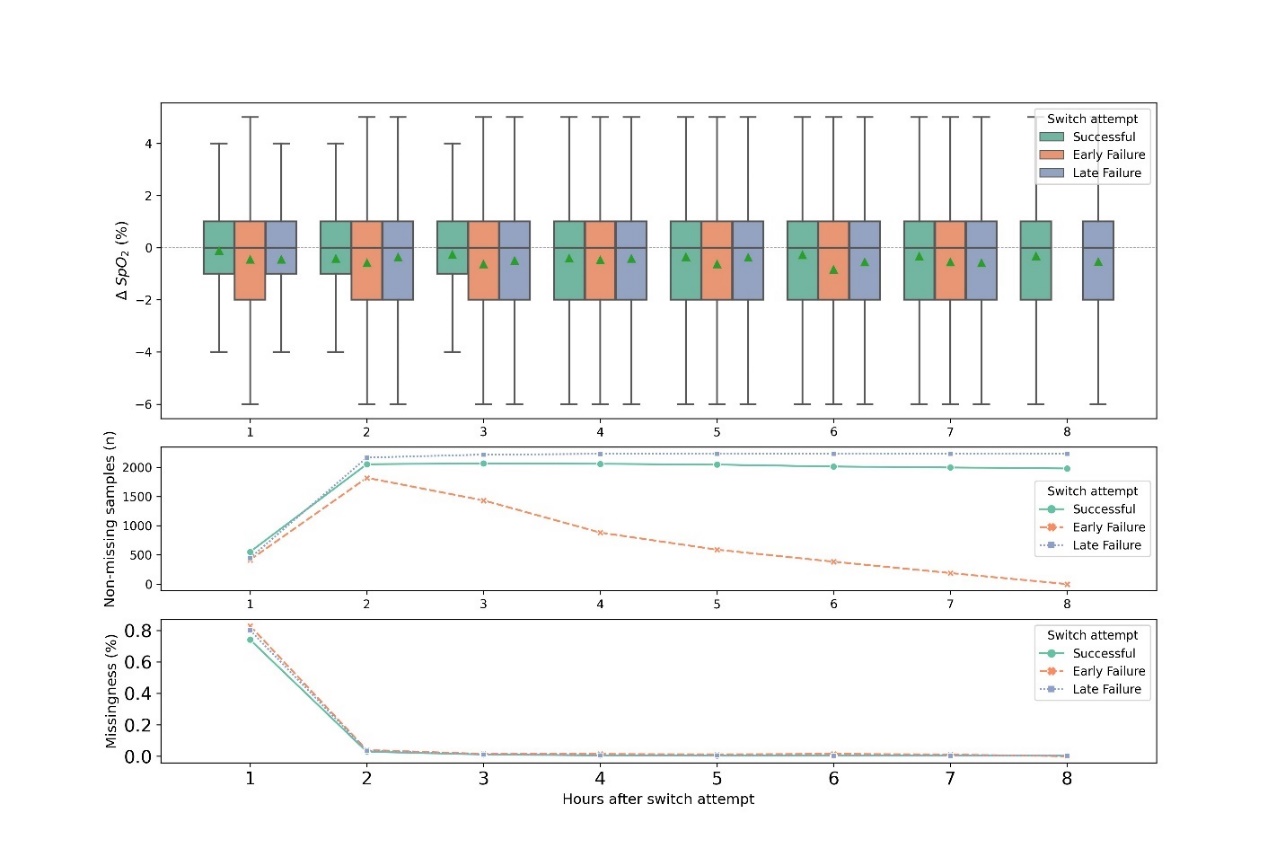


h) Δ-Ppeak


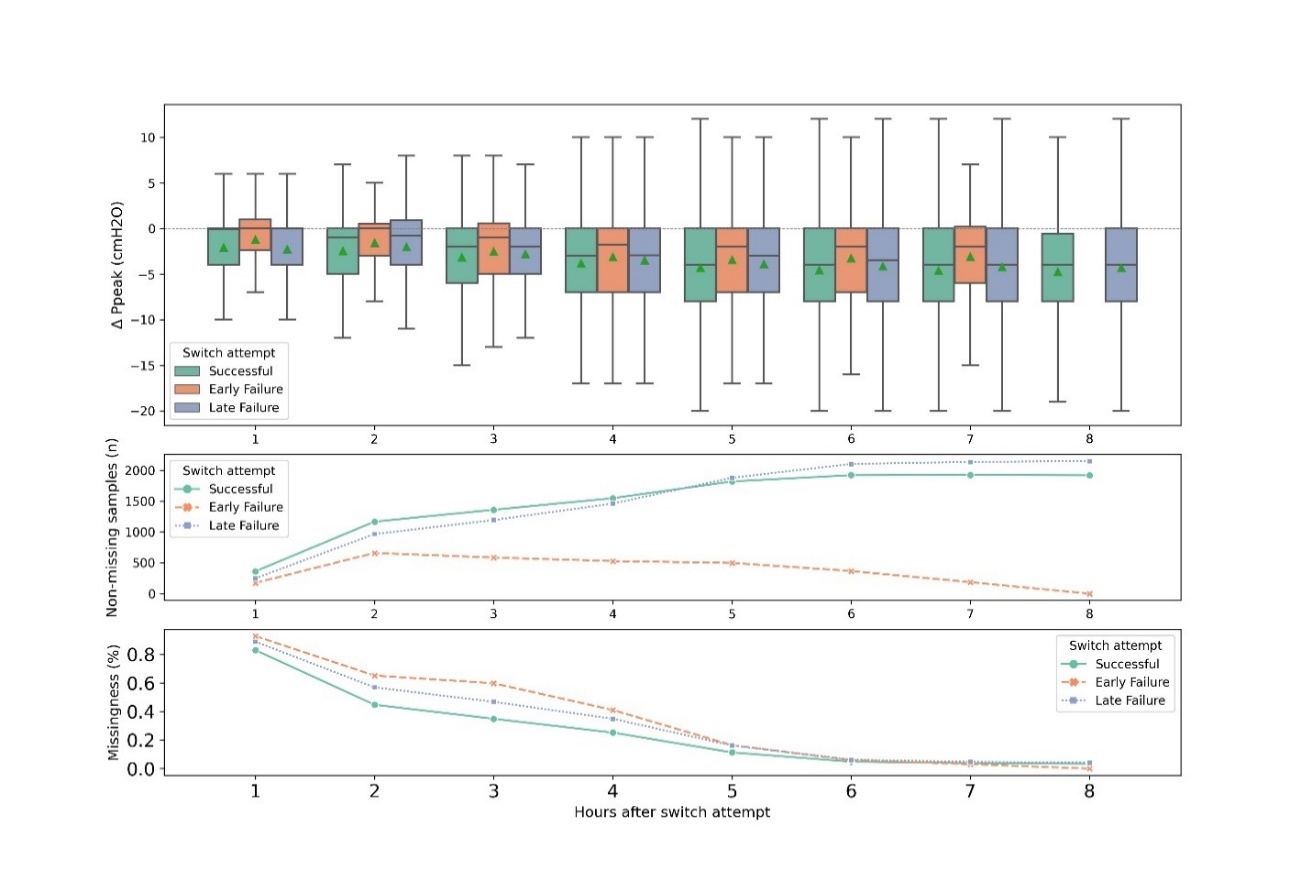


1. Δ-RR


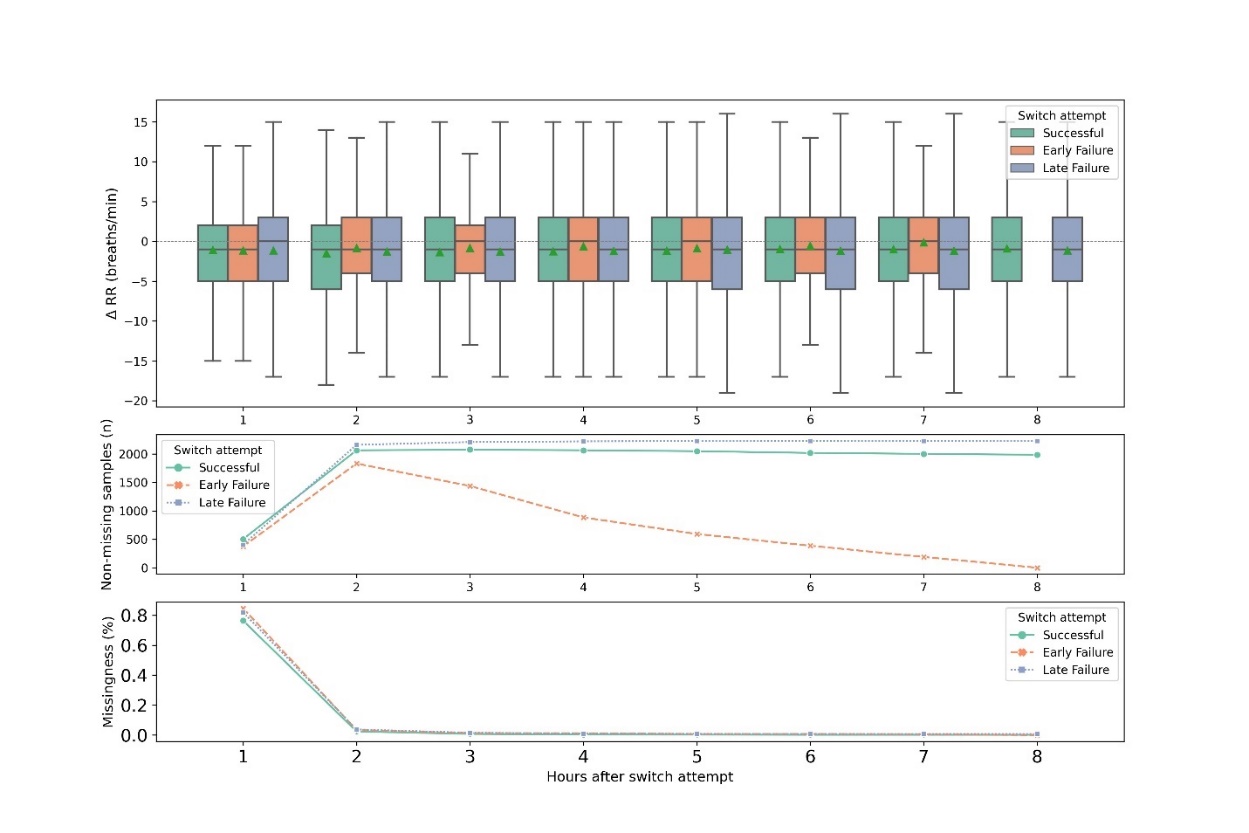


j) Δ-Minute volume


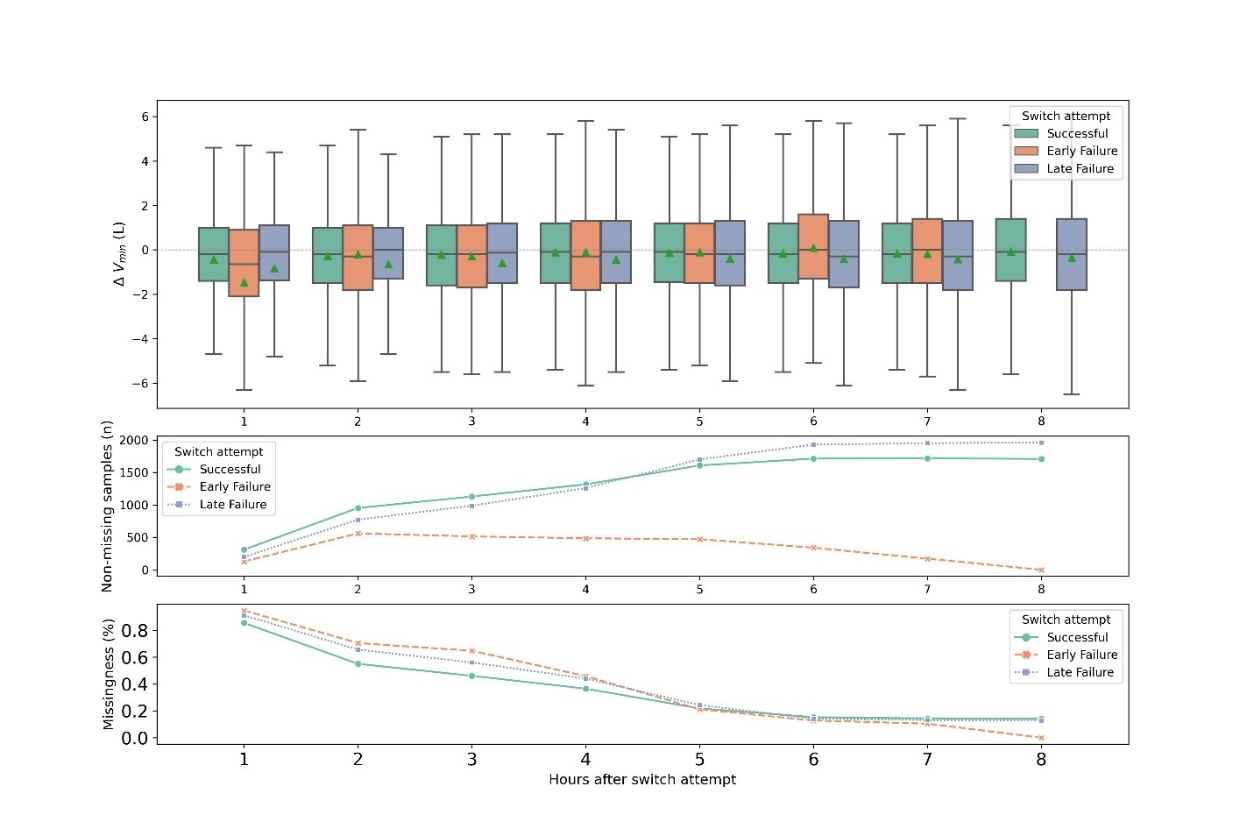


k) Δ-Tidal volume


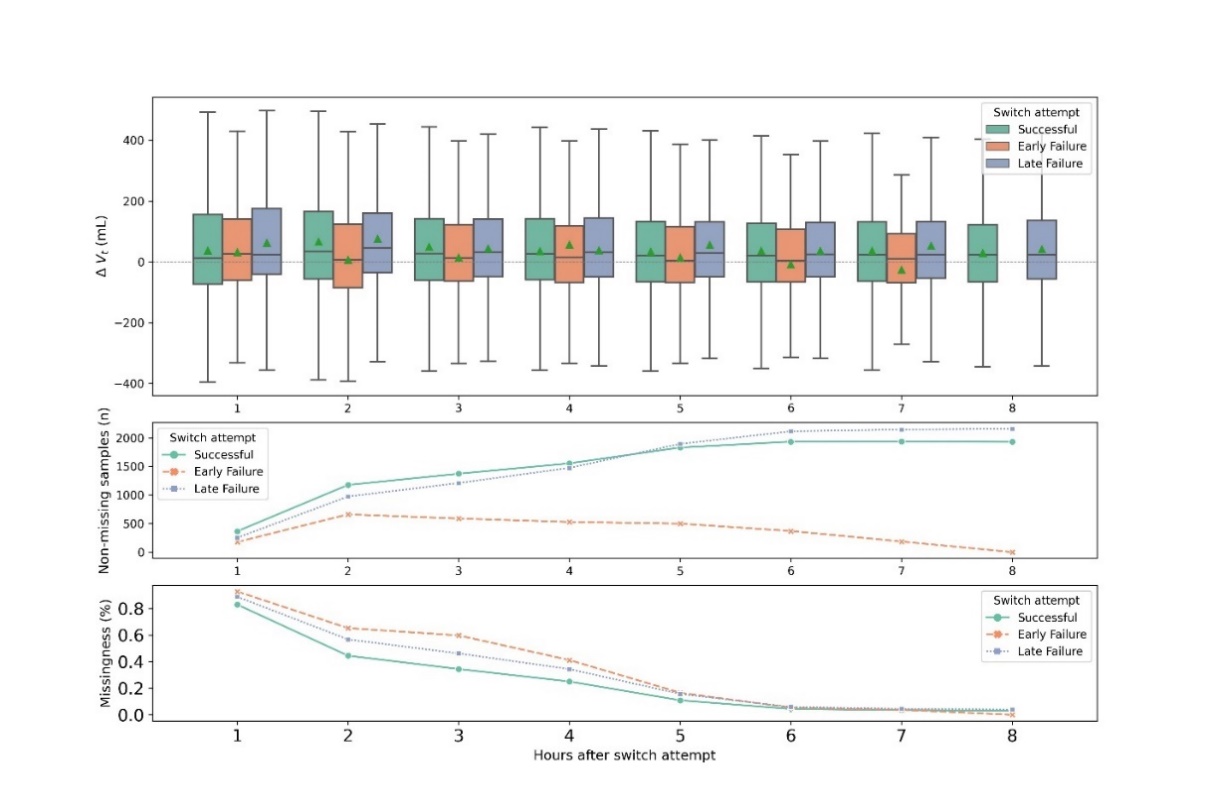


l) Δ-Heart rate


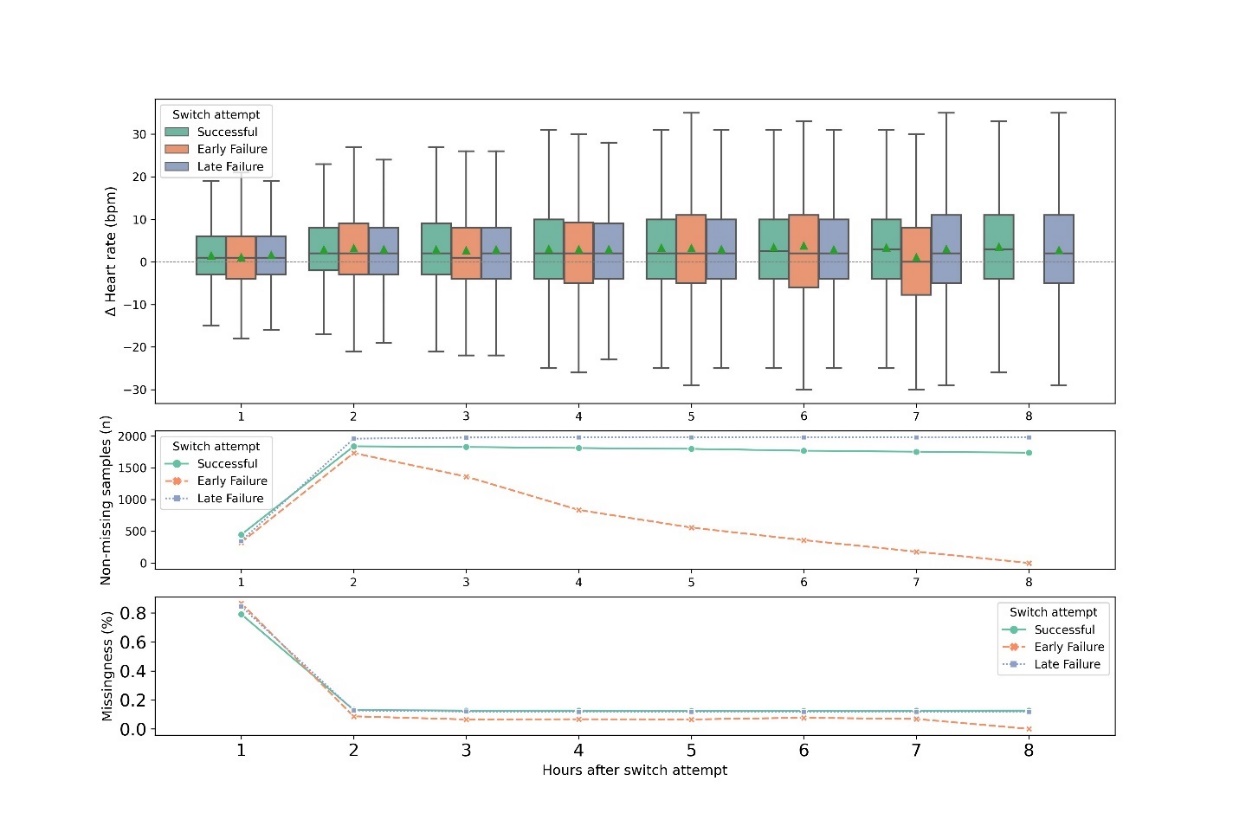


m) Δ-Temperature


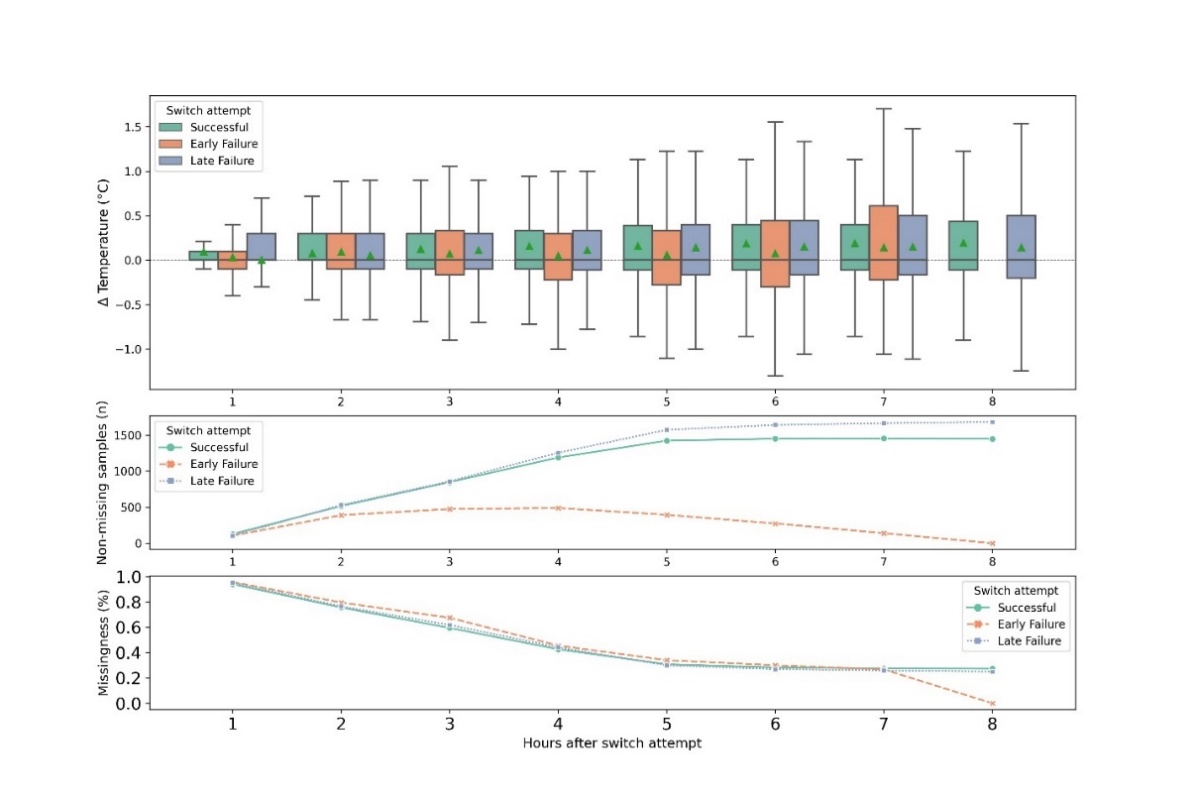


n) Δ-diastolic blood pressure


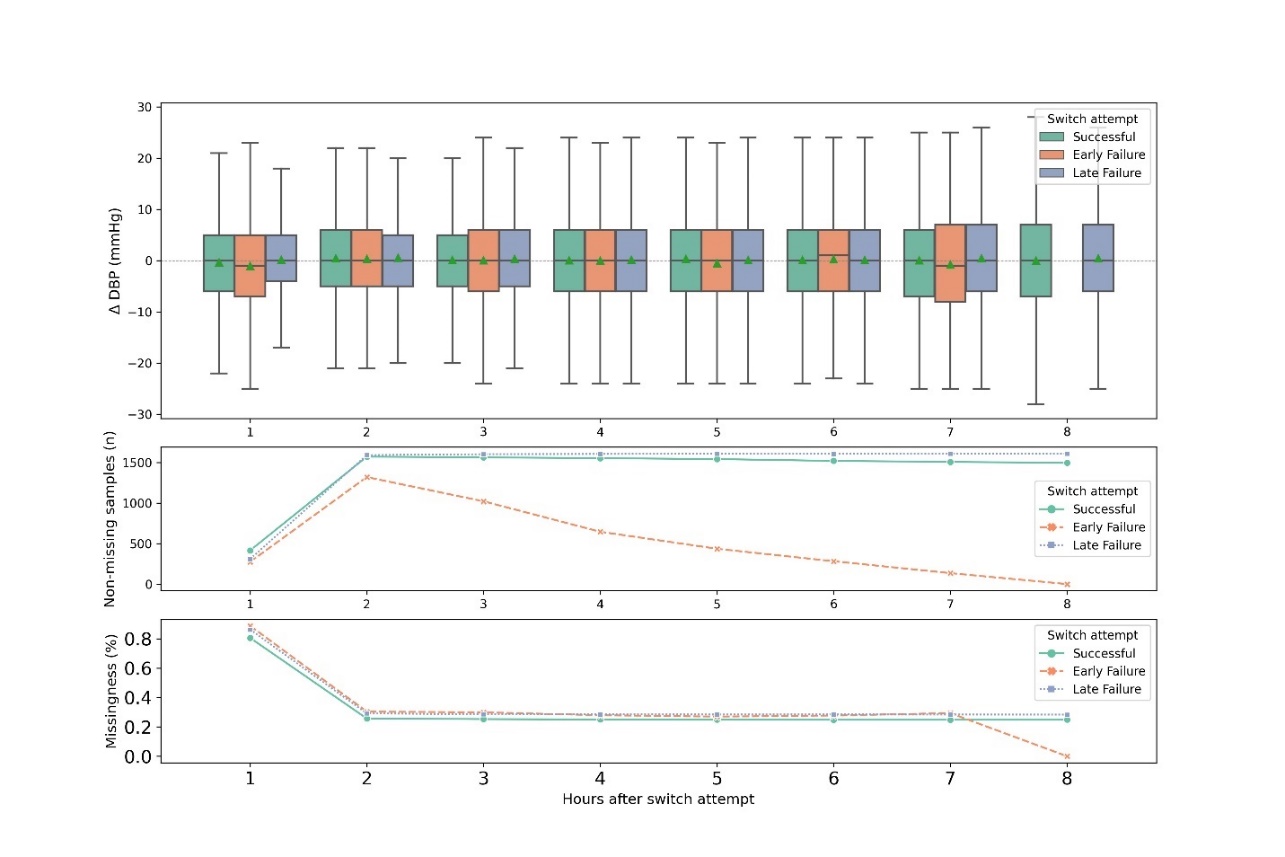


o) Δ-systolic blood pressure


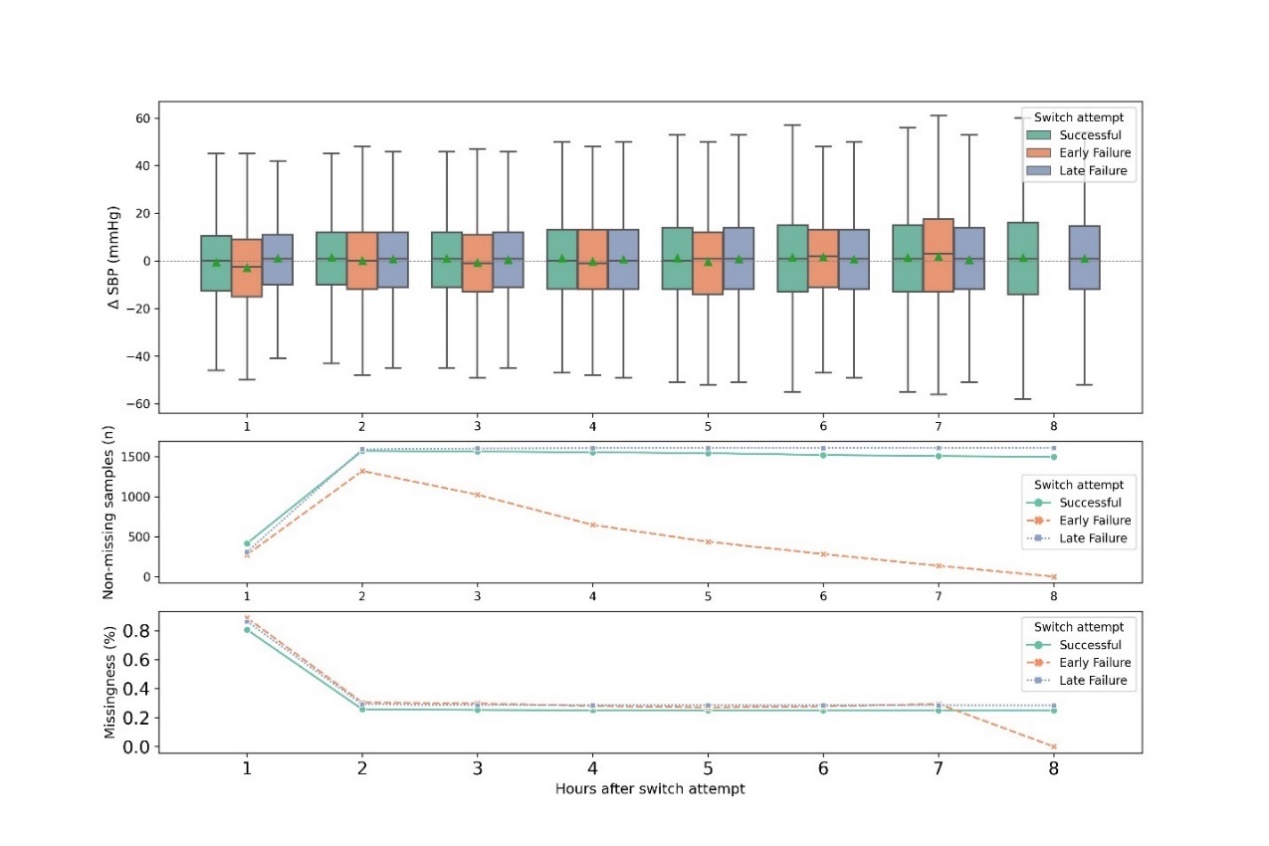


p) Δ-MAP


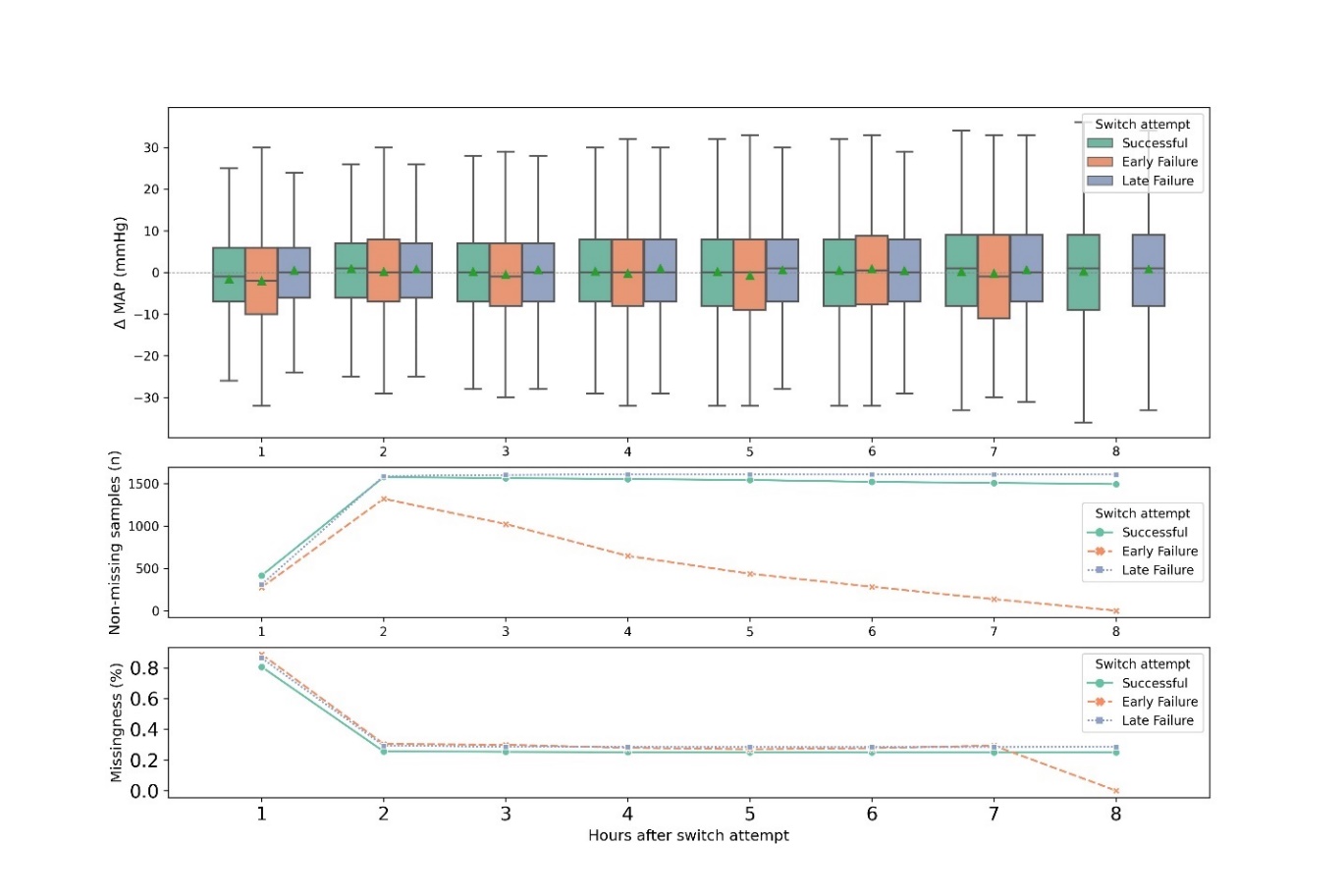

Supplement: Supplementary file 1 — Additional file 1. [file 40635_2025_785_MOESM1_ESM.docx]
